# Abstracts of the 11^th^ International Conference on Cachexia, Sarcopenia and Muscle Wasting, Maastricht, The Netherlands, 7–9 December 2018

**DOI:** 10.1002/jcsm.12365

**Published:** 2018-11-18

**1–01**

**Cross‐sectional associations of n‐6 plasma phospholipid fatty acids with circulating C‐reactive protein and skeletal muscle mass in the EPIC‐Norfolk cohort**

**Richard P.G. Hayhoe** ^1^, Marleen A.H. Lentjes^2^, Angela A. Mulligan^2^, Robert N. Luben^2^, Kay‐Tee Khaw^2^ and Ailsa A. Welch^1^

^1^ *Population Health and Primary Care, Norwich Medical School, University of East Anglia, Norwich, UK;* ^2^ *Public Health and Primary Care, University of Cambridge, Cambridge, UK*

**Introduction:** Dietary fatty acid (FA) intakes have been associated with skeletal muscle mass,^1‐2^ but the importance of n‐6 FAs is underexplored. This study investigated associations of n‐6 plasma phospholipid (PPL) FAs, C‐reactive protein (CRP; a biomarker of inflammation), and fat‐free mass (FFM; a proxy for skeletal muscle mass) in the EPIC‐Norfolk cohort^3^ of individuals aged 39‐79y.

**Methods:** FFM, estimated by bioelectrical impedance analysis, was adjusted for BMI to give a scaled variable, FFM_BMI_. PPLs and serum CRP were measured from peripheral blood. Associations of n‐6 PPLs (linoleic acid (LA), γ‐linolenic acid (GLA), dihomo‐γ‐linolenic acid (DGLA), and arachidonic acid (AA); each as a percentage of total PPLs) and CRP (mg/L), with muscle mass were investigated in individual logistic regression models. The odds ratio (OR) of each sex‐specific quintile of PPL or CRP was calculated in relation to membership of a ‘low muscle mass’ group (defined as individuals with the lowest 10% FFM_BMI_). Linear regression was used to test associations of n‐6 PPLs with CRP.

**Results:** The OR of being in the low muscle mass group was lower in higher quintiles of LA for both sexes. Conversely, odds of being in the low muscle mass group were higher in DGLA quintile 5 *vs*. quintile 1 in men, and quintiles 2 and 4 in women; and AA quintiles 3 to 5 *vs*. quintile 1 in women. Higher CRP quintiles were also associated with higher odds of being in the low muscle mass group. CRP was negatively associated with LA in men, and positively associated with DGLA in men and women.

All models adjusted for: age, smoking status, physical activity, social class, menopausal and HRT status in women, statin use, and corticosteroid use. ^1^ Odds ratios are relative to quintile 1.^2^ Men *n* = 919, women *n* = 822. Associations p < 0.05 are shown in bold.

**Conclusions:** In this cohort, specific n‐6 PPLs and serum CRP are strong predictors of low skeletal muscle mass. Specific n‐6 PPLs are also associated with CRP. Further investigation is required into the interplay of these factors, and the implications for skeletal muscle health.

**Table 1:** 

**PPL or CRP**		**Quintile 2**			**Quintile 3**			**Quintile 4**			**Quintile 5**		
		OR^1^	CI	P	OR	CI	P	OR	CI	P	OR	CI	P
**Men**	LA	0.7	0.4–1.2	0.177	**0.5**	**0.3–0.8**	**0.011**	**0.4**	**0.2–0.8**	**0.007**	**0.3**	**0.2–0.6**	**0.001**
*n* = 1167	GLA	1.3	0.7–2.4	0.397	1.2	0.6–2.2	0.564	1.0	0.5–1.9	0.991	1.6	0.9–2.9	0.132
	DGLA	1.9	1.0–3.9	0.060	1.8	0.9–3.6	0.111	1.8	0.9–3.7	0.095	**3.3**	**1.7–6.3**	**<0.001**
	AA	0.8	0.4–1.6	0.558	1.4	0.8–2.4	0.301	1.0	0.5–1.8	0.927	1.0	0.5–1.9	0.994
	CRP^2^	2.1	0.6‐6.9	0.237	**5.1**	**1.7–15**	**0.004**	**6.2**	**2.0–19**	**0.001**	**7.2**	**2.5–21**	**<0.001**
**Women**	LA	**0.5**	**0.3–0.9**	**0.012**	**0.4**	**0.2–0.8**	**0.004**	**0.4**	**0.2–0.7**	**0.002**	**0.2**	**0.1–0.5**	**<0.001**
*n* = 980	GLA	1.3	0.6–2.7	0.505	1.9	0.9–3.7	0.079	1.8	0.9–3.6	0.100	1.6	0.8–3.3	0.172
	DGLA	**2.3**	**1.1–4.8**	**0.035**	2.0	0.9–4.4	0.069	**2.8**	**1.3–5.9**	**0.007**	2.0	0.9–4.4	0.071
	AA	0.9	0.4–2.0	0.830	**2.1**	**1.0–4.2**	**0.039**	**2.0**	**1.0–4.1**	**0.046**	**2.3**	**1.1–4.5**	**0.020**
	CRP	2.5	0.5–12	0.252	**5.0**	**1.1–23**	**0.037**	**9.7**	**2.2–42**	**0.002**	**11**	**2.5–47**	**0.001**

1. Welch A, MacGregor A, Minihane A, *et al.* (2014) *J Nutr* 144 (3), 327–34.

2. Hayhoe R, Lentjes M, Mulligan A, *et al.* (2017) *Proc Nut Soc* 76, E127

3. Day N, Oakes S, Luben R, et al. (1999) *Br J Cancer* 80, 95–103.

**1–02**

**Comparison of muscle fatigability measured by Martin Vigorimeter and Jamar Dynamometer**

**Liza De Dobbeleer** ^1,2^, David Beckwée^1,3,4,5^, Pauline Arnold^1,5^, Stéphane Baudry^6^, Ingo Beyer^1,2^, Jeroen Demarteau^1,5^, Ynes Punie^1,5^ and Ivan Bautmans^1,2,5^

^1^ *Gerontology Department and Frailty in Aging Research (FRIA) Group, Faculty of Medicine and Pharmacy, Vrije Universiteit Brussels, Belgium;* ^2^ *Departement of Geriatrics, Universitair Ziekenhuis Brussels, Belgium;* ^3^ *Rehabilition Sciences Research Department, Vrije Universiteit Brussels, Belgium;* ^4^ *Department of Rehabilitation Sciences and Physiotherapy, Faculty of Medicine and Health Sciences, University of Antwerp, Belgium;* ^5^ *SOMT University of Physiotherapy, Amersfoort, The Netherlands;* ^6^ *Faculté des Sciences de la Motricité, Brussels, Belgium*

**Introduction:** Recently, we have introduced, refined and validated the fatigue resistance (FR) test ‐defined as the time during which grip strength drops to 50% of its maximum during sustained contraction‐ to objectively measure the exhaustion component of Frailty. The test has been validated for the Martin Vigorimeter (MV). However, many researchers and clinicians are using the Jamar Dynamometer (JD). To date, scarce data regarding the FR test measured with the JD are available, thus limiting its implementation.

**Methods:** 618 young, healthy controls (304♂ and 314♀, aged 18–29 years), 660 community‐dwelling adults (310♂ and 350♀, aged 30–95 years) and 50 hospitalized patients (25♂ and 25♀, aged 70–99 years) participated. FR of the dominant hand was recorded with MV and JD, on the same day in a random order with at least 1 hour interval between both tests.

**Results:** FR scores differed significantly between both handgrip devices, with FR measured by MV (55.7 ± 35.0 s) being significantly (*p* < 0.001) higher than when measured by JD (34.2 ± 18.4 s). When FR scores were studied for men (mean difference = 18.5 ± 29.3 s, 95% confidence interval [16.2–20.8]) and women (mean difference = 24.4 ± 34.1 s, 95% confidence interval [21.8–27.0]) separately, similar results were found. In addition, this difference remained present when taking into account the clinical condition and sex. Moreover, Bland–Altman plots show the difference between both devices increases with higher FR scores, highlighting that the longer the participant could sustain the FR test, the higher the difference in FR measured with both devices. This was seen for all participants (R^2^=0.364, *p* < 0.001) and for men (R^2^=0.305, p < 0.001) and women (R^2^=0.402, p < 0.001) separately.

**Conclusions:** FR scores obtained with MV and JD are not interchangeable. Our results are in line with our previous finding that JD is unable to identify subjects with higher levels of muscle endurance, suggesting that the MV might be more suitable for measuring muscle FR.

**1–03**

**Grip work measurement with the Jamar Dynamometer: validation of a simple equation for clinical use**

**Liza De Dobbeleer** ^1,2^, Ingo Beyer^1,2^, Hansen Åse Marie^3,4^, Drude Molboe^3^, Erik L. Mortensen^4,5^, Rikke Lund^3,4^ and Ivan Bautmans^1,2^

^1^ *Gerontology Department and Frailty in Aging Research (FRIA) Group, Faculty of Medicine and Pharmacy, Vrije Universiteit Brussels, Belgium;* ^2^ *Department of Geriatrics, Universitair Ziekenhuis Brussels, Belgium;* ^3^ *Section of Social Medicine, Department of Public Health, University of Copenhagen, Denmark;* ^4^ *Centre for Healthy Aging, University of Copenhagen, Denmark;* ^5^ *Section of Environmental Health Department of Public Health, University of Copenhagen, Denmark*

**Introduction:** Increased muscle fatigability might explain the occurrence of fatigue, one of the major characteristics of frailty in older persons. Surprisingly, resistance to fatigue is not commonly evaluated during geriatric assessment, possibly because older patients are often unable to perform the classic endurance tests. Previously, we developed and validated an easy test to measure of muscle fatigability during sustained maximal handgrip contraction in older persons using a Martin Vigorimeter (MV) device. This study aimed at validating the equation to estimate grip work (GW) during sustained maximal handgrip contraction, by monitoring continuously the grip strength (GS) decay using a Jamar Dynamometer‐like (JD) device.

**Methods:** Data collection took place at The National Research Centre for the Working Environment in Copenhagen, Denmark. 962 subjects, belonging to a subgroup of the Copenhagen Aging and Midlife Biobank, were enrolled. GS was recorded continuously during sustained maximal contraction until it dropped to 50% of its maximum and fatigue resistance (FR, time to fatigue) was noted. GW, area under the force‐time curve, was compared to its estimate which was calculated as GW_estimated_ = GS_max_*0.75*FR.

**Results:** Excellent correlation was found between GW_estimated_ and GW_measured_ (R^2^=0.98, *p* < 0.001). The equation slightly overestimated GW by 6.04 kg*s (95% confidence interval [−0.08–12.15]) with a coefficient of variation method error of 6%.

**Conclusions:** GW estimation is a valid parameter reflecting muscle work output during a sustained maximal grip effort in healthy middle‐aged community‐dwelling persons when using a JD.

**1–04**

**Increased myonuclear accretion during recovery from skeletal muscle metabolic stress**

**Anita E.M. Kneeppers** ^1^, Lex B. Verdijk^2^, Marco C.J.M. Kelders^1^, Mitja Lainscak^3,4^, Luc J.C. van Loon^2^, Annemie M.W.J. Schols^1^ and Ramon C.J. Langen^1^

^1^ *Department of Respiratory Medicine, NUTRIM School of Nutrition and Translational Research in Metabolism, Maastricht University Medical Centre+, Maastricht, the Netherlands;* ^2^ *Department of Human Biology, NUTRIM School of Nutrition and Translational Research in Metabolism, Maastricht University Medical Centre+, Maastricht, the Netherlands;* ^3^ *Division of Cardiology, General Hospital Murska Sobota, Murska Sobota, Slovenia;* ^4^ *Faculty of Medicine, University of Ljubljana, Ljubljana, Slovenia*

**Rationale:** It has been well‐established that sustained overload‐induced hypertrophy upon resistance‐type exercise training requires an intact satellite cell response. It was recently suggested that satellite cells may also play an important role in non‐hypertrophic skeletal muscle adaptations and remodelling upon endurance‐type exercise training, although the underlying triggers and regulatory mechanisms remain elusive. We hypothesized that FGF21 is a myotube‐derived regulator of myogenesis‐associated myonuclear accretion in response to skeletal muscle metabolic stress and tested this in a translational approach.

**Methods**: Changes in FGF21 mRNA expression were correlated to skeletal muscle mass‐ and metabolic plasticity related processes, measured in biopsies of the *M. Vastus Lateralis* of patients with COPD, obtained before and after 4 weeks of exercise training. A novel *in vitro* model of postnatal myogenesis was used to investigate the regulation of FGF21 mRNA expression and myonuclear accretion upon myotube metabolic stress.

**Results:** The exercise training‐induced changes in FGF21 mRNA expression correlated with the *in vivo* Myogenesis response (r_s_ = 0.375, *p* < 0.05). *In vitro*, recovery from myotube metabolic stress or atrophy increased myogenic fusion‐dependent luciferase activity (all *p* < 0.001). Only during recovery from metabolic stress, this was accompanied by AMPK activation (pAMPK/AMPK, p < 0.05) and a tendency towards increased FGF21 mRNA expression (*p* < 0.1). Furthermore, myotube FGF21 knockdown decreased subsequent fusion‐dependent luciferase activity (*p* < 0.01).

**Conclusions:** The current study shows that recovery from myotube metabolic stress is accompanied by increased myonuclear accretion, and suggests that AMPK/FGF21 signalling mediates this metabolic regulation of myonuclear accretion.

**1–05**

**The RePOWER registry: a global prospective observational study of patients with primary mitochondrial myopathy**

**Michelangelo Mancuso** and on behalf of the RePOWER Investigators

*University of Pisa, Pisa, Italy*

**Introduction:** Primary mitochondrial myopathies (PMM) are genetic disorders impacting the mitochondrial respiratory chain affecting predominantly, but not exclusively, skeletal muscle. PMM is associated with fatigue, exercise intolerance, and muscle weakness, all of which are commonly reported symptoms adversely affecting physical function and quality‐of‐life. RePOWER is integral to the elamipretide development program, the MMPOWER Program, and designed to capture demographic, genetic and functional data from participants at 29 global research sites. RePOWER compliments the clinical phase I (MMPOWER‐1) and phase II (MMPOWER‐2) trials, all of which supported the development of the pivotal phase III trial, MMPOWER‐3.

**Objectives:** RePOWER is designed to evaluate the genotypes and phenotypes identified in patients with PMM, and the regional differences in how patients with PMM are managed. RePOWER also identified potential participants for MMPOWER‐3.

**Methods:** This global prospective, non‐interventional study enrolled ambulatory patients 16–80 years with signs and/or symptoms associated with PMM. Enrolled patients completed a single visit in which all demographic, genetic, and clinical/functional assessments were performed and collected.

**Results:** Preliminary analyses were conducted on 414 registered participants. Average participant age was 43.6 years with the majority being white (92%) and female (60%). Greater than 87% of participants had genetic testing performed prior to registry entry. Ongoing classification of genetic defects shows 77% (230/300) as “mtDNA mutations that impair mitochondrial protein synthesis in toto”, while nDNA mutations and multiple mtDNA deletions accounted for the remaining 23% (70/300). Three functional tests were performed during the assessment visit. The 6‐Minute Walk Test average distance was 367.8 meters, while the 5‐Times Sit‐to‐Stand Test and Triple‐Timed‐Up‐and‐Go Test, had average times‐to‐completion of 20.0 seconds and 42.4 seconds, respectively.

**Conclusions:** The RePOWER population demonstrated a significant level of disease burden when compared to literature‐based healthy controls. Additionally, the data demonstrated regional differences in the handling of patients with PMM.

**1–06**

**Inactivation of GSK‐3β potentiates PGC‐1α‐mediated mitochondrial biogenesis in skeletal muscle cells**

**Wessel F. Theeuwes** ^1^, Harry R. Gosker^1^, Ramon C.J. Langen^1^, Koen J.P. Verhees^1^, Nicholas A.M. Pansters^1^, Annemie W.J. Schols^1^ and Alexander H.V. Remels^2^

^1^ *NUTRIM School of Nutrition and Translational Research in Metabolism, Departments of Respiratory Medicine;* ^2^ *Pharmacology and Toxicology, Maastricht University Medical Center+, Maastricht, The Netherlands*

**Background:** Aberrations in oxidative metabolism of the skeletal musculature is a debilitating feature often observed in chronic diseases, such as *e.g*. chronic obstructive pulmonary disease (COPD). Muscle mitochondrial biogenesis and oxidative metabolism are tightly controlled by the peroxisome proliferator‐activated receptor‐γ co‐activator 1 (PGC‐1) signalling network, which has been shown to be disturbed in the musculature of COPD patients. Inactivation of glycogen synthase kinase (GSK)‐3β was previously shown to potently increase PGC‐1α abundance in non‐muscle cells.

**Research Question:** Does inactivation of GSK‐3β enhance mitochondrial content and function in cultured muscle cells via upregulation of *Pgc‐1α* and, if so, what is the underlying molecular mechanism?

**Methods:** GSK‐3β was inactivated genetically or pharmacologically during myogenic differentiation or in fully differentiated C2C12 muscle cells. Subsequently, key parameters of mitochondrial oxidative metabolism as well as expression levels of constituents of the PGC‐1 signalling network and known regulators of this pathway were investigated.

**Results:** Inactivation of GSK‐3β increased *Pgc‐1α* gene expression both during myogenic differentiation (4‐fold) and in fully differentiated C2C12 myotubes (10‐fold). Increased *Pgc‐1α* expression was associated with enhanced development of mitochondrial biogenesis and increased myotube oxidative capacity. Our preliminary data shows that myocyte enhancer factor (MEF)2 and oestrogen‐related receptor (ERR) α, transcription factors known to bind and activate the PGC‐1α promoter, are likely not responsible for the induction of *Pgc‐1α* following inactivation of GSK‐3β.

**Conclusions:** We show a novel interaction between inactivation of the GSK‐3β protein, well‐known to be involved in muscle mass regulation, and regulation of mitochondrial biogenesis via the *Pgc‐1α* signalling network in muscle cells. This highlights an intricate link between pathways involved in regulation of skeletal muscle energy production and those controlling muscle mass. However, elucidating the molecular basis how inactivation of GSK‐3β upregulates *Pgc‐1α* gene expression warrants further attention.

**1–10**

**Magnetic Resonance Fingerprinting for sarcopenic skeletal muscle multi‐parametric tissue characterization**

**Benjamin Marty**, Harmen Reyngoudt and Pierre G. Carlier

*Institut de Myologie, AIM & CEA, Neuromuscular Investigation Center, NMR Laboratory, Paris, France*

**Introduction:** Nuclear Magnetic Resonance (NMR) is an extremely versatile imaging modality to characterize skeletal muscle tissue structure. In relation with sarcopenia, it can precisely measure individual muscle volumes and their degree of fatty replacement, with intramuscular fat fraction (FF) maps. Quantitative water T1 (T1H2O) mapping, which is routinely used to monitor inflammation, necrosis or fibrosis processes in myocardial tissues, is another potential tool to characterize muscle aging. Here, we proposed and validated an ultra‐fast sequence, inspired by the Magnetic Resonance Fingerprinting (MRF) method, allowing simultaneous estimation of T1H2O and FF in skeletal muscles.

**Methods:** The MRF sequence for water and fat separation (MRF‐WF) consisted in the acquisition of a long golden angle radial echo‐train, following non‐selective inversion. The echo time, repetition time and flip angle were varied throughout the sequence and the acquisition time was 10 sec per slice. Apparent B1, FF and T1H2O were computed using Bloch‐equations‐based modelling of the MRF signal. It was validated on a multi‐vial phantom, containing emulsions at different FF and T1H2O values. Reference T1H2O and FF were separately measured within each vial using MR spectroscopy and Dixon sequences. Proof‐of concept in vivo acquisitions were performed on healthy volunteers and patients suffering from different neuromuscular disorders.

**Results:** T1H2O and FF values estimated with MRF‐WF correlated with the reference values (R2 = 0.99). Variations of FF and T1H2O were visible in several muscles groups of the patients and MRF‐FF values correlated with Dixon‐FF (R2 = 0.97).

**Conclusions:** The MRF‐WF sequence allowed simultaneous estimation of unbiased T1H2O and FF values in voxels containing a mixture of water and fat with a short acquisition time compatible with whole‐body imaging. It could be acquired on large cohorts of elderly subjects to better understand the relationship between muscle loss, fibre typology, fibrosis and inflammation processes in sarcopenia.

**1–11**

**Bioelectrical impedance analysis to predict outcomes in hemodialysis patients**

**Soo Jeong Choi** ^1^, Min Sung Lee^1^, Seok Hui Kang^2^, Su‐Hyun Kim^3^, Sujung Park^1^, Byung Chul You^1^, Moo Yong Park^1^, Jin Kuk Kim^1^ and Seung Duk Hwang^1^

^1^ *Division of Nephrology, Department of Internal Medicine, Soonchunhyang University Bucheon Hospital;* ^2^ *Division of Nephrology, Department of Internal Medicine, Yeungnam University Hospital, Daegu, Republic of Korea;* ^3^ *Division of Nephrology, Department of Internal Medicine, Chung‐Ang university Hospital, Seoul, Korea*

**Background:** Overhydration and sarcopenia in patients with end stage renal disease (ESRD) are associated with all‐cause mortality and cardiovascular events, while obesity is associated with better survival. Although ESRD patients on hemodialysis check body weight every session, it is not easy to check the change of body composition. Our aim was to evaluate the effect of body composition on survival and hospitalization during maintained HD period.

**Methods:** We enrolled trice weekly HD patients and assigned them to get body composition test using bio‐impedance analysis. We followed up their body composition and outcomes for 2 years.

**Results:** Total 114 HD patients (56.2 ± 13.3 years old, 58 males) were enrolled. Forty eight (42%). patients were diabetic. At baseline, BMI and appendicular skeletal muscle index (ASMI) was 22.2 (15.5~34.1) and 8.5 kg/M^2^ (4.1–13.7), respectively. So obesity (>25 Kg/M2) and sarcopenia (ASMI <7.0 kg/m^2^; male or < 5.7 kg/m^2^; female) was 19.4% and 41.6%, respectively. Thirty two patients had overhydaration (the ratio of extracellular water to total body water >0.40). During 23.5 (0–27) months, 12 and 2 patients died. Total 37 patients (34.3%) admitted 2.5 (1–15) times and 23 (1–209) days, respectively. Sarcopenia had no difference of survival and hospitalization. Obesity was not associated with survival, but with hospitalization. Overhydation was associated with survival (hazard ratio [HR] 5.8; 95% confidence interval [CI], 1.54–21.9, *p* < 0.01) and hospitalization (HR 1.69; 95% CI, 1.08–2.66, *p* = 0.022), respectively. However, this difference disappeared after adjusting age, sex, diabetes, weight and albumin.

**Conclusions:** Hydration status may predict survival and outcomes of HD patients rather than fat or muscle mass. Further controlled studies are need to confirm the effect of body composition in HD patients.

**1–12**

**Inter‐rater reliability of the assessment of rectus femoris thickness in older adults using a portable A‐mode ultrasound system**

**Martine J. Sealy** ^1,2^, Willemke Nijholt^1,3^, J.S.M. Hobbelen^1,4^, Cees P. van der Schans^1,3,5^ and Harriet Jager‐Wittenaar^1,2^

^1^ *Research Group Healthy Aging, Allied Health Care and Nursing, Hanze University of Applied Sciences, Groningen, The Netherlands;* ^2^ *Department of Maxillofacial Surgery, University of Groningen, University Medical Center Groningen, Groningen, The Netherlands;* ^3^ *Department of Rehabilitation Medicine, University of Groningen, University Medical Center Groningen, Groningen, The Netherlands;* ^4^ *Department of General Practice and Elderly Care Medicine, University of Groningen, University Medical Center Groningen, Groningen, The Netherlands;* ^5^ *University of Groningen, University Medical Center Groningen, Department of Health Psychology Research, Groningen, The Netherlands*

**Introduction:** Loss of muscle mass is a major problem and is common in elderly. Therefore, the assessment of muscle mass has become an important measure in daily practice. Ultrasound can be used to assess peripheral muscle size, however little is known about inter‐rater reliability of measures by A‐mode ultrasound and how reliability may be influenced by rater experience. In this study we explored agreement between two raters, one experienced and one unexperienced in ultrasound analysis, in assessing rectus femoris thickness, using a portable A‐mode ultrasound.

**Methods:** Rectus femoris thickness was measured by A‐mode ultrasound (BodyMetrix) in community dwelling Dutch older adults. Trained students conducted the ultrasound measurements. Two raters (one experienced radiographer and one dietitian unexperienced in ultrasound) performed the analyses of each image independently of each other. A paired sample T‐test was performed to evaluate systematic differences, and intraclass correlation coefficient (ICC) and Bland Altman plotting were used to assess agreement between the raters. ICC ≥0.90 was considered excellent and *p* < 0.05 significant.

**Results:** 56 older adults were included. Images of 40 participants (median age 63y [range 56–88 y], 50% male) were interpretable and analysed. Mean rectus femoris thickness was 6.01 cm ±1.19 cm for males and 4.98 ± 1.48 cm for females (*p* = 0.021). Agreement between the two raters was excellent on group level (ICC = 0.91 [95%CI: 0.83–0.95]). Means muscle size as assessed by the unexperienced rater was significantly lower than that of the experienced radiographer (−0.13 cm; *p* = 0.040). Limits of agreement range from 0.65 cm to −0.91 cm.

**Conclusions:** In this sample of older adults, agreement between the raters in assessing rectus femoris thickness was excellent on group level and moderate on individual level. Our results indicate on group level analysis of A‐mode ultrasound images can be performed by different raters. Providing more training to unexperienced raters may further minimize measurement errors.

**1–13**

**The comparison of BIA and DXA for estimating lean body mass and percent body fat in ambulatory patients**

**Ryan Hurt** and Manpreet Mundi

*Mayo Clinic, Rochester, USA*

**Introduction**: Despite malnutrition being associated with increased mortality there has been great difficulty in defining what constitutes malnutrition. A decreased lean body mass (LBM) has been associated with increased risk of falls and impaired ability to perform routine activities of daily living. Tools used to diagnosis decreased LBM (sarcopenia) should be easy to use, relatively inexpensive, and safe. Bioelectrical impedance analysis (BIA) has the potential to meet these criteria but reliability across BMI classes is a concern for many providers. The purpose of the current study was to compare LBM and percent body fat measurements using BIA versus dual‐energy X‐ray absorptiometry (iDXA) in participants across BMI categories.

**Methods:** The Mayo IRB approved the current study. A total of 176 healthy non‐pregnant ambulatory participants (18–65 years of age) were recruited equally (*n* = 44) in 4 BMI (kg/m2) categories: 1) 18.5–24.9, 25.0–29.9, 30–34.9, ≥35.0. Participants were fasting overnight and had BIA (InBody 770; InBody Company, South Korea) measurements the next morning and immediately following this iDXA measurements iDXA were performed**.**

**Results:** The measurements (mean ± SD) for LBM with iDXA was 52.8 ± 11.0 and BIA was 53.6 ± 11.0. Delta (InBody‐DXA) was 0.8 ± 2.2 (5% limits of agreement −3.5 to +5.2) and concordance correlation coefficient (CCC) was 0.98 (95% CI 0.97,0.98). The measurements (mean ± SD) for percent body fat with DXA was 37.5 ± 10.6% and BIA was 36.6 ± 11.3%. Delta (InBody‐DXA) was −0.9 ± 2.6 (5% limits of agreement −6.0 to +4.2) and concordance correlation coefficient (CCC) was 0.97 (95% CI 0.96,0.98). The CCC according to the four body mass groups for LBM was between 0.96–0.98 and for percent body fat 0.90–0.94.

**Conclusions:** LBM and percent body fat measured by BIA had good agreement with iDXA across all BMI categories measured in the current study of ambulatory participants.

**1–14**

**Sex differences in the relationship of concurrent change in knee extension peak torque and body composition with change in gait speed**

**Yusuke Osawa**, Nancy Chiles Shaffer, Michelle D. Shardell, Stephanie A. Studenski and Luigi Ferrucci

*Longitudinal Studies Section, Translational Gerontology Branch, National Institute on Aging, National Institutes of Health, Baltimore, Maryland, USA*

**Introduction:** Muscle strength and body composition are known to change over time but the relationship of joint changes in these parameters with change in mobility is not known. We investigated whether changes in muscle strength (Δ peak torque), appendicular lean mass (Δ *ALM*), and whole‐body fat mass (Δ *fat*) jointly relate to changes in gait speed (Δ gait speed).

**Methods:** The analytic sample comprised 575 women and 539 men aged 22–95 years enrolled in the Baltimore Longitudinal Study of Aging. Mean follow‐up time was 3.99 (range, 1–11) years. Measures included isometric knee extension peak torque, DXA‐assessed ALM and fat, and gait speed from the 400 m fast pace walk. Sex‐specific linear mixed models were adjusted for follow‐up time and baseline age, race, height, ALM, fat mass, peak torque, and gait speed. We additionally included second order interaction terms of the key predictive variables (e.g., Δ peak torque‐by‐Δ ALM).

**Results:** In both sexes, independent of change in ALM and fat, larger decline in peak torque was significantly associated with larger decline in gait speed (*p* = 0.01 for male and female). In men, significant associations of Δ peak torque‐by‐Δ ALM or Δ fat‐by‐Δ ALM interactions with simultaneous gait speed decline suggest that men with greater declines in peak torque and ALM or greater declines in ALM and fat mass have steeper gait speed decline. In women, a significant interaction of Δ fat‐by‐Δ ALM suggests that women with fat mass increase combined with lesser decline in ALM have steeper gait speed decline.

**Conclusions:** While changes in strength affect simultaneous change in gait speed in men and women, the influence of body composition differs by sex.

**1–15**

**A body‐fixed‐sensor‐based analysis of stair ascent and sit‐to‐stand to detect age‐related differences in leg‐extensor power**

**Evelien Van Roie** ^1^, Stijn Van Driessche^1^, Bas Huijben^2^, Rob C. van Lummel^2^ and Christophe Delecluse^1^

^1^ *KU Leuven, Department of Movement Sciences, Physical Activity, Sports and Health Research Group, Belgium;* ^2^ *McRoberts BV, The Hague, The Netherlands*

**Introduction:** Human aging is accompanied by a progressive decline in leg‐extensor power (LEP). LEP is typically measured with specialized and expensive equipment, which limits the large‐scale applicability in clinical settings. Previously, sensor‐based trunk kinematics have been used to estimate the vertical power required to elevate the body's center of mass during functional tests, but the link with LEP and age remains to be investigated. Therefore, we investigated whether a body‐fixed sensor‐based analysis of power in stair ascent (SA) and sit‐to‐stand (STS) is positively related to LEP and whether it declines similarly because of age. In addition, the effect of load during SA and STS was investigated.

**Methods:** 96 adults (20–70 years) performed a multi‐joint LEP, a SA and a 5‐repetition STS test. In SA and STS, two conditions were tested: unloaded and loaded (+10% body mass). Data of a wearable motion sensor (DynaPort MT, McRoberts, The Hague, The Netherlands) were used to analyse (sub)‐durations and vertical power.

**Results:** SA and STS power were more related to LEP than duration parameters (i.e. 0.80–0.81 for power and − 0.41 – −0.66 for duration parameters, *p* < 0.05). The annual age‐related percent change was higher in SA power (−1.38%) than in LEP (−0.86%) and STS power (−0.38%) (p < 0.05). Age explained 29% in SA power, as opposed to 14% in LEP and a non‐significant 2% in STS power. The addition of 10% load during SA and STS did not influence the age‐related decline in power nor the relationship with LEP.

**Conclusions:** These results emphasize the potential of body‐fixed sensor‐based analyses of SA power to detect early deterioration in neuromuscular function.

**1–16**

**Interleaved multiparametric multinuclear dynamic NMR imaging and spectroscopy: a non‐invasive setup to further investigate the skeletal muscle functional alterations associated with sarcopenia**

**Pierre G. Carlier** ^1^, Alfredo Lopez Kolkovsky^1^, Harmen Reyngoudt^1^, Benjamin Marty^1^, Eric Giacomini^1^, Damien Bachasson^2^ and Jean‐Yves Hogrel^2^

^1^ *Institute of Myology, Neuromuscular Investigation Center, AIM & CEA NMR Laboratory, Paris, France;* ^2^ *Institute of Myology, Neuromuscular Investigation Center, Neuromuscular Physiology Laboratory, Paris, France*

**Introduction:** There is a need to better understand and further investigate the complex interactions between alterations of perfusion, mitochondrial oxidative phosphorylation, muscle structure and mass and loss of strength and ultimately of autonomy in advanced age. Nuclear magnetic resonance (NMR) can assess non‐invasively skeletal muscle anatomy (mass, structure and composition), physiology (perfusion, capillary and intramyocyte oxygenation) and biochemistry (mitochondrial ATP production, lactate production, pH regulation). However, in a standard setting, each of these variables is acquired separately, during dedicated acquisitions, possibly after modification of coil and system configuration, which considerably reduced the possibilities of studying the interplay between these variables.

**Methods:** Modifications were introduced to a commercially available 3 T NMR scanner in order to collect simultaneously these variables during and/or after an exercise bout. Fully digitally controlled non‐magnetic ergometers were developed in‐house. Off‐the‐shelf dual‐tune 1H/31P coils were used or were developed in collaboration with Rapid Biomedical. A range of interleaved sequences were written allowing simultaneous measures of e.g. perfusion by arterial spin labeling, intramyocytic oxygenation with 1H spectroscopy of deoxymyoglobin, ATP production from the phosphocreatine rephosphorylation rate determined by 31P spectroscopy, etc**.**

**Results:** Proof‐of‐concept has so far been demonstrated on healthy volunteers. The following variables were successfully measured during plantar flexion exercise and recovery: phosphocreatine and inorganic phosphate dynamics, mitochondrial oxidative capacity, pH, muscle reoxygenation rate, oxygen extraction and tissue perfusion. The results were in agreement with literature data, with, for example, a peak perfusion response after exercise of 57.2 ± 3.9 mL.min‐1.100 g‐1, in the gastrocnemius whereas the soleus presented no activation.

**Conclusions:** This pilot work demonstrates the feasibility of using interleaved multiparametric multinuclear NMR in the skeletal muscle at a temporal resolution of 2 seconds during a dynamic paradigm in a clinical setting. Such an approach can be used to study the interactions between structural, hemodynamic and biochemical alterations in sarcopenic patients.

**1–17**

**6 weeks of strength endurance training attenuates senescence‐prone T‐cells in peripheral blood in community‐dwelling older women**

**Ivan Bautmans** ^1,2,3^, Hung Cao Dinh^1,2^, Oscar Okwudiri Onyema^1,2^, Ingo Beyer^1,2,3^, Keliane Liberman^1,2^, Liza De Dobbeleer^1,2^, Wim Renmans^4^, Sam Vander Meeren^4^, Kristin Jochmans^4^, Andreas Delaere^1,2^, Veerle Knoop^1,2^ and Rose Njemini^1,2^

^1^ *Frailty in Aging Research Group, Vrije Universiteit Brussel, Brussels, Belgium;* ^2^ *Gerontology Department, Vrije Universiteit Brussel, Brussels, Belgium;* ^3^ *Department of Geriatric Medicine, Universitair Ziekenhuis Brussel, Brussels, Belgium;* ^4^ *Laboratory of Haematology, Universitair Ziekenhuis Brussel, Brussels, Belgium*

**Introduction:** Aging is characterized by a progressive decline in immune function known as immunosenescence (IS). While the causes of IS are likely to be multi‐factorial, an age‐associated accumulation of senescent T‐cells and decreased naive T‐cell repertoire are key contributors to the phenomenon. There is growing evidence that IS and its dysregulation may play a role in the development of sarcopenia. Therefore, targeting senescent cells is thought to be a promising way to prevent or alleviate age‐related sarcopenia. Although exercise is recognized as a safe countermeasure for IS, few studies have explored its long‐term effect on IS. Moreover, the optimum training condition required to obtain beneficial results in older subjects is lacking. Therefore, we investigated the impact of different training modalities on makers of IS in elderly women.

**Participants and Methods:** One hundred older women (aged 65 years and over) were randomized to 2–3 times/weekly training for 6 weeks at either intensive strength training (3x10 repetitions at 80% one‐repetition maximum (1RM), *n* = 31), strength endurance training (SET, 2x30 repetitions at 40% 1RM, *n* = 33), or control (flexibility training, FT, *n* = 36). The T‐cell percentage and absolute blood counts were determined before and after 6 weeks (24 h–48 h after the last training session) using flow cytometry and haematology analyser.

**Results:** We report for the first time that 6 weeks of SET significantly decreased the basal percentage and absolute blood counts of senescence‐prone T‐cells, which was positively related to the number of training sessions performed. On the other hand, no significant change was observed following IST or FT.

**Conclusions:** The results indicate that SET protocols with many repetitions ‐ at a sufficiently high external resistance ‐ might favour the reduction of senescence‐prone T‐cells in older women. Whether SET might represent a potential novel strategy to delay the onset and impede the progression of IS requires additional investigation.

**1–18**

**Myosteatosis is associated with poor physical fitness in patients undergoing hepatopancreatobiliary surgery**

Malcolm A. West^1^, **David P.J. van Dijk** ^2,3^, Denny Z.H. Levett^4^ and Steven W.M. Olde Damink^2,5^

^1^ *Academic Unit of Cancer Sciences, Faculty of Medicine, Department of Surgery, University of Southampton, Southampton, United Kingdom;* ^2^ *Department of Surgery, Maastricht University Medical Centre and Department of Surgery, NUTRIM School of Nutrition and Translational Research in Metabolism, Maastricht University, Maastricht, the Netherlands;* ^3^ *Department of General Surgery, Zuyderland Medical Centre, Heerlen, the Netherlands;* ^4^ *Anaesthesia and Critical Care Research Group, Southampton Biomedical Research Centre, University Hospital Southampton NHS Foundation Trust and University of Southampton, Southampton, United Kingdom;* ^5^ *Department of General, Visceral and Transplantation Surgery, RWTH University Hospital Aachen, Aachen, Germany*

**Background:** Body composition assessment using a single computed tomography (CT) slide at L3‐level and cardiopulmonary exercise testing (CPET) are widely used for perioperative risk assessment. Both sarcopenia (i.e. low skeletal muscle mass), myosteatosis (i.e. low skeletal muscle radiation attenuation) and poor objectively measured fitness have been associated with poor post‐operative outcomes and survival in various cancer types. However, the association of CT body composition and physical fitness have not been explored. In this study, we will assess the association of CT body composition with selected CPET variables in patients undergoing hepatopancreatobiliary surgery.

**Methods:** A pragmatic prospective cohort of 123 patients undergoing either pancreatic or liver surgery were recruited. All patients underwent preoperative CPET. Preoperative CT‐scans were analysed using a single CT‐slice at L3‐level to assess skeletal muscle mass and adipose tissue mass as well as muscle radiation attenuation (average Hounsfield units of skeletal muscle tissue at L3‐level). Multivariate linear regression was used to assess the association between CT‐body composition variables and CPET variables. Main outcomes were peak oxygen uptake (VO_2_ at Peak) and oxygen uptake at anaerobic threshold (VO_2_ at AT).

**Results:** One‐hundred‐and‐thirteen patients had good quality abdominal CT‐scans available and were included. Of the CT‐body composition variables, skeletal muscle radiation attenuation had the strongest correlation with VO_2_ at Peak (*r* = 0.57, *p* < 0.001) and VO_2_ at AT (*r* = 0.41, p < 0.001). In multivariate analysis, only skeletal muscle radiation attenuation was significantly associated with VO_2_ at Peak (B = 0.25, 95%‐CI 0.15–0.34, p < 0.001, R^2^=0.42) and VO_2_ at AT (B = 0.13, 95%‐CI 0.05–0.21, p < 0.001, R^2^=0.20). Skeletal muscle mass showed no significant associations.

**Conclusions:** There is a positive association between preoperative CT skeletal muscle radiation attenuation and preoperative fitness (VO_2_ at AT and at Peak). This study demonstrates that myosteatosis and not sarcopenia is associated with poor fitness. Combining both variables might provide additive accuracy during perioperative risk assessment.

**1–19**

**Cutt‐off scores for grip strength, based on a young and healthy reference group**

**David Beckwée** ^1,2,3,4,5^, Ingo Beyer^2,3^, Sandra De Breucker^6^ and Ivan Bautmans^2,3,4^ and Belgian Society of Gerontology and Geriatrics, Guideline Development Group on Sarcopenia

^1^ *Rehabilitation Sciences Research department, Vrije Universiteit Brussels, Belgium;* ^2^ *Gerontology;* ^3^ *Frailty in Aging (FRIA) research department, Vrije Universiteit Brussels, Belgium;* ^4^ *Department of Geriatric Physiotherapy, SOMT University of Physiotherapy, Amersfoort, The Netherlands;* ^5^ *Department of Rehabilitation Sciences and Physiotherapy, Faculty of Medicine and Health Sciences, University of Antwerp, Belgium;* ^6^ *Hôpital Erasme –Université Libre de Bruxelles, Brussels, Belgium*

**Objective(s):** Dynapenia or age‐related loss of muscle strength is an important aspect of sarcopenia. The actual cut‐off scores for muscle weakness that are suggested by the European Working Group on Sarcopenia in Older People (EWGSOP) are based on gait speed. Consequently, these cut‐off scores represent a proxy for physical performance rather than being an independent outcome measure for strength. Moreover, these scores do not take into account the heterogeneity regarding older adult's health status. Therefore, in line with the T‐scores of bone mineral density assessments in osteoporosis, this work aims to present new cut‐off scores for grip strength, based on normative data of young and healthy people, acquired from a systematic review with meta‐analyses.

**Material and Methods:** Pubmed was searched systematically using keywords corresponding to muscle strength and reference values. Studies, reporting reference values for grip strength of young (18–39 years) men or women, were included. Data was extracted and pooled for both sexes (meta‐analyses). Subsequently, the pooled standard deviation was calculated by using the Welch‐Satterthwaite equation for pooled degrees of freedom. Finally, cut‐off scores were calculated based on the pooled mean and standard deviation (T‐values).

**Results:** After screening 910 studies, 15 were included in the meta‐analysis. The pooled mean grip strength for men (*n* = 1755) and women (*n* = 2194) was 49.8 kg (95% Confidence Interval (CI) (48.1, 51.5)) and 30.6 kg (95%CI (29.3, 31.9)) respectively. Hence, grip strength cut‐off scores were calculated for men (T_1_ = 38 kg; T_2_ = 25 kg) and women (T_1_ = 21 kg; T_2_ = 11 kg).

**Conclusions:** The presented cut‐off scores for grip strength enable the interpretation of muscle function regardless of physical performance and the health status of the older individual. Further research should be done to validate these cut‐off scores.

**1–20**

**The influence of short‐term and long‐term detraining on muscle strength and body composition after following a resistance training program in older adults**

**Keliane Liberman** ^1,2^, Bart Moreels^1,2^, Veerle Knoop^1,2^, Liza De Dobbeleer^1,2^, Axelle Costenoble^1,2^, Rose Njemini^1,2^, Ingo Beyer^1,2,3^ and Ivan Bautmans^1,2,3^

^1^ *Frailty in Aging Research Univ;* ^2^ *Gerontology Department, Vrije Universiteit Brussels, Belgium;* ^3^ *Geriatrics Department, Universitair Ziekenhuis Brussels, Belgium*

**Background:** After stopping an exercise intervention, loss of muscle gain occurs. Training load might influence the rate of detraining in older persons.

**Purpose:** To investigate the effects of short‐term (6 months) and long‐term (9 months) detraining following resistance training on muscle strength (MS) and appendicular muscle‐fat ratio (AMFR) in older adults.

**Methods:** Community‐dwelling older adults (71 ± 5 years) participating in the Senior's Project Intensive Training (SPRINT) who were randomized into 3–6 months' of 3x/week intensive strength training (IST: 75–80% of 1RM; *n* = 31) or strength endurance training (SET: 40–50% of 1RM; *n* = 32), and of whom data at 1 year follow‐up were available were included in this analysis. Muscle strength (MS), and appendicular muscle fat ratio (AMFR) were assessed through 1RM measurements and bio‐impedance at baseline, 3, 6 and 12 months after baseline.

**Results:** MS increased significantly after 3 and 6 months of resistance training irrespective of training load (IST and SET: all *p* < 0.001). After short‐term detraining significant decreases were observed in SET and IST (both p < 0,05). After additional (long‐term) detraining, no significant supplementary changes were found for MS. Compared to baseline, MS in IST and SET remained significantly higher despite detraining of 3 or 6 months (both p < 0,001). No significant differences were found for AMFR.

**Conclusions:** MS increases significantly after 3 and 6 months of training. However, detraining occurs very rapidly. Compared to baseline, MS remains higher in IST and SET suggesting that both interventions are efficient to counter sarcopenia on the longer term.

**1–21**

**The effect of a six‐month intensive strength training and strength‐endurance training on muscle strength and body composition in older adults: A randomized controlled trial**

**Keliane Liberman** ^1,2^, Lise Demesmaeker^2^, Veerle Knoop^1,2^, Liza De Dobbeleer^1,2^, Axelle Costenoble^1,2^, Rose Njemini^1,2^, Ingo Beyer^1,2,3^ and Ivan Bautmans^1,2,3^

^1^ *Frailty in Aging Research Unit;* ^2^ *Gerontology Department, Vrije Universiteit Brussels, Belgium;* ^3^ *Geriatrics Department, Universitair Ziekenhuis Brussels, Belgium*

**Background:** Aging is associated with sarcopenia, dynapenia and changes in body composition. Previous research has demonstrated that resistance training is currently the most effective, non‐pharmacological method to increase muscle mass and strength. However, there is no consensus yet regarding most favourable intervention in older adults.

**Objective:** To evaluate the effect of intensive strength training (IST) and strength endurance training (SET) on muscle strength and body composition in healthy older adults. Furthermore, our aim is to analyse the differences between different training durations on these outcomes.

**Methods:** 145 community‐dwelling older adults aged ≥65 years participating in the Senior's Project Intensive Training (SPRINT) were randomized into 3 or 6 months' exercise 3x/week at either: IST (70–80% of 1RM, *n* = 44), SET (40–50% 1RM, *n* = 52) or flexibility training (FT) group (*n* = 49). Muscle strength was determined by 1RM and body composition was assessed by bioelectrical impedance analysis. Outcomes were assessed at baseline and after three and six months training.

**Results:** Muscle strength increased significantly in all interventions after 3 and 6 months. However, only in the IST group, significant increases were observed between 3 and 6 months (*P* = 0.009). There was a significant difference in increase in muscle strength between the FT and the IST after three (*p* = 0,022) and six months training (p = 0,007). No significant changes in body composition were observed.

**Conclusions:** Although all interventions led to an increase in muscle strength compared to baseline values, only the IST‐group showed significant increases in muscle strength between third and sixth month of training or compared to FT.

**1–22**

**Changes overtime in physical activity level among hemodialysis patients and the association with body composition and the myokine BAIBA**

**Alessio Molfino** ^1^, Maria Ida Amabile^1^, Thomas Ammann^2^, Luana Lionetto^3^, Alessandra Spagnoli^4^, Silvia Lai^1^, Maria Grazia Chiappini^2^ and Maurizio Muscaritoli^1^

^1^ *Department of Clinical Medicine, Sapienza University of Rome, Rome, Italy;* ^2^ *Hemodialysis Unit, Fatebenefratelli “Isola Tiberina” Hospital, Rome, Italy;* ^3^ *Personalized Medicine Unit, Istituto Dermopatico dell'Immacolata‐IRCCS, Rome, Italy;* ^4^ *Department of Public Health and Infectious Diseases, Sapienza University of Rome, Rome, Italy*

**Introduction:** Low physical activity is particularly prevalent among hemodialysis (HD) patients and few studies analysed the impact of physical activity variations in this population. We aimed to evaluate the longitudinal changes in physical activity and its barriers in a cohort of HD patients and the association between the patterns of physical activity changes, body composition, and beta‐aminoisobutyric acid (BAIBA), as circulating myokine.

**Methods:** Chronic HD patients were enrolled in a 24‐month follow‐up. The presence and changes of physical inactivity and its barriers were investigated by validated questionnaires, body composition was assessed by body impedance analysis, and muscle strength by handgrip dynamometer. Plasma BAIBA levels were measured by liquid spectrometry. Parametric and non‐parametric tests were performed, as appropriate.

**Results:** Forty‐nine patients were studied at baseline (51% were inactive and 88% reported barriers to physical activity); 39 patients completed the first‐year follow‐up (51% were inactive and 90% reported the presence of barriers); 29 patients completed the second‐year follow‐up (63% resulted inactive and 96% reported the presence of barriers). The barrier “reduced walking ability” was more frequent in inactive patients with respect to active at month 12 (*P* = 0.003) and at month 24 (*P* = 0.05). At 12‐month follow‐up, active patients had higher ICW (%) (*P* = 0.001) and cellular mass (%) (*P* < 0.001) with respect to inactive patients. Similarly, at month 24 active patients showed, with respect to inactive, higher ICW (*P* = 0.012) and cellular mass (*P* = 0.002). The all cohort showed, with respect to baseline, a significant reduction in ICW at month 12 (*P* = 0.011) and at month 24 (*P* = 0.014). At the end of the second year, significant correlation was seen between muscle strength and ICW (*r* = 0.51, *P* = 0.005) and BAIBA levels were higher among active patients with respect to inactive (*P* = 0.043). When considering BAIBA normalized per BMI, we found it significantly lower with respect to baseline (*P* = 0.004), as well as after normalizing per ICW (P = 0.001), as marker of muscle mass.

**Conclusions:** A high prevalence of physical inactivity persisted during a 24‐month follow‐up, associated with a decline in marker of muscularity and with reduced plasma BAIBA levels. Additional clinical data are needed to clarify the role of BAIBA and body composition changes in HD.

**1–23**

**Prolonged exercise training improves acute type II skeletal muscle fibre stem cell response in healthy older men**

**Tim Snijders** ^1,2^, Joshua Nederveen P^1^, Kirsten E. Bell^1^, Sean W. Lau^1^, Nicole Mazara^1^, Dinesh A. Kumbhare^3^, Stuart M. Phillips^1^ and Gianni Parise^1^

^1^ *McMaster University, Canada;* ^2^ *Maastricht University, The Netherlands;* ^3^ *University of Toronto, Canada*

**Introduction:** The loss of skeletal muscle mass with age is primarily due to type II muscle fibre atrophy which is accompanied by a decline in muscle stem cell (mSC) number and function. Exercise training restores mSC quantity in older adults; however, whether exercise training can restore the impaired mSC response to exercise remains unknown. The present study assessed the acute mSC response to a single bout of resistance exercise before and after 12 weeks of exercise training in older men.

**Methods:** Fourteen older men (72 ± 1 yr) participated in a 12‐week exercise training program. Before and after the combined exercise training program, muscle biopsies were taken before and 24 h and 48 h after a resistance exercise session. Muscle SC content and activation status were evaluated by immunohistochemistry and mRNA expression of the myogenic regulatory factors (MyoD,Myogenin) by RT‐PCR.

**Results:** Whereas no changes were observed in type II muscle fibres, type I muscle fibre mSC content increased significantly at 24 h and 48 h after the single exercise session before the exercise training program (*P* < 0.01). In response to the exercise training program, resting type I (from:0.092 ± 0.007 to 0.114 ± 0.011 mSC per fibre) and type II (from:0.061 ± 0.006 to 0.079 ± 0.007 mSC per fibre) muscle fibre mSC content increased significantly (*P* < 0.05). More importantly, following the exercise training program, both type I and type II muscle fibre mSC content increased significantly at 24 h and 48 h after the resistance exercise session (P < 0.05). The greater acute increase in type II muscle fibre mSC content at 24 h post‐exercise recovery after training was correlated with an increase in type II muscle fibre capillarization (*r* = 0.671,*P* = 0.012).

**Conclusions:** We show, for the first time, that mSC function can be improved by exercise training in older men. This improvement in muscle mSC function shares variance with muscle fibre capillarization suggesting a mechanistic link between the two processes.

**1–24**

**Intradialytic exercise for hemodialysis patients**

**Soo Jeong Choi** ^1^, Min Sung Lee^1^, Seok Hui Kang^2^, Su‐Hyun Kim^3^, Sujung Park^1^, Byung Chul You^1^, Moo Yong Park^1^, Jin Kuk Kim^1^ and Seung Duk Hwang^1^

^1^ *Division of Nephrology, Department of Internal Medicine, Soonchunhyang University Bucheon Hospital;* ^2^ *Division of Nephrology, Department of Internal Medicine, Yeungnam University Hospital, Daegu, Republic of Korea;* ^3^ *Division of Nephrology, Department of Internal Medicine, Chung‐Ang university Hospital, Seoul, Korea*

**Background:** Patients with end stage renal disease (ESRD) had declines in physical function and low level of physical activity. Previous studies have concluded that exercise training is beneficial to patients on hemodialysis (HD). Results, however, have shown that differences in the type, intensity, and frequency of physical exercise lead to variability in its effects on physical functional performance and nutrition. Our aim was to evaluate the effects of aerobic exercise on physical functional performance and nutrition during HD.

**Methods:** Using a pretest‐posttest control design, we enrolled trice weekly HD patients and assigned them to an exercise group that completed a 12 week exercise program using intradialytic cycling or a control group that did no exercise.

**Results:** Total 34 HD patients (55.6 ± 1.9 years old, 17 males) were enrolled. At baseline, Korean instrumental activities of daily living score (KIADL) and daily walking steps were 32.4 (29–33) and 3,367 (130–14,157)/days. The results of 6 minutes walking and sit‐to‐stand (STS) test were 429.4 (306–608) m and 10.5 (6–15)/30 sec, respectively. During 6 months, 2 patients underwent kidney transplantation. Other 3 patients were unable to exercise due to wheel chair using. Only 6 patients completed 12 week intradialytic cycling program. Then, 18 patients followed up physical activities tests at 6 months. The results of 6 minutes walking and STS test were 427.9 (270–648) m and 10.3 (7–14)/30 sec, respectively. Among them, the change of 6 minutes walking test of exercise group was increased compared with control (61 m vs −41 m, *p* = 0.044), while the change of STS test was not different (0.7 vs −0.8, *p* = 0.253). There is no difference of nutritional markers between exercise group and control.

**Conclusions:** Exercise may play a critical role in physical functional performance. But, the effect of exercise need more long term study.

**1–25**

**The effect of intradialytic exercise on physical performance and echocardiographic findings in maintenance hemodialysis patients**

**Jun Chul Kim** ^1^, Su‐Hyun Kim^2^, Seok Hui Kang^3^ and Won Suk An^4^

^1^ *Department of Internal Medicine, CHA Gumi Medical Center, CHA University School of Medicine, Gumi, South Korea;* ^2^ *Department of Internal Medicine, Chung‐Ang university Hospital, Seoul, South Korea;* ^3^ *Division of Nephrology, Department of Internal Medicine, Yeungnam University Hospital, Daegu, South Korea;* ^4^ *Department of Internal Medicine, Dong‐A University, Busan, South Korea*

**Introduction:** Physical performance (PP) is impaired in maintenance hemodialysis (MHD) patients, which leads to a high risk of mortality and hospitalizations. We investigated the effect of 12‐week intradialytic exercise (IDE, 3 times/week) on PP and echocardiographic findings in MHD patients.

**Methods:** We randomly assigned MHD patients on dialysis≥6 months, to 4 exercise groups: aerobic (AE, *n* = 11), resistance (RE, *n* = 10), combination (CE, *n* = 12), and control group (CG, *n* = 13). A stationary bike was used for AE at moderate intensity and a TheraBand®/theraball® for RE at high intensity. The CG received only warm‐up stretching. At baseline and 12‐week, a sit‐to‐stand for 30 seconds test (STS30), a 6‐minute walk test (6‐MWT), and echocardiography were performed.

**Results:** We observed significant increases in STS30 (times/30 s) and 6‐MWT (meters) in the AE (18.7 ± 5.4 vs 16.5 ± 4.8 and 459 ± 122 vs 434 ± 111), RE (24.6 ± 4.9 vs 21.0 ± 5.0 and 530 ± 106 vs 510 ± 102), and CE (24.8 ± 10.7 vs 21.6 ± 9.6 and 514 ± 165 vs 492 ± 167) at 12 weeks compared with baseline, while no improvement in the CG. When comparing between‐group changes in STS30 and 6‐MWT, there were significant increases in the AE (2.3 ± 2.2 vs −0.5 ± 2.2 and 25 ± 29 vs −26 ± 41), RE (3.6 ± 2.7 vs −0.5 ± 2.2 and 20 ± 21 vs −26 ± 41), and CE (3.3 ± 3.1 vs −0.5 ± 2.2 and 22 ± 12 vs −26 ± 41) groups compared with the CG. In the echocardiographic analysis, all exercise groups (AE, RE, and CE) showed no significant effect on left ventricular ejection fraction (%) (2.5 ± 5.2, −0.7 ± 5.3, and − 0.3 ± 5.4 vs 0.3 ± 3.9, respectively), left ventricular mass index (g/m^2^) (−1.5 ± 16.7, 0.8 ± 16.3, and − 0.5 ± 32.0 vs −11.7 ± 30.1, respectively), and myocardial performance index (0.09 ± 0.19, −0.02 ± 0.18, and 0.12 ± 0.21 vs 0.08 ± 0.18, respectively) compared with the CG.

**Conclusions:** Although IDE does not affect the echocardiographic parameters measured, it appears to be clinically beneficial in improving PP. It may suggest that PP was improved by muscular or neurological rather than cardiac function in this study.

**1–26**

**Radiographic and histopathologic analysis of bone regeneration by early osteogenic induced adipose‐derived stem cells in improve method on biodegradable scaffold**

**Kyung‐Ku Kang** ^1,2^, Se‐il Park^3^, Sunray Lee^4^, Hyun‐Sook Park^4^, Myung‐Jin Chung^1,2^, SunYoung Park^1,2^, Soon‐Seok Park^1,2^, Hong‐In Shin^5^ and Kyu‐Shik Jeong^1,2^

^1^ *Department of Veterinary Pathology, College of Veterinary Medicine, Kyungpook National University, Daegu, Republic of Korea;* ^2^ *Stem Cell Therapeutic Research Institute, Kyungpook National University, Daegu, Republic of Korea;* ^3^ *Cardiovascular Product Evaluation Center, Yonsei University College of Medicine, Seoul, Republic of Korea;* ^4^ *Cell Engineering for Origin Research Center, Seoul, Republic of Korea;* ^5^ *Department of Oral Pathology and Regenerative Medicine, School of Dentistry, Kyungpook National University, Daegu, Republic of Korea*

**Background:** The adipose‐derived mesenchymal stem cells of canine animals has a slightly different character from the MSc of human origin such as the difference in CDs marker expression and differentiation potential.

**Methods:** In this study, we used a medium supplemented with antioxidants such as GSH and low in glucose content, canine adipose tissue derived MSCs. The cells prepared for bone cell therapy were allogeneic cells, which were short‐time‐induced cells of early osteogenic differentiation and were more targeted for therapeutic purposes than those of undifferentiated ADMSCs transplantation. We obtained about adipose tissue for canine adipose tissue derived mesenchymal stem cells (cADMSCs) isolation. To transplant cells in to the long bone of canine, canine were divided into 3 groups: Control, Cell + Scaffold (C/S), Cell + Scaffold + Immunosupressant (C/S/I, Cyclosporin, 25 mg/kg/day). Canine femoral segmental defeat were performed and after 12 weeks, animal were sacrificed and femur were isolated. The osteogenic differentiated cells were prepared by encapsulation in hydrogel.

**Results:** The cADMSC expressed CDs markers similar to typical mesenchymal stem cells such as CD44, CD73 and CD90 at passage 3. On the X‐ray examination, bone regeneration was confirmed at 2 weeks after surgery, and the response was clearly observed at 4 weeks. In particular, C/S group showed faster regeneration than other groups. As a result of microCT analysis, the degree of bone formation in defect was C/S group > C/S/I group > Control group in CT image. In case of BMD, defect femur was lower than control femur but it was approximated in C/S group.

**Conclusions:** In conclusion, the results of the present study demonstrate that transplantation of early osteogenic induced cADMSCs after culturing in CEFOgro™ cADMSC show improve new bone formation efficiency in canine segmental bone defects.

**1–27**

**Prevalence of sarcopenia and malnutrition in community‐dwelling older adults in Ireland**

**Caoileann H. Murphy** ^1^, Aoibheann M. McMorrow^1^, Ellen M. Flanagan, Helen Cummins^2^, Sinead N. McCarthy^2^, Maureen J. McGowan^3^, Sheena Rafferty^4^, Brendan Egan^5^, Giuseppe De Vito^1^ and Clare A. Corish^1^ and Helen M. Roche^1^

^1^ *University College Dublin, Ireland;* ^2^ *Teagasc Food Research Centre, Dublin, Ireland;* ^3^ *Health Service Executive Community Health Organization 6, Wicklow, Ireland;* ^4^ *Health Service Executive Community Health Organization 9, Dublin, Ireland;* ^5^ *Dublin City University, Dublin, Ireland*

**Introduction:** Sarcopenia, the age‐related loss of skeletal muscle mass and function, is an independent risk factor for numerous adverse health outcomes. Older adults are at increased risk for chronic malnutrition, a condition that may accelerate the loss of muscle mass and physical function. The aim of this study was to identify the prevalence of sarcopenia and malnutrition among community‐dwelling older adults in Ireland for the first time.

**Methods:** In a cross‐sectional analysis, 498 community‐dwelling adults (age 78.5 ± 8.0 y, body mass index (BMI) 27.6 ± 5.1 kg/m^2^) were assessed. Skeletal muscle mass was measured using bioelectrical impedance analysis, muscle strength via handgrip dynamometry and physical performance via the Short Physical Performance Battery. Sarcopenia was defined according to the European Working Group on Sarcopenia in Older People (EWGSOP) criteria. Malnutrition risk was assessed using the Malnutrition Universal Screening Tool (MUST).

**Results:** The prevalence of sarcopenia was 9.0%. Of those that did not meet the EWGSOP definition for sarcopenia, 2.9% were pre‐sarcopenic (low muscle mass without a decrement in strength or physical performance) and 61.7% had low strength and/or physical performance in the absence of low muscle mass. Using MUST, 10.0% of participants were classified as “at risk” of malnutrition. Among participants with sarcopenia, 10% were at medium risk and 29% were at high risk of malnutrition. BMI within the underweight and obese categories were associated with higher risk of sarcopenia (*p* < 0.05).

**Conclusions:** Sarcopenia is prevalent among community‐dwelling older adults living in Ireland and, even among those who did not meet the EWSGOP definition for sarcopenia or pre‐sarcopenia, decrements in strength and physical function are common. Our data indicate that underweight and obesity are risk factors for sarcopenia, however the MUST tool has low predictive value for the identification of sarcopenia.

**1–28**

**NT‐proBNP levels predict lower long‐term physical function and muscle strength in sepsis survivors**

**Sai Gogineni**, Quran Wu, Stephen Anton, Scott Brakenridge, Christiaan Leeuwenburgh, Lyle Moldawer, Frederick Moore and Robert Mankowski

*University of Florida, Gainesville, Florida, USA*

**Introduction:** Sepsis is an exaggerated response to infection with high mortality. Sepsis survivors often fail to recover and experience long‐term impairments in physical, cardiovascular and cognitive function. Even among those who initially recover, physical function declines over time. Thus, there is a need to identify biomarkers that can predict long‐term worsening of physical function to be able to act early and prevent mobility loss. N‐terminal pro b‐type natriuretic peptide (NT‐proBNP) is a classic blood biomarker associated with cardiovascular events and all‐cause mortality. We tested whether higher NT‐proBNP levels in acute phase of sepsis would be associated with lower physical function and muscle strength at 6 months after sepsis onset.

**Methods:** We analysed 65 sepsis patients enrolled in the University of Florida Sepsis and Critical Illness Research Center who consented to participate in the 12 month follow‐up study. In this retrospective analysis we tested whether NT‐proBNP levels at day 1 after sepsis onset are associated with short physical performance battery (SPPB) test score (scale 0–12 – higher score corresponds with better function) and hand grip strength results (kilograms) from a 6 month follow‐up ambulatory visit. We used a multi‐variate generalized linear model to test an association between NT‐proBNP levels, physical function and grip strength scores. Statistical significance was set at *p* < 0.05.

**Results:** After adjusting for covariates (age, sex and disease severity) higher NT‐proBNP levels at day 1 predicted lower SPPB score (*p* = 0.01) and hand grip strength (*p* = 0.008) among sepsis survivors (*n* = 65, aged 18–80 years old) at 6 month follow‐up.

**Conclusions:** Levels of NT‐proBNP during the acute phase of sepsis may be a useful indicator of impairments in long‐term physical function and muscle strength.

**1–29**

**Older men with dynapenic obesity have more fragility fractures – the prospective STRAMBO study**

**Pawel Szulc** and Roland Chapurlat

*INSERM UMR1033, Unviersity of Lyon, Hospices Civils de Lyon, Lyon, France*

**Introduction:** Dynapenic obesity is associated with disability and mortality. Data on its link with fractures are scarce.

**Methods:** We studied the relation between dynapenic obesity and fracture risk prospectively assessed in 814 men aged 60–88. Bone mineral density (BMD) and body composition were assessed by DXA (Hologic Discovery A). Dynapenic obesity was defined as the coexistence of fat fraction >24% and grip strength <66 kPa (Vigorimeter Martin). The reference group had both variables within the normal range. The intermediate group had one of the 2 criteria. Self‐reported incident peripheral fractures were checked. Incident vertebral fractures were assessed on DXA scans. We used Cox model adjusted for age, hip BMD, prior falls and fractures, and ischemic heart disease. Fracture prediction improvement was assessed by comparison of the areas under the curve (AUC) using Harrell's test.

**Results:** During the 8‐year prospective follow‐up, 98 men had incident fractures. Age‐standardized fracture incidence in the reference group, the intermediate and the dynapenic obesity group was 4, 17 and 33/1000 person‐years, respectively. In comparison with the reference group, fragility fracture risk was higher in men with dynapenic obesity (HR = 2.64, 95%CI: 1.42–4.90, *p* = 0.002), but not in the intermediate group (HR = 1.52, 95%CI: 0.84–2.75). Similar patterns were found for fracture subgroups (vertebral, major osteoporotic, peripheral), e.g. peripheral fracture, HR = 3.18, 95%CI: 1.43–7.08, *p* < 0.005. Dynapenic obesity was associated with higher fracture risk in the 565 osteopenic men (T‐score < −1.0 at lumbar spine, hip or femoral neck): HR = 3.21, 95%CI: 1.65–6.25, *p* < 0.001, but not in men with normal BMD. In the osteopenic group, dynapenic obesity improved fracture prediction vs. traditional model (AUC = 0.724 vs. 0.672, *p* < 0.01). Dynapenic obesity was also associated with fracture subtypes, e.g. peripheral fracture, AUC = 0.698 vs. 0.626, p < 0.05.

**Conclusions:** Dynapenic obesity is associated with higher risk of fracture in older men. Dynapenic obesity significantly improved fracture prediction in older men with osteopenia.

**1–30**

**Longitudinal muscle and myocellular changes in community‐dwelling men over two decades of successful aging ‐ The ULSAM cohort revisited**

**Elisabeth Skoglund** ^1,2,3^, Max Grönholdt‐Klein^4" noteRef="jcsm12365-subcmp-0027-note-0001^, Eric Rullman^1" noteRef="jcsm12365-subcmp-0027-note-0001^, Lars Eric Thornell^3^, Anna Strömberg^1^, Anu Hedman^5^, Tommy Cederholm^2^, Brun Ulfhake^4" noteRef="jcsm12365-subcmp-0027-note-0002^ and Thomas Gustafsson^1" noteRef="jcsm12365-subcmp-0027-note-0002^

^1^ *Department of Laboratory Medicine, Division of Clinical Physiology, Karolinska Institutet, and Unit of Clinical Physiology, Karolinska University Hospital, Stockholm, Sweden;* ^2^ *Department of Public Health and Caring Sciences, Clinical Nutrition and Metabolism, Uppsala University, Uppsala, Sweden;* ^3^ *Department of Integrative Medical Biology, Umeå University, Umeå, Sweden;* ^4^ *Department of Neuroscience, Karolinska Institutet, Stockholm, Sweden;* ^5^ *Heart Centre East‐Tallinn Central Hospital, Tallinn, Estonia*

^†^Equally contributed,

^‡^Joint senior authors.

**Introduction:** Activation of factors involved in remodelling in aged skeletal muscle has been suggested to be both beneficial to prevent muscle loss as well as to be the cause of the process. This study reports on longitudinal muscular changes over 18 years of advanced aging.

**Methods:** A large cohort from the population‐based Uppsala Longitudinal Study of Adult Men (ULSAM) reaching >88 years of age (survivors) was investigated at age 70, 82 and 88–90. Skeletal muscle morphology and gene expression was analysed longitudinally at 70 and 88–90 years of age in a sub‐group of the survivors (*n* = 28). Gene expression data in survivors was compared to a sub‐group (*n* = 24) of participants reaching <82 years of age (non‐survivors) who had provided a muscle biopsy at 70 years of age and to a young healthy reference group (*n* = 8; 25–35 years old).

**Results:** Body composition and muscle mass were remarkably stable over time. Histology revealed only minor changes between ages 70 to 88–90, with a small increase in type I fibre frequency. At age 70, survivors had greater transcript levels of Myogenin, MyoD, embryonic myosin (eMyHC), NCAM, Smad 2 and E3 ligases Trim 32 and MuRF1 in comparison to the young reference group. Transcript levels of eMyHC, Smad2, and MuRF1 were lower in non‐survivors compared to survivors at age 70. Longitudinal changes in transcript abundance in survivors were evident for Myogenin, MyoD, eMyHC, NCAM and Smad2 (lower at age 88–90), and for beta‐Catenin (higher at age 88–90). Subjects with lower physical function at 88 years of age had lower expression of Myogenin, Smad2, and TRIM32 compared to higher functioning subjects.

**Conclusions:** The gene expression alteration at age 88–90 indicates a dysregulation in remodelling coherent with the earlier observation of a successive loss of re‐innervation capacity and/or plasticity in advanced aged human skeletal muscle.

**1–40**

**Muscle strength and functional capacity in patients with diabetes and chronic heart failure (HF)**

**Mirela Vatic** ^1^, Breno Matheus Wozne Godoy^2,3^, Nicole Ebner^2^, Amir Emami^2^, Goran Loncar^4^, Tania Garfias Macedo^2^, Anja Sandek^2,3^, Stefan D. Anker^5,6,7^, Wolfram Doehner^5,6,8^, Stephan von Haehling^2,3^ and Miroslava Valentova^2,3^

^1^ *Medical University of Goettingen (UMG), Cardiovascular Science program, Goettingen, Germany;* ^2^ *Department of Cardiology and Pneumology, University Medical Center Goettingen, Georg‐August University, Goettingen, Germany;* ^3^ *DZHK (German Centre for Cardiovascular Research), partner site Goettingen, Goettingen, Germany;* ^4^ *Department of Cardiology, Clinical Hospital Zvezdara, Belgrade, Serbia;* ^5^ *Division of Cardiology and Metabolism ‐ Heart Failure, Cachexia & Sarcopenia, Department of Cardiology (CVK), Charité University Medical Center Berlin, Germany;* ^6^ *Berlin‐Brandenburg Center for Regenerative Therapies (BCRT), Charité University Medical Center Berlin, Germany;* ^7^ *DZHK (German Centre for Cardiovascular Research), partner site Berlin, Berlin, Germany;* ^8^ *Center for Stroke Research Berlin (CSB) Charité Universitätsmedizin Berlin, Germany*

**Introduction:** Diabetes mellitus in non‐HF populations lowers exercise tolerance due to impaired cardiac metabolism and endothelial dysfunction. We hypothesized that negative effect of diabetes on functional status does not apply in the setting of HF since similar perturbations ensue from HF itself, and diabetes rather prevents muscle loss.

**Methods:** We analysed 210 HF outpatients (median age 68.4 years, 19% female, median left ventricular ejection fraction 35.0%). Patients were enrolled between February 2010–August 2014 at the Charité Medical School Berlin. Diabetes was confirmed in medical charts. Unknown diabetes was defined as HbA1c > = 6.5%. Functional capacity was assessed by cardiopulmonary exercise test (CPX), hand grip and quadriceps strength test. Body composition was measured by dual‐energy X‐ray absorptiometry.

**Results:** A total of 87 (41.4%) patients had known diabetes. Previously unknown diabetes was found in 15 (7.1%) patients. Baseline characteristics of diabetic (*n* = 102) and non‐diabetic (*n* = 108) patients were similar except for higher prevalence of hypertension (91% vs. 74%) and NYHA class>2 (50% vs. 35%; both *p* ≤ 0.03) in diabetic patients. Functional determinants were similar in both groups except for higher median BMI (30 [IQR:26–34] vs. 27 [25–31]kg/m2; *p* = 0.02) and fat‐free mass (58.5 [49.9–64.1] vs. 53.6 [46.5–58.4] kg; *p* = 0.005) in diabetic subjects. CPX was performed in 87 (85%) diabetic and 90 (83%) non‐diabetic patients. Diabetic vs. non‐diabetic patients had similar median maximal oxygen consumption (1444.3 [1163.2–1751.7] vs. 1479.6 [1159.0–1737.2] ml/min), hand grip strength (36.9 [28.0–47.0] vs. 36.0 [29.0–46.0] kg) and quadriceps strength (37.1 [29.3–46.6] vs. 36.0 [28.5–48.1] kg; all *p* > 0.05).

**Conclusions:** The prevalence of diabetes among HF patients was unexpectedly high (48.5%). Unlike in non‐HF subjects, diabetes was not associated with worse functional capacity in patients with HF. Diabetic patients with HF seem to have more skeletal muscle mass as indicated by higher fat‐free mass.

**1–41**

**Lower body mass index (BMI) is associated with suboptimal medication dosing in patients with heart failure: a possible contributor to the obesity paradox**

**Miroslava Valentova** ^1,2^, Breno Matheus Wozne Godoy^1,2^, Nicole Ebner^1,2^, Mirela Vatic^3^, Amir Emami^1,2^, Ruben Evertz^1,2^, Goran Loncar^4,5^, Tania Garfias Macedo^1^, Wolfram Doehner^6,7,8^, Anja Sandek^1,2^, Stefan D. Anker^6,7,9^ and Stephan von Haehling^1,2^

^1^ *Department of Cardiology and Pneumology, University Medical Center Goettingen, Georg‐August University, Goettingen, Germany;* ^2^ *DZHK (German Centre for Cardiovascular Research), partner site Goettingen, Goettingen, Germany;* ^3^ *Medical University of Goettingen (UMG), Cardiovascular Science program, Goettingen, Germany;* ^4^ *Institute for cardiovascular disease Dedinje, Belgrade, Serbia;* ^5^ *Faculty of medicine, University of Belgrade, Serbia;* ^6^ *Division of Cardiology and Metabolism ‐ Heart Failure, Cachexia & Sarcopenia, Department of Cardiology (CVK), Charité University Medical Center Berlin, Germany;* ^7^ *Berlin‐Brandenburg Center for Regenerative Therapies (BCRT), Charité University Medical Center Berlin, Germany;* ^8^ *Center for Stroke Research Berlin (CSB) Charité Universitätsmedizin Berlin, Germany;* ^9^ *DZHK (German Centre for Cardiovascular Research), partner site Berlin, Berlin, Germany*

**Background:** A survival advantage in overweight and obese patients with heart failure (HF) compared to their normal‐to‐low BMI counterparts has been confirmed in numerous trials. We hypothesize that this advantage arises from higher doses of prognostically relevant HF drugs. Here, we investigated the relationship between BMI and doses of HF medications.

**Methods:** We analysed 180 outpatients with left ventricular ejection fraction ≤40% (median age 68.3 years [interquartile range, IQR:60.9–74.2], female 16.1%, New York Heart Failure [NYHA] class II/III/IV 54.4/45.0/0.6%). Doses of angiotensin‐converting‐enzyme inhibitors (ACE), angiotensin‐II‐receptor blockers (ARB), beta‐blockers and mineralocorticoid‐receptor antagonists (MRA) were extracted from medical records. Target doses were defined according to the current guidelines. Achieved drug doses were calculated as percentages of the recommended target doses. Achieved doses <50% of target dose were considered suboptimal.

**Results:** A total of 170 (94.4%), 171 (95.0%) and 101 (56.1%) patients received ACE/ARB, beta‐blockers and MRA, respectively. Median BMI was 27.5 kg/m^2^ (IQR:24.8–32.4). Patients were divided into three equal groups according to BMI: low (≤25.5 kg/m^2^), medium (25.6‐ < 30.5 kg/m^2^) and high BMI (≥30.5 kg/m^2^). Prescription rates of ACE/ARB (94.9% vs. 95.1% vs. 93.3%), beta‐blockers (94.9% vs. 93.4% vs. 96.7%) and MRA (54.2% vs. 57.4% vs. 56.7%) were similar in all BMI groups (all *p* > 0.7). However, achieved doses of HF drugs differed between the groups. A total of 39.3% patients in low BMI group received <50% of target ACE/ARB dose compared to 31.0% and 16.1% patients in medium and high BMI groups, respectively (*p* = 0.001). Similarly, 46.4% patients in low BMI group received <50% of target beta‐blocker dose compared to 36.8% and 22.4% patients in medium and high BMI groups, respectively (*p* = 0.03). Suboptimal MRA doses were rare in all three BMI groups (3.1% vs. 5.7% vs. 0.0%; p = NS). BMI correlated with ACE/ARB dose (Spearman's rho 0.306; *p* < 0.001).

**Conclusions:** Prescription rates of HF drugs were similar among all BMI groups. However, patients with low BMI received more frequently suboptimal doses of ACE/ARB and beta‐blockers compared to patients with medium and high BMI. Suboptimal doses of HF drugs may contribute to worse outcomes in HF patients with normal‐to‐low BMI.

**1–44**

**Skeletal muscle mass index and phase angle are decreased in subjects with dependence of alcohol and other substances**

Marta Paula Pereira Coelho^1^, Kiara Gonçalves Dias Diniz^1^, Tatiana Bering^1^, Lucas dos Santos Athadeu Ferreira^2^, Diego Alves Vieira^2^, Maria Isabel Toulson Davidson Correia^3^, Gifone Aguiar Rocha^4^, Frederico Duarte Garcia^5^ and **Luciana Diniz Silva** ^1,6^

^1^ *Sciences Applied to Adult Health Care Post‐Graduate Programme;* ^2^ *Medical undergraduate student;* ^3^ *Department of Surgery;* ^4^ *Laboratory of Research in Bacteriology;* ^5^ *Department of Mental Health;* ^6^ *Department of Internal Medicine, Faculdade de Medicina, Universidade Federal de Minas Gerais (UFMG), Belo Horizonte, Brazil*

**Aims:** To assess the prevalence of low skeletal muscle mass index and low phase angle and how it is associated with demographic, clinical, lifestyle and nutritional status in patients with dependence of alcohol and other substances.

**Methods:** This is a prospective cross‐sectional study conducted in the Addiction Outpatients Clinic for 24 months. We included 63 subjects with dependence of alcohol and other substances (mean age, 46.0 ± 13.2 yrs; 65.1% males) and 71 healthy subjects (mean age, 44.0 ± 8.2 yrs; 56.3% males). Bioelectrical impedance analysis assessed body composition. Skeletal muscle mass was converted to a skeletal muscle mass index by dividing height by meters squared (kg/m^2^). The cut‐off points for low skeletal muscle mass index, for women and men, were ≤ 5.60 kg/m^2^ and ≤ 8.60 kg/m^2^, respectively. The 5^th^ percentile was adopted as cut‐off point for low phase angle. Subjective global assessment was used to evaluate malnutrition. All the included subjects underwent a psychiatric evaluation, including the administration of the Mini International Neuropsychiatric Interview.

**Results:** Low skeletal muscle mass index and low phase angle were identified in 11.1% and 44.4% of the substance users, respectively. Low mid‐arm muscle circumference, low mid‐arm muscle area and reduced phase angle were positively correlated with low skeletal muscle mass index. Receiver operating characteristic analysis revealed that the optimal phase angle cut‐off value to detect low skeletal muscle mass index was ≤6.05° in men with substance dependence. Low skeletal muscle mass index was independently associated with heavy alcohol consumption (≥80 g/day/≥5 yrs; OR = 2.33; 95%CI = 1.12–4.84; *P* = 0.02) and sedentary lifestyle (OR = 4.39; 95%CI = 1.29–14.89; *P* = 0.02). Low phase angle was independently associated with heavy alcohol consumption (OR = 3.64; 95% CI = 1.62–8.15; *P* = 0.002) and cocaine/crack use (OR = 3.97; 95%CI = 1.05–15.11; *P* = 0.04).

**Conclusions:** Subjects with alcohol and drug dependence, showed low skeletal muscle mass index and low phase angle. These findings highlight the relevance of evaluating the body composition in substance users.

**1–45**

**Depression as a predictor of sarcopenia in older Chileans. The ALEXANDROS study**

**Cecilia Albala** ^1,2^, Carlos Marquez^1,2^, Lydia Lera^1,2^, Bárbara Angel^1,2^, Rodrigo Saguez^1,2^, Mario Moya^1,2^ and Patricia Arroyo^1,2^

^1^ *INTA, University of Chile;* ^2^ *Clinic Hospital, University of Chile*

**Introduction:** Depression is the most common mental disorder in older people. The isolation, inactivity, change in appetite and loss of energy that characterize depression, impairs functioning in daily life and can lead to sarcopenia, the main cause of physical disability in older people.

**Objective:** to determine the risk of sarcopenia as a consequence of depression in older Chileans.

**Methods:** Follow up of ALEXANDROS cohorts designed to study disability associated with obesity in community‐dwelling people 60y and older living in Santiago/Chile. At baseline, 717 from 1126 participants (68.4% women, mean age 72y ± 6.7) had baseline evaluation of depression and the measurements needed for the diagnosis of sarcopenia using the algorithm from the EWGS validated for Chile. Depression was evaluated with the GDS‐15 (cut‐off point 4/5). Information about deaths was available for the entire sample.

**Results:** At baseline, 20.8% of the sample had depressive symptoms. The frequency of sarcopenia in the total sample was 16.6%, higher in people with depression than in the non‐depressive (23.5 vs. 14.8%;p < 0,02) After excluding people with Sarcopenia (*n* = 119) and deaths (*n* = 7), we were able to follow 349 subjects. After 1793.7 person/years of follow‐up, 51 new cases of sarcopenia were identified, 17 cases in people with baseline depression and 34 in the non‐depressive, yielding an incidence rate of 4.72 per 100 person/years in people with depression and 2.37 per 100 person/years in the non‐depressive, *p* < 0.03. After Cox Regression analysis, adjusted by age, gender, years of education and living alone, the HR for sarcopenia in people with depression was HR = 2.13 (95%CI1.07–4,24) *p* = 0.031.

**Conclusions:** We found more than twice the risk of sarcopenia in people with depression than in the non‐depressive. The high frequency of depression in older Chileans, a condition often overlooked and untreated poses an important risk for sarcopenia that should be considered for its prevention.

**1–46**

**Prevalence of pre‐hospital sarcopenia in elderly patients with acute stroke**

**Nozoe Masafumi** ^1^, Masashi Kanai^2^, Hiroki Kubo^2^, Miho Yamamoto^2^, Shinichi Shimada^3^ and Kyoshi Mase^1^

^1^ *Department of Physical Therapy, Faculty of Nursing and Rehabilitation, Konan Women's University;* ^2^ *Department of Rehabilitation, Itami Kousei Neurosurgical Hospital;* ^3^ *Department of Neurosurgery, Itami Kousei Neurosurgical Hospital, Japan*

**Objective:** To investigate the prevalence of pre‐hospital sarcopenia and to determine its association with the functional outcome in elderly patients with acute stroke.

**Methods:** The study population consisted of 155 Japanese patients aged ≥65 years, who were admitted to our hospital for acute stroke. Pre‐hospital sarcopenia was assessed by using a questionnaire for sarcopenia (SARC‐F). According to their SARC‐F score, patients were divided into a non‐sarcopenia group (SARC‐F score < 4) and pre‐hospital sarcopenia group (SARC‐F score ≥ 4). The study endpoint was defined by the modified Rankin Scale score at discharge from the acute hospital settings (0–3, good outcome; 4–6, poor outcome).

**Results:** The prevalence rate of pre‐hospital sarcopenia was 19.4% (30 patients); these patients showed a significantly poorer functional outcome than the non‐sarcopenia group (53% vs. 18%, p < 0.001). Pre‐hospital sarcopenia was found to be associated with older age (p < 0.001), female predominance (*p* = 0.004), higher NIHSS score (*p* = 0.006), and higher rate of previous stroke (p = 0.03). In general, patients with pre‐hospital sarcopenia showed poorer functional outcome after acute stroke than those with non‐sarcopenia (adjusted odds ratios: 5.15, 95% confidence interval: 1.35–19.7, *p* = 0.02).

**Conclusions:** Pre‐hospital sarcopenia is a predictor for poor functional outcome after acute stroke in elderly patients.

**1–47**

**The impact of sarcopenia and metabolic syndrome on cognitive performance in patients with COPD**

**Martijn van Beers** ^1^, Daisy Janssen^2,3^, Fiona Cleutjens^2^, Harry Gosker^1^, Frits Franssen^1,2^, Emiel Wouters^1,2^ and Annemie Schols^1^

^1^ *Department of Respiratory Medicine, NUTRIM School of Nutrition and Translational Research in Metabolism, Maastricht University Medical Centre, Maastricht, The Netherlands;* ^2^ *Department of Research & Education, CIRO, Centre of Expertise for Chronic Organ Failure, Horn, The Netherlands;* ^3^ *Centre of Expertise for Palliative Care, Maastricht University Medical Centre, Maastricht, The Netherlands*

**Rationale:** The prevalence of body composition abnormalities is high in patients with COPD, adversely influencing physical performance and cardiometabolic risk. Recent research also indicates that patients with COPD are prone to develop decreased cognitive performance. Here we investigated if cognitive performance is altered in COPD sub‐groups with metabolic syndrome (MetS) and/or sarcopenia.

**Methods:** A detailed neuropsychological test battery was administered to 170 patients with COPD referred for pulmonary rehabilitation. Sarcopenia was determined based on decreased appendicular skeletal muscle mass by dual‐energy x‐ray absorptiometry and impaired physical performance by 6‐minute walking distance. MetS was determined according to the NCEP ATP‐III criteria.

**Results:** The sample consisted of 170 patients (53.5% male), aged 63.4 ± 9.4 years, with an FEV_1_ of 54.5 ± 22.7%. Patients scored significantly worse than reference values derived from the Maastricht Aging Study (MAAS) on working memory (*Z* = −0.44, *p* < .001), verbal memory (*Z* = −0.33, *p* = .003) and cognitive flexibility (*Z* = −0.52, *p* < .001) but not on psychomotor speed and planning. MetS was observed in 54.7% of participants and sarcopenia in 34.7%. Differences in working memory, verbal memory and cognitive flexibility were most pronounced in the subgroup of non‐sarcopenic patients with MetS (*n* = 76) compared to the other patients. Within‐group analysis further revealed that patients with MetS scored significantly worse on the digit span backwards (working memory) test, compared to patients without MetS (*p* = .017).

**Conclusions:** This study suggests that presence of MetS may enhance risk for decreased cognitive performance in patients with COPD.

**1–48**

**Distinct skeletal muscle molecular responses to pulmonary rehabilitation in COPD: a cluster analysis**

**Anita E.M. Kneppers** ^1^, Roy A.M. Haast^2,3^, Ramon C.J. Langen^1^, Lex B. Verdijk^4^, Pieter A. Leermakers^1^, Harry R. Gosker^1^, Luc J.C. van Loon^4^, Mitja Lainscak^5,6^ and Annemie M.W.J. Schols^1^

^1^ *Department of Respiratory Medicine, NUTRIM School of Nutrition and Translational Research in Metabolism, Maastricht University Medical Centre+, Maastricht, the Netherlands;* ^2^ *Department of Cognitive Neuroscience, Faculty of Psychology and Neuroscience, Maastricht University, Maastricht, the Netherlands;* ^3^ *Maastricht Centre for Systems Biology, Maastricht University, Maastricht, the Netherlands;* ^4^ *Department of Human Biology, NUTRIM School of Nutrition and Translational Research in Metabolism, Maastricht University Medical Centre+, Maastricht, the Netherlands;* ^5^ *Division of Cardiology, General Hospital Murska Sobota, Murska Sobota, Slovenia;* ^6^ *Faculty of Medicine, University of Ljubljana, Ljubljana, Slovenia*

**Rationale:** Pulmonary rehabilitation (PR) forms a cornerstone in the management of COPD, targeting skeletal muscle to improve functional performance. However, there is substantial inter‐individual variability in the effect of PR on functional performance, which cannot be fully accounted for by generic phenotypic factors.

**Objectives:** To identify groups with a differential response to pulmonary rehabilitation, we performed an unbiased integrative analysis of the skeletal muscle molecular responses to rehabilitative training in COPD patients.

**Methods:** *M. vastus lateralis* biopsies were obtained from 51 COPD patients (FEV_1_%pred., 34 (26–41)) before and after short‐term high‐intensity supervised in‐patient PR. Muscle molecular markers were grouped by network‐constrained clustering, and their relative changes in expression values — assessed by qPCR and Western blot — were reduced to process scores by principal component analysis. Patients were subsequently clustered based on these process scores. Pre‐ and post‐PR functional performance were assessed by incremental cycle ergometry and 6‐min walking test (6MWT).

**Results:** Two clusters differed in PR‐induced Autophagy, Myogenesis, Glucocorticoid signalling, and Oxidative metabolism regulation, with Cluster 1 (C1) overall displaying more pronounced changes in marker expression than Cluster 2 (C2). General baseline characteristics did not differ between clusters. However, the functional improvements were more pronounced in C1, as a higher percentage of patients exceeded the minimal clinically important differences in peak workload (61 *vs* 21%, *p* < 0.05), and both peak workload and 6MWT (52 *vs* 8%, *p* < 0.01) upon PR.

**Conclusions:** We identified patient groups with distinct skeletal muscle molecular responses to pulmonary rehabilitation, associated with differences in functional improvements.

**1–49**

**The association between sarcopenia and the prognosis of cirrhotic patients in liver transplantation**

**Mimosa Nguyen** ^1^, Mélanie Tremblay^2^, Geneviève Huard^3^, An Tang^3^, Christopher F. Rose^2^ and Chantal Bémeur^1,2^

^1^ *Department of nutrition, Université de Montréal, Montreal, Quebec, Canada;* ^2^ *Centre de Recherche du Centre hospitalier de l'Université de Montréal, Montreal, Quebec, Canada;* ^3^ *Centre hospitalier de l'Université de Montréal, Montreal, Quebec, Canada*

**Introduction:** Sarcopenia affects up to 70% of patients suffering from chronic liver disease (cirrhosis). The presence of sarcopenia may influence the prognosis of cirrhotic patients before and after liver transplantation (LT). Few studies have assessed the evolution of sarcopenia in LT. The goal of this study was to follow the evolution and assess the impact of sarcopenia on the prognosis of cirrhotic patients before and after LT.

**Methods:** Skeletal muscle index (SMI) was calculated from cross‐sectional muscle area at the third lumbar level (L3) on computed tomography (CT). The following CT‐scans were analysed: before LT + before discharge and/or nearest 1‐year post‐LT. Sarcopenia was defined using previously published cutoffs based on gender. The association of sarcopenia with prognostic factors (mortality, hospital stay, infections, readmissions) was assessed in cirrhotic patients who underwent LT.

**Results:** Thus far, the average SMI before LT of sarcopenic and non‐sarcopenic patients were 40.3±5.3 cm2/m2 and 58.7±13.7 cm2/m2, respectively. The correlation of SMI with length of hospital stay, infections, and readmissions were high (rspearman = −0.714 *p* = 0.071), moderate (rspearman = −0.598 *p* = 0.156), and low (rspearman = −0.386 *p* = 0.393).

**Conclusions:** Preliminary results indicate that low muscle mass before LT tends to be associated with prolonged hospitalization. As we analyse the remaining data, the strength of the relationship between sarcopenia and the prognosis in LT will help better guide patient care.

**1–50**

**The correlation between liver steatosis and phase angle in patients with chronic intestinal failure**

**Taja Jordan**, Peter Popovič and Nada Rotovnik Kozjek

*UMC Ljubljana, Ljubljana, Slovenia*

**Introduction:** In patients with chronic intestinal failure who are on total parenteral nutrition, a common late complication is liver steatosis (LS), which can lead to the intestinal failure associated liver disease (IFALD). The phase angle (PA), measured with bioelectrical impedance analysis (BIA), is a good indicator of cellular mass status and integrity or functionality of the cellular membrane and is related to inflammatory status.

**Methods:** From January 2017 until December 2018 we are prospectively collecting data of patients with chronic intestinal failure, who are on home parenteral nutrition (HPN) therapy. LS is diagnosed with magnetic resonance imaging (MRI). BIA measurements are done at every routine visit.

**Results:** We have collected data from 53 patients, 22 men (42%) and 31 women (58%), the median age is 64,6 years (min. 23 years, max. 81 years). The median length of HPN therapy, calculated to the date of MRI, is 627 days (min. 82 days, max. 3374 days). The prevalence of LS is 32% (17 patients). The average PA in the group of patients with LS is 4,46**°** and in the group without LS 4,5**°**; p = 0,49 (Figure 1). The PA in female patients with, compared to female patients without LS is 3,9**°** vs. 4,39**°**; p = 0,27. The PA in male patients with, compared to male patients without LS is 5,1**°** vs. 4,6**°**; p = 0,26.

**Conclusions:** In our data, there is no significant correlation between PA and LS. There is a trend towards lower PA in patients with LS in the mixed group of patients and male patients. This supports the theory that LS is a proinflammatory state and can be detected by PA, which is inversely related to inflammation. Further studies with bigger data set are needed to confirm these findings.

**Figure 1:** Distribution of PA values in patients with LS (GraphA) and patients without LS (GraphB).

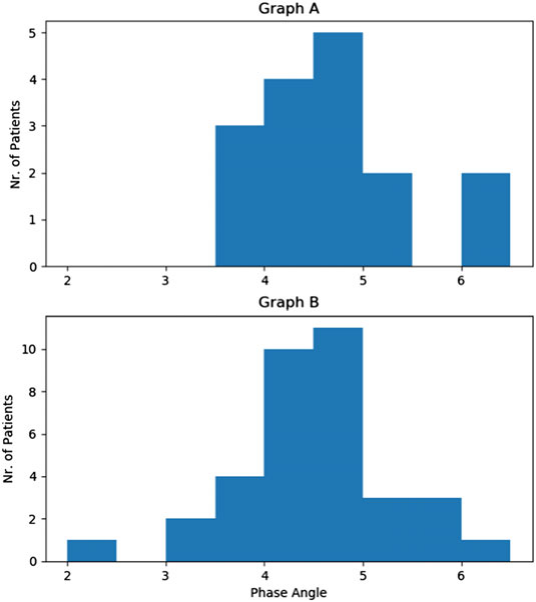

**1–52**

**Sarcopenia and cardiovascular risk indices in chronic kidney disease patients on conservative and replacement therapy**

**Alessio Molfino** ^1^, Silvia Lai^1^, Paola Andreozzi^2^, Alessandro Sgreccia^2^, Sabrina De Leo^1^, Luca Poli^3^, Renzo Pretagostini^3^, Paolo Protopapa^1^, Maria Ida Amabile^1^ and Maurizio Muscaritoli^1^ and Study Group on Geriatric Nephrology of the Italian Society of Nephrology (SIN) MIOSIN Study, preliminary results

^1^ *Department of Clinical Medicine, Sapienza University of Rome, Rome, Italy;* ^2^ *Department of Cardiovascular, Respiratory, Nephrological, Anaesthetic and Geriatric Sciences, Sapienza University of Rome, Rome, Italy;* ^3^ *Department of General and Specialized Surgery “Paride Stefanini”, Sapienza University of Rome, Rome, Italy*

**Introduction:** Chronic kidney disease (CKD) is a condition with high cardiovascular mortality, associated with emerging risk factors including sarcopenia. Several mechanisms can affect muscle in CKD, as vitamin D deficiency, low protein intake, physical inactivity, metabolic acidosis, and inflammation, leading to a worsening of cardiovascular outcomes and cognitive function.

We aimed to evaluate the prevalence of sarcopenia, in CKD patients on conservative and replacement therapy and the associations between sarcopenia and markers of atherosclerosis, endothelial dysfunction, and psychological and cognitive function.

**Methods:** We enrolled CKD patients (stage 3/5 KDOQI) and hemodialysis, peritoneal dialysis and post‐kidney transplantation patients. Clinical, laboratory and instrumental examinations, including bioimpedance analysis (BIA), hand‐grip strength, intima media thickness (IMT), flow mediated dilation (FMD) and evaluation of epicardial adipose tissue (EAT) were performed, as well as analysis of psychological and cognitive status by Montreal Cognitive Assessment (MoCA), Mini‐Mental State Examination (MMSE), and Geriatric Depression Scale (GDS). Parametric and non‐parametric tests were performed, as appropriate.

**Results:** 77 CKD patients (32 male), with a mean age of 69.57 ± 9.84 years, were studied. According to validated criteria by BIA, the prevalence of sarcopenia was of 49.4%. Sarcopenic patients showed higher values of IMT IMT (*p* = 0.032), EAT (*p* = 0.012), and lower FMD (*p* = 0.002), total‐cholesterol (*p* = 0.005), and HDL‐cholesterol (*p* = 0.008) with respect to non‐sarcopenic patients. By cognitive tests, we observed higher GDS score (*p* = 0.04) in sarcopenic patients with respect to non‐sarcopenic patients, whereas we did not find differences between the 2 groups in MMSE score and in MoCA score (*p* = 0.12, p = 0.07, respectively).

**Conclusions:** Sarcopenia is highly prevalent in CKD patients and is significantly associated with variations in early systemic markers of atherosclerosis and endothelial dysfunction, known as negative prognostic markers that may worsen the prognosis.

**1–53**

**Association of energy expenditure with sarcopenia diagnosis components in patients on maintenance hemodialysis**

**Karina de Jesus Antonio** ^1^, Barbara Perez Vogt^2^, Vanessa Benzoni Venitelli^3^ and Jaqueline Costa Teixeira Caramori^4^

^1^ *Post graduation in Pathophysiology in internal medicine, UNESP, Botucatu, SP, Brazil;* ^2^ *University of Western São Paulo – Unoeste, Presidente Prudente, SP, Brazil;* ^3^ *Graduation in nutrition, UNESP, Botucatu, SP, Brazil;* ^4^ *Department of Internal Medicine, Faculdade de Medicina de Botucatu, UNESP, Botucatu, SP, Brazil*

**Introduction:** Sarcopenia is defined as generalized and progressive decline of muscle mass and strength, and it is related to chronic kidney disease (CKD). Several factors in CKD may lead to muscle mass and function loss, such as increased inflammation, energy and protein intake decrease, hormonal disarrangements, and physical inactivity, resulting in sarcopenia. Those factors may also affect energy expenditure.

**Aim:** to verify the association of resting energy expenditure (REE) with sarcopenia of patients on maintenance hemodialysis.

**Methods:** Cross‐sectional study, which included hemodialysis patients >18 years old. REE was assessed by indirect calorimetry, muscle mass was assessed by multifrequencial bioimpedance and muscle mass index (MMI) was calculated (total muscle mass/height^2^). Muscle strength and function were evaluated using handgrip strength (HGS) and gait speed in 4 m walking distance, respectively. Spearman's correlation was used to verify the correlation between REE and biochemical and nutritional parameters. Multiple linear regression was performed to verify the association of REE with sarcopenia components (MMI, HGS, and gait speed).

**Results:** Thirty‐two patients were included, 56.3% male, mean age 64 ± 13 years, dialysis vintage 36 (25;52) months, BMI 27.2 ± 6.2 kg/m^2^, serum creatinine 9 ± 2.8 mg/dl, REE 1486 (1077;1800) kcal, HGS 15(9.2;27.5), MMI 13.4 ± 4.0 kg/m^2^, gait speed 7.6 (5.8;14.9) sec. There was positive correlation between REE and BMI (*r* = 0.479; *p* = 0.006), HGS (*r* = 0.607; *p* < 0.001) and MMI (*r* = 0.613; p < 0.001), and negative correlation between REE and age (*r* = −0.482; p = 0.005), gait speed (r = −0,633; p < 0.001). In the linear regression adjusted for age and sex, REE was associated only with HGS (β = 0.476; 95% CI 2.60–39.26; *p* = 0.027).

**Conclusions:** Muscle strength and mass were positively associated with REE, while muscle function, assessed by gait speed, was negatively associated with REE. HGS is associated with REE, independent of age and sex, showing an important influence of strangth in energy expendure, and not only muscle mass.

**1–54**

**Sarcopenia in peritoneal dialysis: prevalence and associations**

**Maryanne Zilli Canedo Silva** ^1^, Barbara Perez Vogt^2^, Nayrana Soares Do Carmo Reis^1^ and Jaqueline Costa Teixeira Caramori^3^

^1^ *Post graduation in Pathophysiology in internal medicine, UNESP, Botucatu, SP, Brazil;* ^2^ *University of Western São Paulo – Unoeste, Presidente Prudente, SP, Brazil;* ^3^ *Department of Internal Medicine, Faculdade de Medicina de Botucatu, UNESP, Botucatu, SP, Brazil*

**Introduction:** Peritoneal dialysis (PD) has some particularities that leads to nutritional disarrangements, increasing the risk of sarcopenia. Aim: to evaluate the prevalence of sarcopenia in PD patients and the association of its components with clinical, nutritional and physical capacity parameters.

**Methods:** Cross‐sectional study including PD patients>18 years old, with assessments of appendicular skeletal muscle mass index (ASMMI) by DXA, muscle strength by handgrip strength (HGS), and physical performance by 4 m gait speed (GS). European Working Group on Sarcopenia in Older People (EWGSOP) criteria was used for sarcopenia diagnosis. Groups were compared by Student's T‐test, Mann–Whitney and chi‐square. Variables with significance<0.2 in comparisons were added to multiple logistic regression.

**Results:** Fifty patients were included, mean age 55.7 ± 16.2 years, 52% female. Sarcopenia prevalence ranged from 2–4%, according to the variables used for diagnosis. Patients were divided according to ASMMI, HGS and GS cutoffs proposed by the EWGSOP criteria and compared. There were no differences between patients with ASMMI > and < cutoff. Sex (60% vs 30% male, *p* = 0.038), age (49 ± 14 vs 66 ± 15 years, p < 0.001), GS (0.95 ± 0.19 vs 0.69 ± 0.21 m/s, p < 0.001), ASMMI [7.52 (6.40–8.84) vs 6.40 (6.05–7.21), *p* = 0.024], SPPB (10(9–11) vs 8(7–10), *p* = 0.003) and serum creatinine (10 ± 2.8 vs 7.7 ± 2.1 mg/dl; p = 0.003) were different between patients with HGS > and < cutoff. Age (50 ± 13 vs 65 ± 17 years, *p* = 0.001), HGS [28(20–40) vs 18(12–24), p = 0.002], SPPB [10(9–11) vs 8(7–8), p < 0.001], serum creatinine (9.7 ± 2.7 vs 8.1 ± 2.8 mg/dl, *p* = 0.041), albumin (3.8 ± 0.3 vs 3.4 ± 0.5, *p* = 0.004) were different between patients with GS > and < 0.8 m/s. In multiple analysis, HGS was associated with ASMMI (OR:0.474; 95%IC 0.242–0.928; *p* = 0.029) and GS (OR:0.004; 95%IC 0.000–0.549; *p* = 0.028). No association among ASMMI or GS with selected variables were found.

**Conclusions:** Prevalence of sarcopenia varies according the criteria. HGS was the component of sarcopenia that had associations with clinical and nutritional parameters, mainly related to muscle mass and function.

**1–55**

**The natural course of body composition in hemodialysis patients**

**Wesley J. Visser** ^1^, Anneke M.E. van Egmond^1^ and Ewout J. Hoorn^2^

^1^ *Division of Dietetics;* ^2^ *Nephrology and Transplantation, Department of Internal Medicine, Erasmus MC, Rotterdam, The Netherlands*

**Introduction:** Protein‐energy wasting and subsequent muscle wasting is a major complication in chronic kidney disease and even more pronounced in patients on hemodialysis. Because data addressing the effect of dialysis on body composition are limited, our aim was to address the natural course in body composition.

**Methods:** All chronic hemodialysis patients in our university center were eligible for this study (regardless of dialysis vintage and except for life expectancy ≤6 months, malignancy or infection). During 5 months we performed monthly measurements of body weight, body composition (lean tissue mass [LTM] and adipose tissue mass [ATM]), handgrip strength, and serum albumin and CRP. Mixed models were used to analyse the natural course in body composition.

**Results:** 54 patients were included (median age 65 years, dialysis vintage 19 months, 71% male). During the study period body weight decreased 0.5 ± 0.03 kg, which was caused by a decrease in LTM (6.5 ± 0.6 kg) and an increase in ATM (4.5 ± 0.3 kg). A concurrent decrease in handgrip strength and serum albumin and increase in CRP were observed. At baseline, LTM (in kg) was significantly lower in patients >65 years (35 ± 0.06 vs. 41.9 ± 0.7, p = 0.01), women (27.1 ± 0.6 vs. 42.8 ± 0.8, p = 0.001), and patients with a dialysis vintage >12 months (35.8 ± 0.1 vs. 42.3 ± 0.7, p = 0.03). For these subgroups, the change in LTM over time was not greater (*p* > 0.05 for all). Of interest, in patients with LTI ≥ P10 at baseline LTM (in kg) decreased significantly more than in patients with LTI < P10 (−1.8 ± 0.1 vs. ‐0.5 ± 0.04, p = 0.001).

**Conclusions:** Hemodialysis has a major impact on body composition regardless of dialysis vintage. The change in body weight does not reflect the change in body composition.

**1–56**

**Validation of the Korean version of the SARC‐F in peritoneal dialysis patients**

**Seok Hui Kang** ^1^, Won Suk An^2^, Jun Chul Kim^3^ and Soo Jeong Choi^4^

^1^ *Division of Nephrology, Department of Internal Medicine, Yeungnam University Hospital, Daegu, Republic of Korea;* ^2^ *Department of Internal Medicine, Dong‐A University Hospital, Pusan, Republic of Korea;* ^3^ *Department of Internal Medicine, CHA Gumi Medical Center, CHA University School of Medicine, Republic of Korea;* ^4^ *Division of Nephrology, Department of Internal Medicine, Soonchunhyang University Bucheon Hospital, Republic of Korea*

**Background:** The SARC‐F is a simple screening tool for sarcopenia, consisting of 5 questions covering strength, assistance in walking, rising from a chair, climbing stairs, and falls. Low muscle mass and poor physical functions are frequent found in peritoneal dialysis (PD) patients. This study aimed to validate the Korean version of the SARC‐F in PD patients.

**Methods:** The Korean version of SARC‐F, which was introduced from a previous study validating it in community‐dwelling older adults, was tested in regular visiting outpatients on PD. According to the diagnostic criteria of European Working Group on Sarcopenia in Older People, Asian Working Group on Sarcopenia, and the Foundation for the National Institutes of Health Sarcopenia Project, sarcopenia was defined as low muscle mass and diminished strength. The subjects were divided into 2 groups, according to the sum of the SARC‐F score (<4 and ≥ 4).

**Results:** All 127 PD patients were included in our study. Mean age was 55.5 ± 11.7 years. The numbers of men and women were 67 and 60, respectively. The prevalence of sarcopenia in men and women according to criteria of the SARC‐F was 14.9% and 31.6%, respectively. For men, compared with other diagnostic criteria, SARC‐F showed low sensitivity (33.3~66.7%) and low positive predictive value (PPV) [10.0~20.0%], but high specificity (85.9~87.5%) and high negative predictive value (NPV) [94.7~98.2%]. Similarly, for women, SARC‐F showed low sensitivity (50.0~80.0%), low PPV (5.3~21.1%) and high NPV (97.6%).

**Conclusions:** High SARC‐F score showed a high specificity and high NPV, but low sensitivity and low PPV. This simple method may be useful for ruling out sarcopenia in PD patients.

**1–57**

**Serum myostatin levels are associated with abdominal aortic calcification, skeletal muscle mass and bone mineral density in dialysis patients**

**Won Suk An** ^1^, Su Mi Lee^1^, Soo Jeong Choi^2^ and Jun Chul Kim^3^

^1^ *Department of Internal Medicine, Dong‐A University, Republic of Korea;* ^2^ *Division of Nephrology, Department of Internal Medicine, Soonchunhyang University Bucheon Hospital, Republic of Korea;* ^3^ *Department of Internal Medicine, CHA Gumi Medical Center, CHA University School of Medicine, Republic of Korea*

**Introduction:** Serum myostatin levels are increased according to renal function decline and myostatin is main mediator of chronic kidney disease‐related sarcopenia. A previous study reported that serum myostatin levels are negatively associated with abdominal aortic calcification (AAC) in older men. However, no research has been conducted on the relationship between myostatin levels and vascular calcification (VC) in dialysis patients. The aim of this study was to assess the association between serum myostatin levels and AAC in dialysis patients. In addition, we analysed the relationship between serum myostatin levels, muscle mass and bone mineral density.

**Methods:** In this cross‐sectional study, we evaluated AAC on the lateral lumbar spine using plain radiograph and bone mineral density (BMD) in 71 patients undergoing dialysis. We classified patients into two groups according to the median value of myostatin: patients with high myostatin levels (≥4991.4 pg/mL) and those with low myostatin levels (<4991.4 pg/mL).

**Results:** The proportion of patients with an abdominal aortic calcification (AAC) score ≥ 5 was higher in patients with low myostatin levels compared with that in patients with high myostatin levels. The median myostatin levels for patients with AAC scores of ≥5 were 4073.5 pg/mL, whereas those for patients with AAC scores of <5 were 5838.6 pg/mL. The myostatin levels were negatively associated with AAC scores on plain radiograph and had a positive association with appendicular skeletal muscle mass and T‐scores for BMD measured at the total hip and femur neck. Lower myostatin levels were independently associated with higher AAC scores after adjustment for age, gender, diabetes mellitus, dialysis vintage, dialysis modality, and osteoprotegerin levels.

**Conclusions:** Lower serum myostatin levels were associated with higher AAC scores, lower muscle mass and lower bone mineral density in dialysis patients. Further studies are necessary to validate these findings in large cohorts and prospective studies.

**1–60**

**Sarcopenia and frailty in cancer survivors after transcatheter aortic valve implantation**

**Masakazu Saitoh** ^1^, Mike Saji^2^, Yudai Suzuki^1^, Takeshi Arimitsu^1^, Akihiro Sakuyama^1^ and Masatoshi Nagayama^2^

^1^ *Department of Rehabilitation, Sakakibara Heart Institute, Tokyo, Japan;* ^2^ *Department of Cardiology, Sakakibara Heart Institute, Tokyo, Japan*

**Background:** The targets of this study were to evaluate the prevalence of sarcopenia or frailty and to investigate the clinical outcomes for cancer survivors who underwent transcatheter aortic valve implantation (TAVI).

**Methods and Results:** We retrospectively enrolled 213 patients undergoing elective TAVI procedures. Sarcopenia was assessed by Asian Working Group for Sarcopenia (AWGS) criteria. Various frailty scales including the Rockwood clinical frailty scale, modified essential frailty toolkit, the frailty index, and the SPPB score were evaluated. The primary endpoint was the functional decline determined by decreased Short physical performance battery (SPPB) summary score before discharge, comparing with the preoperative score. Patients who were diagnosed with cancer, or have had cancer treatment, were defined as cancer survivors, and variables were compared between cancer survivors and controls. The prevalence of sarcopenia in cancer survivors was significantly higher than that in controls (71% vs. 50%, *p* = 0.017). In addition, overall prevalence of frailty varied from 22 to 51%, and was approximately 1.5‐fold higher in cancer survivors compared with controls undergoing TAVI. In univariate analysis, cancer survivors and underweight associated with functional decline after TAVI (OR: 2.609, 95% CI 1.296 to 5.250, *p* = 0.007, and OR 2.2218, 95%CI 1.005 to 4.896, *p* = 0.049). In multivariate Cox regression analysis, cancer survivors were independently associated with functional decline after adjusting for confounding variables (HR2.53, 95% CI 1.218 to 5.254, *p* = 0.013).

**Conclusions:** The prevalence of sarcopenia or frailty syndrome, which indicated loss of homeostatic or physical functional reserve, was higher in cancer survivors, and may confer greater risk of functional decline following TAVI.

**1–61**

**Sarcopenia and frailty phenotype influence on quality of life of patients with bladder or kidney cancer**

Patrícia Fonseca dos Reis^1,2^, Mylena Pinto dos Santos^2^ and **Renata Brum Martucci** ^1,2^

^1^ *Nutrition Institute, Rio de Janeiro State University, Rio de Janeiro, Brazil;* ^2^ *Nutrition and Dietetic Service, National Cancer Institute, Rio de Janeiro, Brazil*

Sarcopenia is the progressive loss of muscle mass, which is a key component of frailty; both increase the risk of adverse events such as physical disability, quality of life (QoL) reduction and death. Sarcopenia and frailty are overlapping syndromes, and they are associated to diseases such as cancer. The aim of this study was to evaluate the influence of sarcopenia and frailty phenotype on QoL of bladder or kidney cancer patients.

**Methods:** Cross sectional study with bladder or kidney cancer patients, aged 20 years or more. Clinical and nutritional data were collected from medical records and nutritional appointment. Sarcopenia was defined as low skeletal muscle mass < 6.76 kg/m^2^ for women and < 10.76 kg/m^2^ for male patients assessed by bioelectrical impedance analysis. Frailty was defined as the presence of 3 or more of the following criteria: unintentional weight loss (≥5% of body weight in prior year), self‐reported exhaustion, weakness (low handgrip strength), slowness (gait speed) and low physical activity (IPAQ questionnaire). QoL was assessed using the EORTC QLQ‐C30 questionnaire.

**Results:** 40 patients were evaluated (50% bladder and 50% kidney cancer); mean age 62; 62.5% male; 62.5% sarcopenic; 25% frail and 52.5% pre‐frail. There were no differences in nutritional parameters, performance and muscular strength, body composition, physical activity, and QoL, according to tumour location. As QoL was not different in sarcopenic compared to non‐sarcopenic patients. However, a worse QoL was observed in non‐frail compared to frail patients, in Global Health Status (84.2 ± 18.8 vs 55.8 ± 19.3; *p* = 0.03); Role Function (100 vs 38.3 ± 45.2; *p* < 0.001); Physical Function (96.3 ± 5.9 vs 52 ± 30.3; p < 0.001); Fatigue (2.9 ± 4.9 vs 56.7 ± 40; *p* = 0.001); Pain (1.8 ± 5.5 vs 78.3 ± 33.4; p < 0.001) and Appetite lost (3.7 ± 11.1 vs 40 ± 46.6; p = 0.001).

**Conclusions:** Frailty influenced a worse quality of life in bladder or kidney cancer patients, although sarcopenia has not exerted the same effect.

**1–63**

**Prevalence of cardiac cachexia in cats with heart failure**

Sasha Santiago, **Lisa M. Freeman** and John E. Rush

*Cummings School of Veterinary Medicine, Tufts University, North Grafton, MA, USA*

**Background and Aims**: Naturally‐occurring cardiomyopathies affect 10–15% of all pet cats. Congestive heart failure (CHF) is a common result of cardiomyopathy in cats that can be associated with cardiac cachexia. Studies have shown that 48–54% of dogs with CHF are affected by cachexia (defined as loss of muscle). No studies have been conducted to investigate the prevalence of cardiac cachexia in cats with CHF. Therefore, the aim of this study was to determine prevalence of cardiac cachexia in cats with CHF due to cardiomyopathies.

**Methods**: Cats with CHF evaluated by the Cardiology Service at the Cummings School of Veterinary Medicine between June, 2015‐May, 2018 were eligible. Cats with CHF due to hypertrophic, dilated, unclassified, restrictive, or arrhythmogenic right ventricular cardiomyopathies were enrolled. Data from the medical records was retrospectively reviewed, including body weight, body condition score (BCS), and muscle condition score (MCS). Body condition score, which assesses fat stores, was measured on a 1–9 scale, with 1 = emaciated, 9 = obese, and 4–5 considered ideal. Muscle condition was categorized using the validated World Small Animal Veterinary Association scoring system of normal muscle, mild muscle loss, moderate muscle loss, or severe muscle loss.

**Results**: Median age of the cats (*n* = 137) was 10.0 years (range, 0.7–18.7 years); 72% were male. Underlying diseases included hypertrophic (*n* = 119), dilated (*n* = 8), unclassified (*n* = 7), arrhythmogenic right ventricular (*n* = 2), and restrictive (n = 1) cardiomyopathies. Mean body weight was 5.2 kg (range, 2.3–9.5 kg). Only 12% of cats were underweight (BCS <4/9) and 30% of cats were overweight or obese (BCS > 5/9). However, muscle loss was identified in 45% of cats. Twenty‐eight percent of cats had mild muscle loss, 12% had moderate muscle loss, and 4% had severe muscle loss.

**Conclusions**: Although many cats were overweight or obese, cachexia was present in 45% of cats with CHF.

**1–64**

**Age‐related defects at the motoneuron nuclear envelope may be drivers of skeletal muscle atrophy**

**Ashley P. Gillon** ^1^, Jon C. Cornwall^2^ and Phillip W. Sheard^1^

^1^ *Department of Physiology School of Biomedical Sciences, University of Otago, New Zealand;* ^2^ *Otago Medical School, University of Otago, New Zealand*

**Study Purpose:** Life expectancy continues to extend, frailty caused by loss of skeletal muscle mass shows continual growth. Many studies have shown that age‐related loss of skeletal muscle is driven at least partly by denervation. Although we have identified no single cause of denervation in old age, recent data suggests degradation of the nucleocytoplasmic barrier and nuclear envelope transport process are responsible for nerve loss in a number of neurodegenerative diseases, so we ask whether similar defects accompany motoneuron death in normal aging.

**Methods:** We used immunohistochemistry on young and old mouse tissues to explore potential links between motoneuron death and nucleocytoplasmic transport regulatory proteins, and we used a nuclear permeability assay to investigate the patency of the nuclear barrier on extracts of spinal cord from young and old mice.

**Results:** We found that loss of lumbar motoneurons in old age was accompanied by reductions in immunodetectable levels of key nucleocytoplasmic transport proteins and increased nuclear permeability.

**Conclusions:** Our results suggest that emergent defects in nucleocytoplasmic transport and in the integrity of the nuclear barrier may contribute to age‐related motoneuron death and therefore these events may be significant indirect drivers skeletal muscle loss.

**1–65**

**Myofibre death is not a major contributor to age‐related skeletal muscle atrophy**

Navneet Lal^1^, Jon Cornwall^2^ and **Philip Sheard** ^1^

^1^ *Department of Physiology, Otago University, Dunedin, New Zealand;* ^2^ *Centre for Early Learning in Medicine, Otago University, Dunedin, New Zealand*

**Introduction:** Age‐related skeletal muscle atrophy is thought to result partly from loss of myofibres. Myofibre loss is generally inferred from myofibre counts from whole muscles, but evidence for decline in fibre number varies between muscles. In order to reliably determine the magnitude of fibre loss in widely studied mouse muscles, we performed a detailed analysis of age‐related change in fibre number and muscle microarchitecture.

**Methods:** Six young and ten old mouse soleus, biceps brachii, extensor digitorum longus (EDL), sternomastoid, and tibialis anterior muscles were stained and imaged to permit measurement of tendon lengths. Myofibre number was established by counting right sided muscles from standard transverse sections whilst left sided muscles were counted from sections taken obliquely to exclude tendons.

**Results:** The intramuscular terminations of proximal and distal tendons in young soleii were separated by 600 ± 400 μm, but tendon elongation resulted in tendon overlap of 310 ± 350 μm in the mid‐belly region in old muscles. Myofibre counts from mid‐belly transverse sections revealed ~15% reduction between young (1023 ± 65) and old (889 ± 162) soleii, but this apparent fibre loss was abolished from oblique sections that excluded the myotendinous region (1075 ± 58 vs 1023 ± 105). Similar results were observed in EDL muscles but no age‐related changes to morphology or fibre number were evident in other muscles.

**Conclusions:** Age‐related tendon expansion in soleus and EDL reduces the probability that all myofibres can be included for counting in a single transverse section, and this likely results in erroneous indication of fibre loss between young and old muscles. Our findings call into question the existence of myofibre death as a significant contributor to age‐related muscle atrophy.

**1–66**

**p62 accumulates in late stage autophagic organelles within the myotendinous regions of elderly mouse skeletal myofibers**

**Navneet Lal** ^1^, Jon Cornwall^2^, Tania Slatter^3^, David Rowlands^4^ and Philip Sheard^1^

^1^ *Departments of Physiology, Otago University, Dunedin;* ^2^ *Centre for Early Learning in Medicine, Otago University, Dunedin;* ^3^ *Pathology, Otago University, Dunedin;* ^4^ *School of Sport and Exercise, Massey University, New Zealand*

**Introduction:** Losses in muscle mass and strength are normal correlates of aging, however, our understanding of the processes that underpin these phenomena is incomplete. As critical sites of force transmission, alterations to myotendinous junction (MTJ) structure could contribute to losses in muscle strength and we recently described such changes in elderly mice. To advance our understanding of the aberrant processes involved in MTJ remodelling, this study investigated autophagic processes in elderly myofibres.

**Methods:** 6 young and 6 elderly (6 and 24 months) mouse soleus muscles were cryosectioned and immunostained to visualize autophagosomal and lysosomal proteins (LC3b and LAMP2 respectively), and the autophagic substrate sequestering protein, p62. MTJ and non‐MTJ regions were identified using WGA staining. Three‐dimensional quantification of p62 immunofluorescence among autophagic compartments was conducted using confocal microscopy.

**Results:** In non‐MTJ regions of young muscles, the highest fractions of p62 were found within the sarcoplasm (86 ± 7%) with decreasing fractions found in autophagosomes (12 ± 2%) and autolysosomes (2 ± 1%). However, fractions found in autophagosomes (24 ± 3%) and autolysosomes (11 ± 2%) of MTJs were two‐fold higher than non‐MTJ regions (*p* < 0.05 and < 0.01 respectively). By contrast, the distribution of p62 among autophagic organelles in both MTJ and non‐MTJ regions of elderly myofibres mirrored that of young MTJ regions, indicating that autophagic processes normally localized to MTJs became distributed further along the length of myofibres. Moreover, p62 accumulated specifically in late stage autophagic organelles within myofibres that contained the highest total amounts of p62.

**Conclusions:** MTJs are significant producers of autophagic substrate. In elderly myofibres, the process of sequestering substrate into autophagic organelles appears intact though its accumulation in late stage autophagic organelles indicates ineffective degradation. This work links our prior observations of MTJ elongation with autophagic insufficiency; therefore, highlights a possible pathogenic relationship between the two events.

**1–67**

**Effect of acetic acid intake on muscle quality and expressions of atrophy‐related genes of aged rats**

**Hitomi Maruta**, Reina Abe, Yun Ma, Chiaki Isono and Hiromi Yamashita

*Department of Nutritional Science, Faculty of Health and Welfare Science, Okayama Prefectural University, Soja‐city, Okayama, Japan*

Skeletal muscle atrophy in elderly causes a risk of falling, leading to life‐style related diseases, and a loss of quality of life. Previously we showed that oral administered acetic acid improved obesity and enhanced oxygen consumption of OLETF rats, which are genetic model of obesity animals. And it increased genes expressions associated with lipid and glucose metabolisms in muscle of the rats. Acetic acid increased myoglobin, GLUT4, MEF2A, and PGC‐1a expressions through the activation of AMP‐activated protein kinase (AMPK) in cultured L6 myotube cells. Treatment of acetic acid reduced lipid accumulation and enhanced glucose uptake in L6 cells. In this study, we investigated the effect of acetic acid on skeletal muscles of aged rats to examine whether acetic acid contributes to improve the muscle quality of elderly.

Thirty‐six month‐old male SD rats were randomly assigned to 2 groups, water‐injected and 1% acetic acid‐injected groups. Acetic acid group was given 5 ml/kg BW of 1% *v*/v acetic acid, while water group was given 5 ml/kg BW of distilled water, until 56 week‐old.

Oxygen consumption rate was decreased in water administered rats with aging, however, that of acetic acid administered rats was not significantly changed. Population of Type I fibre in soleus muscle was significantly higher in acetic acid administered rats than that of water administered rats. Expressions of Atrogin1, MuRF1, and TGF‐b genes were significantly lower, while MEF2A and mitochondrial DNA were increased in soleus muscle of acetic acid administered rats than that of water administered rats.

It is suggested that chronic intake of acetic acid had the effect to improve quality of soleus muscle of aged rats.

**1–68**

**High‐intensity interval training improves oxidative stress and energetic metabolism in skeletal muscle of infarcted rats**

**Mariana Janini Gomes** ^1^, Luana Urbano Pagan^1^, Aline Regina Ruiz Lima^1^, Leiliane Rodrigues dos Santos Oliveira^1^, Thierres Hernani Dias de Pontes^1^, Eder Anderson Rodrigues^1^, Leonardo Antonio Mamede Zornoff^1^, Ana Angélica Henrique Fernandes^2^, Katashi Okoshi^1^ and Marina Politi Okoshi^1^

^1^ *Botucatu Medical School, Sao Paulo State University, UNESP, Botucatu, SP, Brazil;* ^2^ *Institute of Biociences of Botucatu, Sao Paulo State University, UNESP, Botucatu, SP, Brazil*

Current guidelines recommend regular physical exercise for patients with stable heart failure. Studies have suggested that greater benefits can be achieved by high‐intensity interval training (HIIT).

**Aim**: To evaluate the effects of HIIT on oxidative stress and energetic metabolism in skeletal muscle of rats with myocardial infarction (MI).

**Methods:** Three months after MI induction, 24 Wistar rats were divided into three groups: Sham, sedentary MI (MI‐Sed) and MI subjected to HIIT (MI‐HIIT). MI‐HIIT trained on treadmill 3 times per week for three months. Cardiac evaluation was performed by echocardiogram. Functional capacity was evaluated by maximum effort test. Oxidative stress and energetic metabolism were assessed in gastrocnemius muscle by spectrophotometry. Statistical analysis: ANOVA; *p* < 0.05.

**Results**: HIIT improved functional capacity (run distance: Sham 372 ± 54.9; MI‐Sed 308 ± 64.8; MI‐HIIT 484 ± 86.8 m) without changing echocardiographic parameters. Lipid hydroperoxide concentration was increased in both MI groups and reduced by HIIT (Sham 139 ± 20.2; MI‐Sed 227 ± 23.1; MI‐HIIT 201 ± 20.8 nmol/g tissue). Activity of the antioxidant enzyme catalase [Sham 26.2 (23.4–28.0); MI‐Sed 16.3 (12.5–17.7); MI‐HIIT 25.8 (23.5–26.6 μmol/g tissue] and glutathione peroxidase (Sham 68.9 ± 6.05; MI‐Sed 42.0 ± 6.57; MI‐HIIT 65.2 ± 7.46 nmol/mg protein) was decreased in MI‐Sed and normalized by HIIT. Phosphofructokinase activity was reduced in both MI groups and was higher in MI‐HIIT than MI‐Sed [Sham 161 (130–179); MI‐Sed 108 (73.7–120); MI‐HIIT 123 (120–140) nmol/g tissue]. Pyruvate dehydrogenase, citrate synthase and β‐hydroxy acyl CoA dehydrogenase activities were lower in MI‐Sed than Sham. Lactate dehydrogenase activity was increased in both MI groups and reduced by HIIT [Sham 82.9 (79.3–88.0); MI‐Sed 116 (110–129); MI‐HITT 87.0 (85.2–91.6) nmol/mg protein].

**Conclusions**: High‐intensity interval training improves functional capacity and ameliorates oxidative stress and energetic metabolism in gastrocnemius muscle of myocardial infarction rats.

*Financial support:* FAPESP and CNPq.

**2–01**

**The role of myogenin and HDAC4 in the regulation of E3‐ligases MuRF‐1 and MAFbx expression in rat soleus at the early stage of muscle atrophy**

**Ekaterina P. Mochalova** ^1^, Svetlana P. Belova^1^ and Tatiana L. Nemirovskaya^1,2^

^1^ *Institute of Biomedical Problems of Russian Academy of Sciences (IMBP RAS), Moscow, Russia;* ^2^ *Lomonosov Moscow State University, Moscow, Russia*

Skeletal muscle atrophy occurs in many chronic diseases and disuse conditions and results in a significant increase in the expression of the key markers of proteolysis such as E3‐ligases MuRF‐1 and MAFbx. We investigated if HDAC4 regulates the expression of MuRF1 and MAFbx through the myogenin activation following 1 and 3 days of hindlimb unloading. Twenty‐one male Wistar rats were divided into 3 groups (*n* = 7 per group): intact control (C), rats unloaded for 1 day (HU1) and 3 days (HU3). MuRF‐1 and MAFbx mRNA expression was significantly increased (1.4‐ and 1.9 fold, respectively, p < 0,05) in HU1 group compared to Control. One day HU induced a significant increase in HDAC4 content in nuclear fraction (p < 0.05), but didn't affect the content of myogenin. At the same time the content of pAkt (Ser 473) and pFOXO3a (S253) (factors that can regulate E3‐ligases expression) in HU1 was significantly decreased (by 60% and 45%, respectively, p < 0.05). MuRF‐1 and MAFbx mRNA expression was upregulated (3.8 and 6.1 fold, respectively, *p* ≤ 0.05) in HU3 group compared to Control. The level of HDAC4 in the nuclear fraction in HU3 was the same as in HU1 group but the level of myogenin mRNA expression in HU3 group was increased (2.5 fold, p ≤ 0.05) vs Control. The content of pAkt (S473) and pFOXO3(S253) was significantly decreased (by 60% and 45% respectively, p ≤ 0.05) in HU3 group compared to Control. These data suggest that (1) at the early stages of HU (1,3 days) MuRF‐1 and MAFbx mRNA expression is regulated by FoxO3 and (2) myogenin contribute to E3‐ligases regulation by the 3^rd^ day of HU.

*This work was supported by RSF (grant №18–15‐00062).*

**2–02**

**Walker‐256 tumour growth affects hepatic metabolomic profile more severely in young‐host compared to adult rats**

Lea Blanchard^1,2^, Carla de Moraes Salgado^1^, Rogerio William dos Santos^1^, Sarah Christine Pereira de Oliveira^1^, Bread Leandro Cruz^1^, Lais Rosa Viana^1^ and **Maria Cristina Cintra Gomes‐Marcondes** ^1^

^1^ *Laboratory of Nutrition and Cancer, Department of Structural and Functional Biology, Biology Institute, University of Campinas (UNICAMP), Brazil;* ^2^ *Université d'Angers, Angers, France*

**Introduction:** Cancer cachexia is related to marked body weight loss and intense metabolic changes, especially related to liver metabolic activity. We evaluated the tumour‐induced cachexia associated to hepatic metabolomic profile in young and adult Walker‐256 tumour‐bearing rats.

**Methods:** Adult (A; 90 days) and young (Y; 21 days) rats were implanted with tumour (2 x 10^6^ viable cells) and euthanised at pre‐agonic state: 14^th^ day of tumour evolution for young (YW), and 21^st^ day for adults (AW), comparing to non‐tumour‐bearing rats (YC; AC). The carcass, liver and tumour weights were measured. The metabolomic analysis of liver samples was accessed by high‐resolution ^1^H‐NMR spectroscopy.

**Results:** The YW rats had a decrease in carcass‐to‐initial body weight ratio (14% lower; *P* = 0.005), associated with a higher liver‐to‐carcass weight (18%; *P* < 0.0001), with 18% of tumour‐to‐carcass weight ratio. The AW group had a 9% decrease in carcass weight, similar liver‐to‐carcass weight ratio associated with 18% of tumour‐to‐carcass weight ratio. The cachexia index was higher in young group (Y > A, 1.4‐times; *P* = 0.0001). The liver metabolomic profile showed important differences between tumour‐bearing and control groups (YW > YC; AW>AC; P < 0.0001) related to glucose‐alanine cycle, ammonia recycling and gluconeogenesis metabolism. We observed higher hepatic metabolomic profile and impacted pathways mainly in YW compared to AW groups, as the ^1^H‐NMR analysis of liver tissue showed differences mainly in the content of betaine (YW > AW, 2.8x higher; P < 0.0001), β‐alanine (YW > AW, 2.1x; *P* = 0.0010), pyruvate (YW > AW, 1.6x, *P* = 0.05), and other metabolites. These differences showed some impacted metabolic in liver function such as nitrogen metabolism (*P* = 0.0007); alanine/aspartate/glutamate metabolism (P = 0.0001), histidine metabolism (*P* = 0.0021) and β‐alanine metabolism (*P* = 0.0035) (analysed using http://www.metaboanalyst.ca).

**Conclusions:** The young tumour‐bearing animals had short survival (14 days) associated to higher hepatic activity (gluconeogenesis and proteolysis process), compared to adult group (21 days), leading to severe implications in the young host.

*Support: Fapesp, CNPq, FAEPEX‐UNICAMP*.

**2–03**

**Effects of mitochondrial targeting with SS‐31 in cancer‐induced muscle wasting**

**Riccardo Ballarò** ^1,2^, Marc Beltrà^1,2^, Paola Costelli^1,2^, Hazel Szeto^3^ and Fabio Penna^1,2^

^1^ *Department of Clinical and Biological Sciences, Experimental Medicine and Clinical Pathology Unit, University of Turin, Italy;* ^2^ *Interuniversity Institute of Myology, Italy;* ^3^ *Mitochondrial Therapeutics Consulting, New York, NY, USA*

**Introduction:** Cancer cachexia is a syndrome characterized by muscle wasting that is enhanced by mitochondrial alterations.^1^ Indeed, oxidative capacity reduction and low intracellular ATP have been found in the skeletal muscle of cachectic animals.^2^ Among the different mitochondrial targeting compounds, the Szeto‐Schiller peptide (SS‐31) proved effective in preserving mitochondrial function by targeting cardiolipin, a phospholipid essential for the overall functioning of mitochondria.^3^ The present study aimed at evaluating the effects of SS‐31 on muscle wasting and mitochondrial alterations in tumour‐bearing mice.

**Methods:** Balb/c mice were divided in controls and C26 colon carcinoma bearers (C26). Both controls and C26 were treated with vehicle or SS‐31 (2 mg/kg). Mice were euthanized at 14 days after tumour transplantation.

**Results:** Animals with unrestricted tumour growth exhibited a reduction of muscle mass, muscle strength and food intake. At the molecular level, C26‐bearing (C26) mice showed impaired muscle oxygen consumption, which was associated with reduced levels of cardiolipin. Selectively targeting mitochondrial cardiolipin with SS‐31 counteracted body wasting, food intake loss and prevented the reduction of glycolytic fibre cross sectional area (CSA), while borderline significantly protected from muscle wasting. C26 mice exhibited a reduction of PGC‐1α, cytochrome c and SDH protein levels with no effect exerted by SS‐31 administration. On the contrary, in C26 mice, SS‐31 increased SDH activity and ATP levels. Mitochondrial alterations found in C26 mice also associated with a strong reduction in protein synthesis that was improved by SS‐31 treatment.

**Conclusions:** The present results suggest that targeting mitochondria and muscle function might be as important as targeting protein anabolism/catabolism in the prevention of cancer cachexia.

1. Argilés JM, Busquets S, Stemmler B, López‐Soriano FJ. Cancer cachexia: understanding the molecular basis. Nat Rev Cancer. Nature Publishing Group; 2014;14(11):754–62.

2. Pin F, Busquets S, Toledo M, Camperi A, Lopez‐Soriano FJ, Costelli P, et al. Combination of exercise training and erythropoietin prevents cancer‐induced muscle alterations. Oncotarget. 2015;6(41):43202–15.

3. Birk A V., Chao WM, Bracken C, Warren JD, Szeto HH. Targeting mitochondrial cardiolipin and the cytochrome c/cardiolipin complex to promote electron transport and optimize mitochondrial ATP synthesis. Br J Pharmacol. 2014;171(8):2017–28.

**2–04**

**IL‐4 counteracts cancer‐induced skeletal muscle atrophy: one cytokine for multiple effects**

**Domiziana Costamagna** ^1,2^, Robin Duelen^1^, Fabio Penna^3^, Paola Costelli^3^ and Maurilio Sampaolesi^1,2^

^1^ *Translational Cardiomyology, Stem Cell Biology and Embryology, Department of Development and Regeneration, University Hospital Gasthuisberg, Leuven, Belgium;* ^2^ *Division of Human Anatomy, Dept. of Public Health, Experimental and Forensic Medicine, University of Pavia, Italy;* ^3^ *Department of Clinical and Biological Sciences, University of Turin, Italy*

**Background and aims:** Terminal cancer patients frequently present with cancer cachexia, a syndrome characterized by involuntary skeletal muscle weight loss that interferes with antineoplastic therapies. Interestingly, cancer‐induced muscle protein depletion seems not only due to hyper‐activation of the main proteolytic systems in the muscle, but also to an impairment of satellite cell (SC) differentiation. Indeed, increased number of activated myogenic precursors, likely unable to complete the regeneration *in vivo*, has been reported in cachectic muscle.^1, 2^ Next to SCs, other cell populations are involved in myogenic differentiation, such as mesoangioblasts (MABs) and fibro‐adipogenic progenitors (FAPs). As for the former, their myogenic differentiation can be improved by inhibiting the BMP‐SMAD‐signalling.^3^ On the other side, FAP‐secreted cytokines, including IL‐4, enhance the differentiation rate of myogenic progenitors after injury and during dystrophic disease progression.^4, 5^ In this study, we investigated the role of interstitial cells in the skeletal muscle of mice implanted with the colon adenocarcinoma‐C26, focusing on the beneficial effect of IL‐4 in preventing detrimental FAP differentiation towards the adipocyte lineage.

**Results:** IL‐4 administration to C26‐bearing mice (C26 + IL‐4) partially rescued the reduction of body weight, muscle mass and myofiber cross sectional area (CSA) with respect to untreated C26 hosts. Such protective effect was associated with increased phosphorylation of molecules pertaining to anabolic pathways and with increased protein synthesis, evaluated as puromycin incorporation. Moreover, C26 + IL‐4 mice showed increased grip strength and running capacity during a single bout of exhausting exercise in comparison to untreated C26‐bearing mice. At the cellular level, IL‐4 was able to induce a decrease in FAP conversion to adipocyte.

**Conclusions:** On the whole, these results demonstrate that IL‐4 can partially rescue muscle atrophy in cachectic tumour‐bearing mice, probably due to anti‐inflammatory properties. Further experiments are needed to better clarify IL‐4 effects on muscle resident cell types different from FAPs.

**References:**

1. Penna F. *et al.,* PLoS ONE, e13604, 2010

2. He W. A. *et al*., J Clin Inv, 2013

3. Costamagna D. *et al*., JMCB, 2015

4. Joe A.W. *et al*., Nat Cell Biol, 2010

5. Mozzetta C. *et al*., EMBO Mol Med, 2013

**2–05**

**Lewis Lung carcinoma‐induced cachexia impairs insulin action on peripheral tissues**

**Xiuquing Han**, Steffen Henning Raun, Carlos Felipe Henriquez‐Olguín, Mona Sadek Ali, Lisbeth Liliendal Valbjorn Møller, Zhencheng Li, Jingwen Li, Thomas Elbenhardt Jensen, Erik Richter and Lykke Sylow

*Section of Molecular Phys Ther, Dept. of Nutrition, Exercise, and Sport, University of Copenhagen, Denmark*

**Introduction:** Cancer is often associated with insulin resistance and some studies report insulin resistance as a potential underlying cause of cancer‐associated cachexia. However, the studies are few and the specific tissues responsible for insulin resistance in cancer are unclear. We therefore analysed insulin action and glucose uptake in tumour‐bearing mice before and during development of cachexia.

**Methods:** C57BL/6 J mice were intraperitoneally inoculated with lewis lung carcinoma cells (2,5 * 105 cells per mice) and were studied for 15 days (pre‐cachexia) and 21–27 days (cachexia). Insulin action on blood glucose concentration and glucose uptake was measured by injecting insulin (0.3 U/kg body weight) together with 3H‐labelled 2‐deoxyglucose into the retro orbital vein for 10 minutes after which skeletal muscle and white adipose tissue (WAT) were excised.

**Results:** Pre‐cachexia tumour bearing mice (*n* = 13; average tumour volume of 500 mm3) exhibited a significant reduction in systemic insulin action. However, insulin‐stimulated glucose uptake in WAT and muscles was similar to non‐tumour bearing controls. Following cachexia, evident by a significant decrease in body weight (−3%) and WAT mass (−21%), tumour bearing mice (*n* = 7; tumour volume on average 1800 mm3) displayed reduced systemic insulin action and we observed a significant negative correlation between tumour size and insulin‐stimulated glucose uptake of both WAT and muscle.

**Conclusions:** Tumour bearing mice display whole body insulin resistance. However, only in cachexic mice was this accompanied by a reduction in insulin‐stimulated glucose uptake in white adipose tissue and muscle. Increasing insulin sensitivity in those tissues might provide better treatments to improve outcomes for cancer patients. Ongoing studies in our laboratory are now elucidating the molecular mechanisms underlying cancer‐induced insulin resistance in muscle and adipose tissue.

**2–06**

**Melanoma secreted factors in cancer‐associated cachexia**

**Ulf Gündisch**, Johanna Diener and Lukas Sommer

*University of Zurich, Institute of Anatomy*

Cancer‐associated cachexia is a severe disorder characterized by progressive loss of muscle mass with or without loss of adipose tissue. Cachexia is an often neglected condition even though it is estimated that almost half of all cancer patients develop cachexia syndrome at some point during disease progression. It has not only a dramatic impact on the patient's quality of life, but it is also associated with poor responses to treatment, hence decreasing the patient's chance of survival.

Several different pathways and multiple mechanisms have been reported to be involved in the development of Cachexia, however the role of tumour secreted factors in the establishment of this syndrome remain elusive. With this project, we want to decipher Melanoma secreted triggers that are responsible for the induction and maintenance of this disease.

First, we use the C2C12 immortalized mouse myoblast cell line to screen for cachectogenic melanoma cell lines *in vitro*. By co‐culturing C2C12 cells together with different melanoma cell lines, we will be able to assess whether myotube differentiation and myotube formation of C2C12 cells is impaired – an *in vitro* read out for cancer cachexia. The co‐culture growth medium of the selected melanoma cell lines will further be analysed via an unbiased mass spectrometry proteomic approach, to find candidate proteins secreted by cancer cells, which possibly induce cachexia‐like syndromes in the C2C12 cell line. To confirm the cachectogenic potential of specific cell lines, xenografts of patient‐derived melanoma cell lines will be performed in nude mice. The mice will be monitored for tumour growth, weight loss, food intake and changes in their body composition (lean, fat) via EchoMRI. By using CRISPR/CAS9 technology, we will generate knock outs of newly identified, potentially cachectogenic genes in human melanoma cell lines. Those cell lines will again be analysed in co‐culture experiments *in vitro* and with xenograph experiments *in vivo*.

Identification of new tumour‐secreted factors causing Cachexia will give valuable insights into how cancer cells communicate with their surrounding microenvironment and how they force the patient's body into a cachectic condition. These findings could point to new treatment strategies to prevent cancer‐induced weight loss and increase the overall survival of cancer patients suffering from cachexia.

**2–07**

**Disrupted regulation of skeletal muscle mTORC1 in tumour bearing mice**

**Brittany R. Counts**, Dennis K. Fix, Brandon N. VanderVeen, Ryan N. Montalvo and James A. Carson

*University of South Carolina, Columbia, USA*

Physical activity and feeding behaviours exert regulation on skeletal muscle anabolic signalling and are elevated during the dark cycle and negligible during the light cycle in mice. Mechanistic target of rapamycin complex 1 (mTORC1) axis serves to integrate muscle anabolism. Apc^Min/+^ (MIN) mouse is an established preclinical model of cancer‐induced cachexia. While cachexia suppresses basal mTORC1 signalling, significant gaps remain in understanding how the cancer environment effects anabolic inputs (feeding/activity) regulation on diurnal mTORC1 fluctuations.

**Purpose:** We examined cancers effect on the diurnal regulation of skeletal muscle mTORC1 signalling.

**Methods:** Male C57BL/6 and MIN mice had wheel access (B6: *N* = 17, Min: *N* = 19) for 2 weeks, while free living mice served as controls (FL) (B6: *N* = 24, Min: *N* = 22). Mice were sacrificed at the end of the light (SEDENTARY [SED]) or dark (ACTIVE [ACT]) cycles. To examine feeding, mice were sacrificed after a 12 hr fast or ad‐libitum food 1 hr into the dark cycle (FED) (B6: *N* = 7, Min: *N* = 8 per group). Gastrocnemius muscle was used for analysis and significance set at *p* ≤ 0.05.

**Results:** FL and wheel B6 mice exhibited significant differences in physical activity and food consumption between SED and ACT states; cancer environment disrupted this response. B6 wheel activity increased protein synthesis in the ACT state compared to FL B6 (*p* = 0.029) and induced 4EBP1 phosphorylation (*p* = 0.003), a marker of mTORC1 signalling. FL MIN had suppressed 4EBP1 phosphorylation in the ACT state (*p* = 0.051), however wheel activity did not alter this response (*p* = 0.831). B6 FED increased protein synthesis (*p* = 0.0001) and 4EBP1 phosphorylation (*p* = 0.013). Interestingly, MIN FED increased 4EBP1 phosphorylation (*p* < 0.0001) without altering protein synthesis (*p* = 0.129).

**Conclusions:** Skeletal muscle diurnal regulation is disrupted in MIN mice. Moreover, this suppressed anabolism may be driven by both reduced quantity of physical activity and an attenuated ability of physical activity and feeding to interact to regulate anabolic signalling.

*Supported by NCI R01‐CA121249*.

**2–08**

**Electrically stimulated acupuncture improve muscle function and increases renal blood flow through exosomes‐carried miR‐181**

**Manshu Yu**, Janet Klein and Xiaona Wang

*Emory University, Atlana, USA*

**Introduction:** our previous study found that acupuncture with low frequency electrical stimulation (Acu/LFES) can prevent muscle atrophy by increasing muscle protein anabolism in mouse models of chronic kidney disease, diabetes and denervation.

**Methods:** Mice were awake without any anaesthesia and appeared to be comfortable throughout the Acu/LFES. Acupuncture points selected were according to the WHO Standard Acupuncture guidelines. The mice were treated with Acu/LFES (anode: Yang Ling Quan, GB34 and cathode: Zu San Li, ST36) daily for 15 days. Renal plasma flow was measured by p‐Aminohippuric acid infusion. Muscle function was measured using a mouse grip strength meter. Exosomes were isolated by serial centrifugations. A miR deep sequencing assay and qPCR were used to identify microRNA expression in exosomes.

**Results:** Skeletal muscle function and protein anabolic markers were increased after Acu/LFES treatment. We found that Acu/LFES increases serum exosome abundance. Interestingly, we found that Acu/LFES treatment on the skeletal muscle enhanced renal blood flow. When exosome secretion was blocked using GW4869, the Acu/LFES‐induced increase in renal blood flow was limited. To identify how exosomes regulate renal blood flow, we performed microRNA deep sequencing in exosomes isolated from mouse serum and found that 34 microRNAs were altered by Acu/LFES. In particular, miR‐181d‐5p was increased in the serum exosomes of Acu/LFES treated mice. Using a luciferase reporter assay, we demonstrated that miR‐181 directly inhibits angiotensinogen, which provided potential evidence of Acu/LFES regulating renal blood flow.

**Conclusions:** Acu/LFES not only improves muscle function, but also increases miR‐181 in serum exosomes leading to increased renal blood flow. This study provides new insights about the mechanism(s) of muscle‐organ cross talk through exosome‐derived microRNA.

**Funding:** NIH R01 AR060268, AHA 17IBDG33780000.

**2–09**

**Antioxidant effects of bioactive ceramic water on muscle regeneration and vitamin C deficiency**

**Kyu‐Shik Jeong** ^1,7^, Mi‐Ran Ki^1^, Minhyung Kim^2^, Eun‐Joo Lee^1^, Kyung‐Jin Kim^3^, Kyung‐Ku Kang^1^, Eun‐Mi Lee^1^, Hyeon‐Dong Woo^4^, Daehee Hwang^2,4,6^, Yun‐Deuk Jang^4^ and SunYoung Park^1,5,7^

^1^ *Department of Veterinary Pathology, College of Veterinary Medicine, Kyungpook National University, Daegu, Republic of Korea;* ^2^ *School of Interdisciplinary Bioscience and Bioengineering, Pohang University of Science and Technology, Pohang, Republic of Korea;* ^3^ *School of life science and Biotechnology, Kyungpook National University, Daegu, Republic of Korea;* ^4^ *Department of Geology, School of life science and biotechnology, Kyungpook National University, Daegu, Republic of Korea;* ^5^ *Department of New Biology, Daegu Gyeonbuk Institute of Science and Technology, Daegu, Republic of Korea;* ^6^ *Center for Plant Aging Research, Daegu Gyeonbuk Institute of Science and Technology, Institute for Basic Science, Daegu, Republic of Korea;* ^7^ *Stem Cell Therapeutic Research Institute, Kyungpook National University, Daegu, Republic of Korea*

**Background:** Increased oxidative stress induces impairment of the antioxidant defence systems and defects in regeneration of injured tissues. We aim to evaluate the effects of antioxidant bioactive ceramic water on tissue repairing and anti‐aging.

**Methods:** In vitro, myogenic regeneration and antioxidant factors were determined in satellite cells cultured in media. We evaluated antioxidant effects and muscle regeneration effects of bioactive ceramic water in muscle lacerated model, SMP‐KO mice and MDX mice.

**Results:** Satellite cells grown in antioxidant bioactive ceramic water medium displayed increased expression of Notch1, Pax7, AMP‐activating protein kinase‐alpha (AMPK‐alpha) and anti‐oxidant enzymes and significant protection against hydrogen peroxide‐induced cell death. In vivo, antioxidant bioactive ceramic water enhanced muscle regeneration and reduced muscle fibrosis. The lifespan of SMP30 KO mice was extended by feeding. Moreover, the hepatic expression of AMPK‐α in antioxidant bioactive ceramic water‐fed SMP30 KO mice was comparable to that of VC‐fed SMP30 KO mice, whereas it was suppressed in tap water‐fed SMP30 KO mice. Long term supply of antioxidant bioactive ceramic water to muscular dystrophy mice decreased muscle injury and showed the muscle protective effect by reducing the oxidative stress.

**Conclusions:** These results suggest that antioxidant bioactive ceramic water improves antioxidant status through up‐regulating antioxidant enzymes and AMPK and thereby provides a favourable niche for satellite cells, leading to an increase in muscle regeneration and compensates oxidative stress.

**2–15**

**Aging associated reduction in skeletal muscle stem cell proliferation rate is accompanied by reduced focal adhesion formation, and increased yap signalling**

Mohammad Haroon^1^, Heleen E. Boers^1^, Willem M. Hoogaars^1^, Fabien LeG rand^2^, Lorenzo Giordani^2^, Louise Deldicque^3^, Katrien Koppo^4^, Astrid D. Bakker^5^, Jenneke Klein‐Nulend^5^ and **Richard T. Jaspers** ^1^

^1^ *Laboratory for Myology, Amsterdam Movement Sciences, Department of Human Movement Sciences, Vrije Universiteit Amsterdam, The Netherlands;* ^2^ *Center for Research in Myology, INSERM UMRS974, Sorbonne Universités, France;* ^3^ *Institute of Neuroscience, Université Catholique de Louvain, Louvain‐la‐Neuve, Belgium;* ^4^ *Department of Kinesiology, Exercise Physiology Research Group, KU, Leuven, Belgium;* ^5^ *Amsterdam Movement Sciences, Department of Oral Cell Biology, University of Amsterdam and Vrije Universiteit Amsterdam, The Netherlands*

**Introduction:** Decreased muscle stem cell (MuSC) number and impaired function may contribute to aging‐associated muscle wasting and impaired regeneration. Recent studies suggest that aged MuSCs have intrinsically impaired integrins/cytoskeleton, which may explain dysfunctional MuSC activation, growth and mechanosensitivity.^1^ Integrins, which anchor MuSCs to their niche components and regulate YAP^2^, decrease during aging. However, it is unknown whether YAP signalling is also altered in aged MuSCs. The aims of this study were to assess whether aging alters MuSC proliferation, integrin‐α7 expression, focal adhesion number, nuclear translocation of YAP and the response of MuSC to mechanical loading.

**Methods:** MuSCs were isolated from young (2 months) and aged (22 months) mouse (male, C57BL/6 J) hindlimb muscles, (*n* = 3). Cells were seeded on matrigel coated glass slides and subjected to pulsating fluid shear stress (PFSS) for 30 or 60 min with a peak shear stress rate of 6.1 Pa/s. After PFSS, cells (*n* = 50–90) from three independent experiments were stained for Integrin‐α7, phospho‐Paxillin and YAP using immunofluorescence and imaged using confocal microscopy.

**Results:** Aged MuSCs exhibited a lower proliferation rate, larger cell volume, and showed a reduced number of focal adhesions than young MuSCs as indicated by the expression of Integrin‐α7 and phospho‐Paxillin clusters (*p* < 0.05). Aged MuSCs were less firmly attached to the substrate, as a larger fraction detached in response to PFSS (p < 0.05). Despite a lower growth rate, YAP expression and its nuclearization was higher in aged MuSCs than in young MuSCs (p < 0.05). When subjected to PFSS, young and aged MuSCs showed a similar nitric oxide production.

**Conclusions:** Aging reduces the intrinsic capability of MuSCs to proliferate despite the high YAP signalling, possibly due to decreased integrin‐α7 expression and focal adhesion number, which is retained during culture *in vitro*. These results suggest that aging is associated with alterations in the mechanical properties of the MuSC cytoskeleton and niche.

1. Boers HE et al. J Orthop Res 2018

2. Heidary Arash et al. EMBO J 33(24):2997–3011, 2014.

**2–16**

**SIRT2 sustains muscle integrity via regulating anti‐adipogenic and pro‐myogenic process**

**SunYoung Park** ^1,2^, Soong‐Gu Ghim^1^, Ahmed K. Elfadl^1,2^, H.M. Arif Ullah^1,2^, Myung‐Jin Chung^1,2^, Ji‐Yoon Son^1,2^, Sul‐Gi Jeon^1^, JaeYeong Lee^1^ and Kyu‐Shik Jeong^1,2,3,4,5^

^1^ *Department of Veterinary Pathology, College of Veterinary Medicine, Kyungpook National University, Daegu, Republic of Korea;* ^2^ *Stem Cell Therapeutic Research Institute, Kyungpook National University, Daegu, Republic of Korea;* ^3^ *Cardiovascular Product Evaluation Center, Yonsei University College of Medicine, Seoul, Republic of Korea;* ^4^ *Cell Engineering for Origin Research Center, Seoul, Republic of Korea;* ^5^ *Department of Oral Pathology and Regenerative Medicine, School of Dentistry, Kyungpook National University, Daegu, Republic of Korea*

**Background:** Sirtuins (SIRT1–7), members of Histone deacetylases (HDACs) class III requiring NAD+ as a cofactor to deacetylate substrates, play critical roles in various biological processes including apoptosis, adipocyte differentiation and gluconeogenesis via regulating acetylation of histones and a number of transcriptional regulators. SIRT2 is known to participate in adipogenesis via regulating acetylation of FOXO1 which suppresses the transcriptional activity of PPARγ by enhanced binding with PPARγ.

**Methods:** The role of SIRT2 in aging process in terms musclular integrity regulating factor were determined by analysing SIRT2 deficient (SIRT2^−/−^) mice using tissue staining and molecular biological approaches.

**Results:** Aged SIRT2^−/−^ mice, up to 70 week‐old, showed increased body weight and visceral fat accumulation compared to WT. In addition, the amount of intramuscular adipocyte in skeletal muscle from aged SIRT2^−/−^ mouse was significantly increased and upregulated PPARγ and FOXO1 were observed suggesting activated adipogenesis. SIRT2 deficient skeletal muscle showed downregulated myogenic factors such as MyoD and myogenin, and reduced MYH2. These results indicate critical function of SIRT2 in the regulation of myogenic processes. In addition, increased IL‐6 level in SIRT2^−/−^ muscle suggests pathological feature of sarcopenia and addresses important role of SIRT2 in muscular integrity sustaining. Interestingly, the absence of SIRT2 altered other SIRTs expression level compared with WT control and the alteration was enhanced with aging.

**Conclusions:** In conclusion, SIRT2 sustains muscle function via anti‐adipogenic and pro‐myogenic effect, and abrogated SIRT2 function affects to aging of muscle by altering levels of other SIRTs.

**2–17**

**Insulin/IGF1 signalling is necessary for the growth‐promoting effects of β_2_‐adrenergic stimulation in mice**

Dawit A. Gonçalves^1,2,3^, Wilian A. Silveira^1^, Leandro H. Manfredi^1^, Flávia A. Graça^1^, Andrea Armani^3,4^, Enrico Bertaggia^3,4^, Brian T. O'Neill^5^, Juliano Machado^1^, Leonardo Nogara^3,4^, Marcelo G. Pereira^3^, Diletta Arcidiacono^6^, Stefano Realdon^6^, C. Ronald Kahn^5^, Marco Sandri^3,4,7^, Isis C. Kettelhut^2^ and **Luiz C. Navegantes** ^1^

^1^ *Departments of Physiology;* ^2^ *Biochemistry/Immunology, Ribeirão Preto Medical School/University of São Paulo, Brazil;* ^3^ *Department of Biomedical Sciences, University of Padova, Italy;* ^4^ *Venetian Institute of Molecular Medicine, Padova, Italy;* ^5^ *Joslin Diabetes Center, Harvard Medical School, Boston, USA;* ^6^ *Veneto Institute of Oncology IOV‐IRCCS, Padova, Italy;* ^7^ *Myology Center, University of Padova*

The stimulation of β_2_‐adrenoceptors can promote muscle hypertrophy and counteract atrophy and weakness, however, the underlying mechanisms involved in such effects remain elusive.

**Methods:** For the study of acute effects of the β_2_‐agonist formoterol on protein metabolism and signalling events, 2‐day fasted WT mice, muscle‐specific insulin receptor (IR) knockout (M‐IR−/−) mice and MKR mice, the latter expressing a dominant negative IGF‐1 receptor, which blocks both INS/IGF‐1 signalling, received one injection of formoterol (30, 300 or 3,000 ug·kg‐1; sc) or saline and skeletal muscles were analysed from 30 to 240 min. For the study of chronic effects of β_2_‐agonist on muscle plasticity and function and intracellular signalling pathways, fed WT and MKR mice were treated with formoterol (300 ug·kg‐1.day‐1) for 30 days.

**Results:** In fasted mice, one injection of formoterol inhibited proteasome activity and autophagosome formation that was paralleled by an increase in plasma insulin levels and Akt phosphorylation leading to suppression of the master regulators of atrophy, FoxOs, and the mRNA levels of their target genes. Although FOR effects on Akt/Foxo signalling were blunted in both M‐IR−/− and MKR mice, the inhibition of proteolysis markers by β_2_‐agonist was prevented only in MKR mice. Blockade of PI3K, Akt and mTORC1 also suppressed the acute β_2_‐agonist effects on proteolysis, FoxO1 and autophagy, respectively. Chronic stimulation of β_2_‐adrenoceptors in WT mice increased body and muscle growth and down‐regulated atrophy‐related genes, but these effects were abolished in MKR. However, increases in muscle force caused by β_2_‐agonist were only partially impaired in MKR, and formoterol‐induced slow‐to‐fast fibre type shift was not blocked at all in these mice.

**Conclusions:** Our data demonstrate that insulin/IGF1 signalling is necessary for the anti‐proteolytic and hypertrophic effects of β_2_‐adrenergic stimulation, but only partially contributes to strength gain and does not mediate fibre type transition.

*Financial Support: FAPESP, FAEPA/FMRP*.

**2–18**

**Potential involvement of Yes‐Associated Protein in anabolic resistance during mechanical loading in cachectic mice**

**Shuichi Sato** ^1,2^, Ian J. Pecor^1^, Emily R. Walker^1^ and Thao N. Nguyen^3^

^1^ *School of Kinesiology;* ^2^ *New Iberia Research Center;* ^3^ *Department of Nursing, University of Louisiana at Lafayette, Lafayette, LA, USA*

Cancer cachexia affects patients' survival, quality of life, and the response to chemotherapy. While muscle contraction/resistance exercise is a promising intervention to treat cachexia, a lower anabolic response in cachectic skeletal muscle has been reported and we have a limited understating of how this process happens in this condition. Yes‐associated protein (YAP) is a molecule involved in the Hippo signalling pathway and is known to regulate skeletal muscle fibre size and gene expressions in response to mechanical stimuli. However, the role of YAP on altered anabolic response in cancer cachexia is unknown. The purpose of this study was to determine whether altered YAP activation in response to mechanical overload is present in cachectic mice.

**Methods:** Male wild‐type (WT) and *Apc* ^*Min/+*^ (Min) mice were used in this study and they were subject to unilateral synergist ablation (SA) surgery at approximately 18 weeks of age. At 7 days following the SA surgery, both hypertrophied and contralateral control plantaris muscles were collected and used for further analysis. Either paired (control vs. SA‐exposed muscle) or unpaired t‐test (WT vs. Min) was used for statistical analysis.

**Results:** Prior to the SA surgery, Min mice had exhibited 10.2% ± 1.5 loss of body weights, confirming their cachectic condition. 7‐day mechanical overload increased plantaris weights in both mice, but the relative change in the muscle mass was smaller in Min mice than that of WT mice (24.9% ± 5.3 vs. 43.3% ± 5.2, respectively). Western blot analysis showed Min mice had a reduced activation of p70S6K (1.9‐fold vs. 3.4‐fold, respectively) and a smaller relative change of phosphorylated‐YAP1 levels compared to WT mice (15.7% ± 6.6 vs. 56.7% ± 7.8, respectively).

**Conclusions:** These data indicate that YAP plays a role in reduced response to mechanical stimulus in cachectic mice.

*Supported by Louisiana Board of Regents Support Fund (LEQSF(2017–20)‐RD‐A‐22) to SS*.

**2–19**

**Expression of genes and proteins in the skeletal muscle of individuals with cachexia: a systematic review**

**Cecily A. Byrne**, Amy T. McNeil, Amelia F. Brunskill, Timothy J. Koh and Giamila Fantuzzi

*Department of Kinesiology and Nutrition and University Library, University of Illinois at Chicago, Chicago, IL, USA*

**Introduction:** Sarcopenia is one of the central features of cachexia. Despite substantial evidence from animal and in vitro studies, the muscle‐intrinsic pathways associated with sarcopenia in individuals with cachexia remain poorly defined. This evidential weakness is in part due to the scattered nature of the literature, with most studies evaluating a single marker or just a few. We decided to address this gap by performing a systematic review aimed at collecting the evidence related to gene/protein expression in the skeletal muscle of individuals with cachexia and at assessing the strength of such evidence.

**Methods:** To this aim, we built a literature search using terms related to cachexia/sarcopenia, skeletal muscle and gene/protein expression to query the databases MEDLINE, CINHAL and EMBASE for relevant primary publications. We imposed no date restriction but limited the output to articles published in English. Our search generated 8570 references, 6639 of which were unique entries. Three researchers checked the title and abstract of references uploaded to the web application Rayyan QCRI to identify articles that warranted inclusion into full‐text analysis. Consensus settled conflicts. Inclusion criteria were: 1) Observational studies of individuals with disease‐associated sarcopenia; 2) Presence of a control group; 3) Presence of a biopsy of skeletal muscle; 4) Assessment of gene and/or protein expression in muscle. We identified 106 unique entries for full‐text analysis and are checking the reference list of these publications for potential additional relevant studies. We built a data extraction tool aimed at assessing: 1) The relevance of each study to the review's aim; 2) The study's findings directly related to the review's aim; 3) The strength of the study in terms of criteria for determination of cachexia/sarcopenia, criteria for matching cases with controls, determination of statistical power, and standards of quality for the molecular analysis.

**Results:** Data extraction and evaluation is in progress at the time of abstract submission.

**2–20**

**Depot specific adipose mRNA transcriptomics in cancer cachexia**

**Janice Miller** ^1^, Michael I. Ramage^1^, Stephen J. Wigmore^1^, James A. Ross^1^, Iain J. Gallagher^2^ and Richard J.E. Skipworth^2^

^1^ *Department of Clinical Surgery, University of Edinburgh, Royal Infirmary of Edinburgh;* ^2^ *Department of sport and exercise science, University of Stirling*

^†^Joint senior authors

**Introduction:** Skeletal muscle loss is a key marker in the diagnosis and classification of cachexia, however, the importance of fat wasting is less understood. During cachexia, different adipose depots around the body demonstrate differential wasting rates. Animal models suggest adipose tissue maybe a driver of muscle wasting through fat‐muscle crosstalk.

We aimed to perform global gene expression profiling of visceral (VAT) and subcutaneous (SAT) adipose from weight stable and cachectic cancer patients and healthy controls.

**Methods:** Cachexia was defined as >2% weight loss plus low CT‐muscularity. Biopsies of SAT and VAT were taken from patients undergoing resection for oesophago‐gastric cancer, and healthy controls (*n* = 24). RNA was isolated and reverse transcribed. cDNA was hybridized to the Affymetrix Clariom S Microarray. Data was examined using R/Bioconductor. Differential expression of genes was assessed using empirical Bayes and moderated‐t‐statistic approaches. Category enrichment analysis was used with a tissue specific background to examine the biological context of differentially expressed genes.

**Results:** Genes regulated in cancer VAT (regardless of weight loss) differed to controls by over 1200 genes. Over 2000 genes differed between cachexia VAT and SAT (*P* < 0.05). The gene showing the largest difference in expression was Intelectin‐1; a novel adipocytokine (*p* = 0.0001). Genes involving inflammation were upregulated in cachexia and control VAT. Energy metabolism (e.g. TCA) and fat browning genes were downregulated in cancer VAT (*p* = 0.0002). There was no difference in gene regulation between cachectic and weight stable cancer patients.

**Conclusions:** SAT and VAT have unique developmental gene expression signatures. VAT has large metabolic potential in cancer, and maybe a target for therapeutic manipulation. VAT may have a fundamental role in cachexia, but the downregulation of energy metabolism genes implies no role for browning in cachectic patients as suggested in pre‐clinical models. Validation of differentially regulated genes is ongoing and will be carried out by qRT‐PCR.

**2–21**

**Does miR‐675 control the sensitivity to inflammatory signalling in muscle to promote muscle wasting?**

Richard Paul, Mark Griffiths and **Paul Kemp**

*Section of Molecular Medicine, National Heart and Lung Institute, Imperial College London, UK*

**Introduction:** Muscle loss is a multifactorial process occurring in response to both chronic and acute conditions. We have shown miR‐675 is associated with muscle mass in COPD patients but the contribution of miR‐675 to acute muscle loss is unknown. Here we compare miR‐675 expression in the quadriceps of patients undergoing surgery to the gene expression profile.

**Methods:** Rectus femoris cross sectional area (RF_CSA_) was determined before and 7 days after surgery in 18 patients (9 wasting and 9 non‐wasting as defined by RF_CSA_ loss in 7 days of +/−10%) undergoing aortic surgery. Pre‐surgical and 24 h post‐surgical muscle biopsies were taken miRNA expression was quantified by qPCR and mRNA expression next generation sequencing.

**Results:** Using a nominal p value of *p* = 0.05, 3645 genes differed in expression between day 0 and 1.187 genes showed a nominal 2‐fold elevation whereas 27 showed a 2‐fold reduction. Gene set enrichment analysis showed positive enrichment for NF‐kB, STAT and c‐myc signalling. Weighted gene correlation network analysis showed that one day 1 module was associated with day 1 miR‐424 and 542‐3p but 2 modules associated with day 0 expression of miR‐675. Restriction of the gene set to those changed after surgery maintained the associations with miR‐675 but not miR‐424/542. Comparison of the correlation between miR‐675 and these genes showed that miR‐675 was positively associated with genes that increased in expression but negatively associated with those that reduced. These gene sets were enriched for inflammatory genes and myogenic genes respectively.

**Conclusions:** We propose that higher miR‐675 expression sensitizes individuals to inflammatory signals. This observation is consistent with studies showing that H19 the host gene for miR‐675 increases the expression of P65 and promotes JAK/STAT signalling. This increased sensitivity to inflammatory stimuli explains, in part, the association of miR‐675 with reduced muscle mass in COPD.

**2–22**

**miR‐542‐3p suppresses mitochondrial gene expression and promotes muscle wasting in patients undergoing aortic surgery**

Richard Paul, Mark Griffiths and **Paul Kemp**

*Section of Molecular Medicine, National Heart and Lung Institute, Imperial College London, UK*

**Introduction:** Mitochondrial dysfunction occurs in the muscle of patients with chronic diseases and is associated with muscle loss. Factors that regulate mitochondrial function/formation are likely to contribute to muscle loss. We have shown that miR‐542‐3p inhibits mitochondrial ribogenesis, promoting mitochondrial dysfunction and muscle loss in mice. We have also shown that it associates with muscle loss following surgery in humans. We examined miR‐542‐3p expression in the muscle of patients undergoing cardiac surgery and its association with gene expression.

**Methods:** Rectus femoris cross sectional area (RF_CSA_) was determined before and 7 days after aortic surgery in 18 patients (9 wasting and 9 non‐wasting; defined by RF_CSA_ loss in 7 days of +/−10%). An open muscle biopsy was taken after the onset of anaesthesia but prior to surgery and a closed muscle biopsy was taken 24 h later. Gene expression was quantified by next generation sequencing.

**Results:** Presurgical miR‐542‐3p was proportional to muscle loss in the 7 days following surgery. Weighted Gene correlation network analysis identified one module that was associated with RF_CSA_ loss (saddle brown module) which included the miR‐503 host gene (from the same locus as miR‐542‐3p). This module was also associated with the muscle expression of miR‐542‐3p. Gene set enrichment analysis (GSEA) of the genes in this module using module membership showed that the module was negatively enriched for mitochondrial components (NES = −3.20, fdr < 0.001). Similar analysis using correlation with miR‐542‐3p showed negative enrichment for oxidative phosphorylation (NES = −3.39, fdr < 0.001) and a positive enrichment for components of the P53 pathway (NES = 2.73, fdr < 0.001).

**Conclusions:** miR‐542‐3p is associated with reduced mitochondrial gene expression in patients undergoing surgery. As we have shown that miR‐542‐3p inhibits mitochondrial ribosome formation and mitochondrial ribosomal stress activates P53, we propose that miR‐542‐3p induced mitochondrial ribosome stress contributes to muscle wasting after surgery.

**2–23**

**Association between cachexia indicators and metabolic syndromes in hemodialysis patients**

**Tuyen Van Duong** ^1^, Te‐Chih Wong^2^, Hsi‐Hsien Chen^3,4^, Tso‐Hsiao Chen^4,5^, Yung‐Ho Hsu^4,6^, Sheng‐Jeng Peng^7^, Ko‐Lin Kuo^8^, Hsiang‐Chung Liu^9^, En‐Tsu Lin^10^ and Shwu‐Huey Yang^1,11,12^

^1^ *School of Nutrition and Health Sciences, Taipei Medical University, Taipei, Taiwan;* ^2^ *Department of Nutrition and Health Sciences, Chinese Culture University, Taipei, Taiwan;* ^3^ *Department of Nephrology, Taipei Medical University Hospital, Taipei, Taiwan;* ^4^ *School of Medicine, Taipei Medical University, Taipei, Taiwan;* ^5^ *Department of Nephrology, Wan Fang Hospital, Taipei, Taiwan;* ^6^ *Division of Nephrology, Department of Internal Medicine, Shuang Ho Hospital, Taipei, Taiwan;* ^7^ *Division of Nephrology, Cathay General Hospital, Taipei, Taiwan;* ^8^ *Division of Nephrology, Taipei Tzu‐Chi Hospital, Taipei, Taiwan;* ^9^ *Department of Nephrology, Wei Gong Memorial Hospital, Miaoli, Taiwan;* ^10^ *Department of Nephrology, Lotung Poh‐Ai Hospital, Yilan, Taiwan;* ^11^ *Research Center of Geriatric Nutrition, Taipei Medical University, Taipei, Taiwan;* ^12^ *Nutrition Research Center, Taipei Medical University Hospital, Taipei, Taiwan*

**Introduction:** Muscle wasting (cachexia) is common in hemodialysis patients and links to several adverse outcomes. We examined the association between cachexia indicators and metabolic syndrome (MetS) and its components.

**Methods:** A cross‐sectional study was conducted between September 2013 and April 2017 on 391 hemodialysis patients from seven hospital‐based dialysis centers. The dietary intake (3‐day dietary record), biochemical parameters (laboratory tests), body composition (bioimpedance analysis) was assessed. Cachexia indicators including BMI < 20 kg/m2, lowest tertile of handgrip strength, Anorexia (energy intake, EI < 20 kcal/kg/d), lowest quintile of appendicular skeletal muscle mass indexed to height, ASM/Ht2, high‐sensitivity C‐reactive protein, CRP > 0.5 mg/dL, Haemoglobin<12 g/dL, Albumin<3.2 g/dL. The MetS was diagnosed by the Harmonized Metabolic Syndrome criteria (HMetS). Logistic regression was utilized for association analysis.

**Results:** The mean of age, hemodialysis vintage, Charlson comorbidity index (CCI), physical activity were 60.8 ± 11.7 years, 5.7 ± 5.0 years, 4.7 ± 1.6, 4920.9 ± 1873.9 minute/week, respectively, 58.1% men, and 59.3% HMetS. In the multiple logistic regression, weight loss (BMI < 20 kg/m2) less likely to have elevated‐waist circumference, WC (Odd ratio, OR = 0.05, 95%CI, [0.01–0.16]), high Triglyceride (OR = 0.22[0.11–0.44]), low high‐density lipoprotein cholesterol, HDL‐C (OR = 0.37[0.21–0.65]) and HMetS (OR = 0.23[0.13–0.41]). Anorexia (EI < 20 kcal/kg/d) associated with impaired fasting glucose, IFG (OR = 2.17[1.11–4.25]), elevated‐WC (OR = 3.99[2.25–7.07]), high Triglyceride (OR = 2.67 [1.57–4.55]), low HDL‐C (OR = 3.65 [1.81–7.34]) and HMetS (OR = 4.31 [2.22–8.36]). Low ASM/Ht2 associated with elevated‐WC (OR = 0.16[0.09–0.29]), high SBP (OR = 0.45[0.26–0.78]), and HMetS (OR = 0.62[0.40–0.97]). Inflammation linked to elevated‐WC (OR = 2.23[1.33–3.72]), high Triglyceride (OR = 1.65[1.04–2.60]), and HMetS (OR = 1.81[1.11–2.94]). Anaemia associated with high systolic blood pressure (OR = 2.49[1.18–5.25]). Low serum albumin associated with higher IFG (OR = 5.32[1.17–24.17]), high diastolic blood pressure (OR = 11.29[4.34–29.37]), but lower elevated‐WC (OR = 0.27[0.08–0.96]). Handgrip did not show the significant association with MetS and its components (*p* > 0.05).

**Conclusions:** Cachexia indicators independently associated with HMetS and its components. Early identification of cachexia symptoms could contribute to MetS prevention and improve the dialysis outcomes.

**2–24**

**Identification of miRNAs in skeletal muscle associated with lung cancer cachexia**

**Wouter R.P.H. van de Worp** ^1^, Annemie M.W.J. Schols^1^, Annick Harel‐Bellan^2^, Marco C.J.M. Kelders^1^, Anne‐Marie C. Dingemans^1^, Jan Theys^3^, Ardy van Helvoort^1,4^ and Ramon C.J. Langen^1^

^1^ *Department of Respiratory Medicine, NUTRIM School of Nutrition and Translational Research in Metabolism, Maastricht University Medical Center+, Maastricht, the Netherlands;* ^2^ *Laboratory of Epigenetics and Cancer, Institut de Hautes Études Scientifiques, University of Paris‐Saclay, Paris, France;* ^3^ *Department of Radiation Oncology, GROW School for Oncology and Developmental Biology, Maastricht University Medical Center+, Maastricht, the Netherlands;* ^4^ *Nutricia Research, Utrecht, the Netherlands*

**Introduction:** Cachexia is highly prevalent in lung cancer and affects up to 60% of all lung cancer patients. MiRNAs have been reported to play fundamental roles in diverse biological and pathological processes, including multiple cancers and muscle development and regeneration. They have also been implicated in skeletal muscle atrophy, and a potential role remains to be explored.

**Methods and Results:** For this study miRNA expression analysis was performed using a Taqman Low‐Density Array in muscle biopsies of newly diagnosed non‐small cell lung cancer (NSCLC) patients with cachexia (*n* = 8) for comparison with gender‐ and age‐matched healthy control subjects (n = 8). 28 out of 754 miRNAs were differentially expressed in cachectic NSCLC patients compared to healthy controls, of which 5 miRNAs were significantly upregulated and 23 downregulated. Based on fold change (FC) and target prediction, the nine highest‐ranked miRNAs were selected for validation experiments by reverse transcription‐quantitative (qRT‐PCR) in a larger cohort consisting of healthy controls (*n* = 22) and treatment‐naïve NSCLC patients with (*n* = 15) and without (*n* = 11) cachexia. Compared to healthy controls, miR‐424‐5p (FC = 6; *p* < 0.01), miR‐424‐3p (FC = 15; *p* < 0.001) and miR‐450a‐5p (FC = 12; p < 0.01) were significantly upregulated, while miR‐451a (FC = 0.6; p < 0.001) and miR‐144‐5p (FC = 0.6; p < 0.01) were significantly downregulated in NSCLC patients with cachexia. In NSCLC patients without cachexia, expression levels of these miRNAs did not differ from healthy controls or NSCLC patients with cachexia. The expression levels of miR‐424‐5p (*r* = −0.38; p < 0.01), miR‐424‐3p (*r* = −0.50; p < 0.01) and miR‐450a (*r* = −0.37; *p* < 0.05) were inversely correlated and miR‐451a (*r* = 0.32; p < 0.05) positively correlated with hand grip strength.

**Conclusions:** We identified differentially expressed miRNAs putatively involved in lung cancer cachexia. These findings call for further studies aimed at investigating the causality of these miRNAs in muscle atrophy and the mechanisms underlying their differential expression in lung cancer cachexia.

**2–25**

**Gut microbiota composition and short‐chain fatty acid levels in human cancer cachexia: a pilot study**

**Jorne Ubachs** ^1,2^, Janine Ziemons^1^, Hans H. van Eijk^1^, Roy F.P.M. Kruitwagen^2^, Toon Van Gorp^2^, John Penders^3^, Steven W.M. Olde Damink^1^ and Sander S. Rensen^1^

^1^ *Department of Surgery, NUTRIM School of Nutrition and Translational Research in Metabolism, Maastricht University, Maastricht, the Netherlands;* ^2^ *Department of Gynaecology, GROW school for Oncology and Developmental Biology, Maastricht University, Maastricht, the Netherlands;* ^3^ *Department of Medical Microbiology, NUTRIM School of Nutrition and Translational Research in Metabolism, Maastricht University, Maastricht, The Netherlands*

**Introduction:** Cancer cachexia is a complex syndrome characterized by weight loss, inflammation, and disturbed energy metabolism. Recent animal studies suggest that the gut microbiota could be involved in the pathogenesis of cancer cachexia by modulating key metabolic pathways of the host through short‐chain fatty acids (SCFAs) production. We hypothesized that the relative abundance of bacteria known to be associated with deranged metabolism is different in cachectic patients, accompanied by reduced faecal SCFA levels.

**Materials & Methods:** Stool samples were collected preoperatively from patients suffering from breast cancer (*n* = 18), lung cancer (*n* = 16), or pancreatic cancer (*n* = 20). Cachexia was defined as weight loss >5% in the last six months. Relative abundance of *Akkermansia muciniphila*, Lactobacilli, and butyrate producers was quantified by qPCR. Faecal levels of the SCFAs acetate, propionate, and butyrate were assessed by liquid chromatography‐mass spectrometry.

**Results:** Prevalence of cancer cachexia was 17% (*n* = 3), 19% (n = 3) and 70% (*n* = 14) in breast, lung and pancreatic cancer patients, respectively (20 cachectic and 34 non‐cachectic patients, in total). The relative abundance of the indicated bacteria was not different between the different cancer types nor between cachectic and non‐cachectic individuals. Acetate, propionate, and butyrate levels were consistently lower in cachectic individuals, but the differences were not significant (acetate: 12.5 ± 3.4 vs. 14.3 ± 2.6 μmol/g, *p* = 0.51; propionate: 11.8 ± 2.6 vs. 29.2 ± 10.9 μmol/g, *p* = 0.46; butyrate: 25.7 ± 3.0 vs. 50.0 ± 15.8 μmol/g, *p* = 0.86). Faecal SCFA levels were associated with BMI as well as with abundance of Lactobacilli and *Akkermansia muciniphila*.

**Conclusions:** The current data suggest that gut microbiota composition and faecal SCFA levels are not affected in human cancer cachexia, although the number of samples was limited. Microbiota analyses by next‐generation sequencing and inclusion of more patients are ongoing. This will provide more extensive data and a more reliable data‐analysis.

**3–03**

**Preoperative CT assessed low skeletal muscle mass is a risk factor for postoperative complications in surgical oncology: a systematic review and meta‐analysis including over 14,000 patients**

**Sandra I. Bril** ^1,2^, Jeroen L.A. van Vugt^3^, Stefan Buettner^3^, Jan N.M. IJzermans^3^ and Remco de Bree^1^

^1^ *Department of Head and Neck Surgical Oncology, UMC Utrecht Cancer Center, University Medical Center Utrecht, Utrecht, The Netherlands;* ^2^ *Department of Medical Oncology, UMC Utrecht Cancer Center, University Medical Center Utrecht, Utrecht, The Netherlands;* ^3^ *Department of Surgery, Erasmus MC University Medical Centre, Rotterdam, The Netherlands*

**Introduction:** Radiologically determined preoperative low skeletal muscle mass (SMM), sometimes termed sarcopenia, has emerged as a novel biomarker for adverse outcomes in oncological surgery in the last decade. In a variety of tumour types, low SMM has been associated with postoperative morbidity and mortality. This systematic review and meta‐analysis aimed to investigate the impact of preoperative CT assessed low SMM on postoperative complications in patients undergoing surgery for malignant disease.

**Methods:** A systematic search of Embase, Google Scholar, PubMed and Web of Science was performed to identify relevant studies published until 17 July 2017. Screening for inclusion and checking the validity of included studies was carried out independently by two investigators (S.B. & J.v.V.) according to PRISMA guidelines. Outcomes extracted from the included studies were Clavien‐Dindo graded postoperative complications (grade ≥ 1 any complications; grade ≥ 3 major complications) in patients with and without low SMM. A random effects model was used to calculate the pooled odds ratio of low SMM for postoperative complications.

**Results:** In total, 87 studies (*n* = 19,986 patients) were included in this review, of which 60 studies (n = 14,720 patients) had sufficient data for inclusion in the meta‐analysis. Measurement methods and cut‐offs for low SMM varied significantly between studies. Low SMM incidence ranged from 7% to 79%. Preoperative low SMM was associated with an increased risk of any postoperative complications (OR 1.48; 95% CI 1.30–1.70, p < 0.001; I^2^ 44.3%) and major complications (OR 1.57; 95% CI 1.33–1.85; p < 0.001; I^2^ 37.6%). In the studies without sufficient data available for meta‐analysis, 16/27 studies found a significant association between low SMM and adverse postoperative outcomes, and 11/27 did not. None of the studies found a significant protective effect of low SMM.

**Conclusions:** Preoperative low SMM is a significant risk factor for postoperative complications in patients undergoing oncological surgery.

**3–04**

**Fat quality: the handsome stranger in esophageal cancer prognosis**

**Maëlle Anciaux** ^1^, Lieveke Ameye^2^, Thomas Guiot^3^, Patrick Flamen^3^, Serge Goldman^4^, Pieter Demetter^5^, André Van Gossum^6^, Marianne Paesmans^2^, Alain Hendlisz^7^ and Caroline Vandeputte^1^

^1^ *Gastro‐oncology lab, Jules Bordet Institute, Université Libre de Bruxelles, Brussels, Belgium;* ^2^ *Data Centre, Jules Bordet Institute, Université Libre de Bruxelles, Brussels, Belgium;* ^3^ *Department of Nuclear Medicine, Jules Bordet Institute, Université Libre de Bruxelles, Brussels, Belgium;* ^4^ *Department of Nuclear Medicine, Erasme Hospital, ULB, Brussels, Belgium;* ^5^ *Department of Pathology, Erasme Hospital, ULB, Brussels, Belgium;* ^6^ *Medico‐Surgical Department of Gastroenterology, Erasme Hospital, ULB, Brussels, Belgium;* ^7^ *Medical Oncology Department, Jules Bordet Institute, Université Libre de Bruxelles, Brussels, Belgium*

**Introduction:** Sarcopenia has been associated with poor survival in multiple cancers. Since dysphagia and weight loss are frequent in esophageal cancer (EC), this retrospective study aims to give global insight on the impact of body mass composition (BMC) on prognosis in EC.

**Methods:** CT‐based BMC was evaluated in 155 all‐stages EC patients at diagnosis. The index (area/height^2^) of skeletal muscle (SMI), subcutaneous (SFI) and visceral fat (VFI) were delineated on two adjacent slides at the third lumbar vertebra level by two independent investigators using PLANET ONCO® software (DOSIsoft, France). Sarcopenia was defined following the criteria of Prado *et al..*. (Lancet Oncol, 2008). Fat quality was indirectly measured by assessing the mean attenuation or density of each area. Survival and disease‐free survival (DFS) were calculated from date of baseline CT‐scan.

**Results:** Interobserver correlation was excellent for all BMC parameters measured (*r* = 0.94 to 0.99). We confirmed that patients with lower SMI than the sex‐specific median or with sarcopenia showed a poorer prognosis. Remarkably, high subcutaneous fat density (SFD), as well as high visceral fat density (VFD), also distinguished patients with poor 5‐year outcome. In contrast, low BMI and weight loss were not correlated with patient prognosis. Low C‐reactive protein (CRP) levels were only associated with better DFS. Detailed results are shown in Tables 1 and 2.

**Table 2:** 

**Parameters**	**Threshold**		**5‐year survival**	
	**male**	**female**	**p value**	**HR (95 CI)**
Low SMI	<51,00	<40,75	0,03	1,63 (1,04‐2,56)
Sarcopenia (Prado *et al.)*	SMI < 52,4	SMI < 38,5	0,01	1,771 (1,12‐2,79)
High SFD	> − 95,67	> − 99,39	0,0009	2,26 (1,47‐3,49)
High VFD	> − 95,63	> − 90,01	0,0071	1,8 (1,04‐3,10)
Low BMI	<20 if age < 70; <22 if age > 70		0,10	1,50 (0,86‐2,61)
High weight loss	>5% over past 6 months, or > 10% if greater than 6 months		0,11	1,84 (0,85‐3,96)

N.B. Thresholds for SMI, SFD and VFD are the sex‐specific medians.

**Table 3:** 

**Parameters**	**Treshold**	**5‐year DFS**	
		**p value**	**HR (95 CI)**
Low US‐CRP	2,65	0,049	0,55 (0,31‐1,00)

N.B. Threshold for US‐CRP is the median.

**Conclusions:** SFD and VFD appeared as robust prognostic factors in EC patients. Both parameters could potentially be used as prognostic factors. Among the anthropometric criteria evaluated, only SMI had an impact on prognosis, in contrast with BMI and weight loss. CRP, as an indicator of inflammation, was only associated with better DFS. These results confirm the validity of BMC assessment for evaluating patients prognosis.

**3–05**

**Adipose tissue browning in cachectic colorectal cancer patients**

**Ariene Murari Soares de Pinho** ^1,4^, Joyce de Cassia Rosa de Jesus Lima^1,4^, Nelson Inácio^2^, Raquel Galvão Figueredo^1^, Katrin Radloff^1^, Lila Missae Oyama^2^, Flávio Tokeshi^3^, Paulo Sérgio Martins de Alcantara^3^, Marília Seelaender^1,4^ and José Pinhata Otoch^1,3,4^

^1^ *Cancer Metabolism Research Group, University of São Paulo;* ^2^ *Federal University of São Paulo;* ^3^ *Department of Clinical Surgery, University Hospital, University of São Paulo;* ^4^ *Faculdade de Medicina, University of São Paulo*

**Background:** Cancer Cachexia is a multifactorial syndrome characterized by poor quality of life, weight loss, anorexia, fatigue, asthenia, anaemia, insulin resistance and systemic inflammation. In recent years, adipose tissue browning has been proposed to contribute to cachexia. This process is related with increased thermogenic activity of the white adipose tissue, causing enhanced basal metabolic rate and higher energy consumption, thereby aggravating weight loss. Atrial natriuretic peptide (ANP) can promote browning, stimulate lipolysis and augmente energy expenditure.

**Aim:** To examine circulating ANP and browning‐associated genes involved in browning in cachectic and non‐cachectic cancer patients.

**Methods:** Colorectal cancer patients were divided into weight‐stable cancer (WSC, *n* = 20), and cachectic cancer (CC, *n* = 30). Blood sampling and adipose tissue collection was performed after signing the informed consent form, during surgery. ANP was quantified with Luminex®xMAP technology and the expression of SREBP1c and TFAM– transcription factors involved in lipolysis and mitochondrial biogenesis was analysed by RT‐PCR.

**Results:** Plasma concentrations of NT‐ProANP were higher in CC, compared to WSC (WSC: 526 ± 75.3; CC: 859 ± 58.86, *p* = 0.0012). In CC SREBP1c expression was 5.3 fold higher than in WSC (WSC: 8.90 ± 2.73; CC: 47.50 ± 11.61, *p* = 0.0051) and TFAM was upregulated about 2.5 fold (WSC: 0.81 ± 0.19; CC: 2.02 ± 0.47, *p* = 0.037).

**Conclusions:** The results show that circulating ANP concentration and augmented browning‐associated gene expression are altered in cachectic patients, yet it is not clear whether this represents an effect or a cause to the establishment of cachexia‐related alterations.

**3–06**

**Low bone mass is associated with cachexia in advanced cancer patients**

**Robert D. Kilgour** ^1,2^, Leonard Rosenthall^3^, Popi Kasvis^1^ and Antonio Vigano^1^

^1^ *McGill Nutrition and Performance Laboratory, Montreal, Canada;* ^2^ *Department of Health, Kinesiology and Applied Physiology, Concordia University, Montreal, Canada;* ^3^ *Department of Radiology, McGill University Health Centre, Montreal, Canada*

**Introduction:** Very little is known about the incidence of osteopenia and osteoporosis in cancer patients with cachexia. This prospective study was designed to compare bone mineral density (BMD) of the femoral neck (FN) and total hip (TH) among cachectic and non‐cachectic cancer patients and to examine associations between bone and skeletal muscle.

**Methods and Procedures:** A consecutive sample (*n* = 188) of cancer patients enrolled in the Cancer Rehabilitation Program of the McGill University Health Centre, were classified into cachexia (C; *n* = 56), supportive (S; *n* = 88, patients with active tumours and on cancer treatment) and restorative (R; *n* = 44, patients with no tumour and off treatment) groups. Appendicular skeletal muscle mass index (ASMI) and BMD of the FN and TH were measured using dual energy x‐ray absorptiometry (DXA). Comparisons were made among the 3 groups with respect to FN and TH t‐scores, the incidence of low bone mass (osteoporosis and osteopenia) and its relationship with appendicular muscle mass.

**Results:** The C group has significantly lower ASMI than the S group. FN and TH t‐scores were significantly lower in the C group when compared to the S group (FN: C vs S, −1.44 ± 1.04 vs −1.06 ± 0.95; *p* = 0.036; TH: C vs S ‐1.04 ± 1.08 vs −0.56 ± 1.07; *p* = 0.015). The R group had significantly lower TH t‐scores than the S group. Those patients with poor ASMI values had low FN and TH t‐scores. Each group had similar incidences of osteoporosis (C, 15.7%; S, 8.5%; R, 12.8%). Low bone mass (osteoporosis + osteopenia) was prevalent in all three groups (C, 66.6%; S, 53.6%; R, 66.6%).

**Conclusions:** Cancer patients who are cachectic or in remission are at particular risk of osteopenia and osteoporosis. Exercise and nutritional interventions to improve BMD should be integrated within cancer rehabilitation programs.

**3–07**

**Cancer‐associated cachexia generates disruption of skeletal muscle mitochondria and increase in autophagy proteins in patients with gastrointestinal cancer**

**Gabriela S. de Castro** ^1^, Estefania Simões^1^, Joanna D.C.C. Lima^1^, Milene Ortis‐Silva^2^, William T. Festuccia^2^, Dario Coletti^3^, Flávio Tokeshi^4^, Paulo S. Alcantara^4^, José P. Otoch^4,5^ and Marilia Seelaender^1,5^

^1^ *Cancer Metabolism Research Group, Department of Cell and Tissue Biology, Institute of Biomedical Sciences, University of São Paulo, Brazil;* ^2^ *Department of Physiology & Biophysics, Institute of Biomedical Sciences, University of São Paulo;* ^3^ *Department of Biological Adaptation and Aging B2A, Pierre et Marie Curie University (Paris 6), France;* ^4^ *Department of Clinical Surgery, University Hospital, University of São Paulo, Brazil;* ^5^ *Faculty of Medicine, University of São Paulo, Brazil*

**Introduction:** Cachexia is a wasting syndrome defined by a continuous loss of skeletal muscle mass due to an imbalance between protein synthesis and degradation with or without body fat loss. Muscle is one of the most affected organs and loss of skeletal muscle is related to poor prognosis. Dysfunctional mitochondria have been related to lower muscle strength and quality, as well as to increased atrophy. Therefore, we aimed at evaluating skeletal muscle mitochondria morphology and autophagy markers in patients with cancer‐associated cachexia.

**Methods:** Patients with gastrointestinal cancer were recruited after signature of the informed consent form. *Rectus abdominis* muscle biopsies were collected during surgery for tumour resection. Patients were separated into Weight‐Stable Cancer (WSC) and Cachectic Cancer (CC) groups (WSC n = 3–13 and CC n = 4–16) according to criteria described by Evans et al (2008). Muscle mRNA was extracted and real time PCR gene expression was analysed using 2^‐ΔΔCT^. Autophagy protein markers were quantified by Western blot. Transmission electron microscopy was used to acquire images of intermyofibrillar mitochondria.

**Results:** CC patients presented higher weight loss (p = 0.001) and serum C reactive protein (*p* = 0.004) and lower haemoglobin (p = 0.001) compared to WSC patients. Electron microscopy analysis revealed an increase in the intermyofibrillar mitochondrial area (p = 0.01) in the CC group. Relative mRNA of fission 1 and mitofusin 2 were increased in the CC group (*p* = 0.04 for both). LC3 II and autophagy proteins (ATG) 5 and 7 were also increased in the CC group (*p* = 0.042 and *p* = 0.02, respectively).

**Conclusions:** Disruption in the skeletal muscle mitochondria and increase in autophagy may be related to muscle atrophy in CC patients. Our results indicate that cancer‐associated cachexia leads to an exacerbated muscle‐stress response to chronic inflammation that may culminate in muscle loss.

**3–09**

**Comparison of different scales of estimation of muscle wasting in Mexican patients with prostate cancer associated with cachexia**

**Alan Espinosa Marrón** ^1^, María del Pilar Milke García^1^, Aquiles Rubio Blancas^1^, Ana Paula Bravo García^1^, Itzel Salcedo Grajales^1^, Anaís Camacho Zamora^1^, María Teresa Bourlon de los Ríos^2^, Ulices Alvirde García^3^, Ricardo Alonso Castillejos Molina^4^ and Christian Aníbal Quiñones Capistrán^4^

^1^ *Nutrition Division, Instituto Nacional de Ciencias Médicas y Nutrición Salvador Zubirán;* ^2^ *Oncology Department, Instituto Nacional de Ciencias Médicas y Nutrición Salvador Zubirán;* ^3^ *Fundación Clínica Médica Sur;* ^4^ *Urology Department, Instituto Nacional de Ciencias Médicas y Nutrición Salvador Zubirán, Mexico City, Mexico*

**Introduction:** Prostate cancer (PC) is the second most diagnosed, the fifth cause of cancer mortality in men worldwide and the first cause in Mexico. One of its consequences is cachexia, characterized by muscle mass wasting. It is relevant to compare different methods for the diagnosis of muscle mass wasting and functionality in a Latin American population.

**Methods:** Thirty Mexican patients (67.6 ± 8.1 years) with previous diagnosis of PC were analysed cross‐sectionally. Muscle wasting was measured through various techniques and compared: body mass index (BMI) (<22 kg/m^2^), calf circumference (CC) (<29 cm), arm muscle area (AMA) (≤ 15th percentile), “Get up and go test” (<12.47 s), dynamometry (<30 kg), and bioelectrical impedance vector analysis (BIVA), the latter considered as the reference standard. A Spearman's rank‐order correlation, a logistic regression analysis to identify risk factors for cachexia development and central tendency tests for comparison of variables were performed.

**Results:** Cachexia was diagnosed by BIVA in half of the patients (Figure 1) who also presented a significant decrease in AMA and a poor performance in the Get up and go test (*p* = 0.049 and 0.005, respectively). A correlation between the diagnosis of cachexia by BIVA was only found with dynamometry (*p* = 0.012) (Table 1). In addition, a dynamometry <30 kg was identified as a risk factor for the development of cachexia (OR [CI]: 12.2 [1.2–118.3], *p* = 0.03).

**Conclusions:** Muscle wasting and poor physical performance is common in patients with PC. Arm dynamometry is comparable to BIVA; however, its advantages in clinical application could make it the preferred diagnostic method.

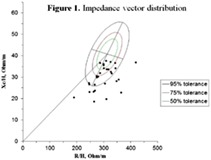

**Table 4:** Correlation of the different techniques for measuring muscle mass and performance in patients diagnosed with cachexia by BIVA.

	**Diagnosis of cachexia by BIVA**	
*Muscle wasting diagnosis techniques*	*Rho*	*p value*
BMI (<22 kg/m^2^)	0.08	0.66
CC (<29 cm)	0	1
AMA (≤ percentile 15)	0.33	0.07
Get up and go (<12.47 s)	0.33	0.07
Dynamometry (<30 kg)	0.45	0.012*
Serum albumin (<3.2 g/dl)	0.18	0.32

Statistical tests: Spearman's rank‐order correlation

* Statistically significant difference.

Abbreviations: BIVA: bioelectrical impedance vector analysis; Rho: Spearman's rank‐order correlation; BMI: body mass index; CC: calf circumference; AMA: arm muscle area.

**3–10**

**Skeletal muscle mass/density and recovery of physical functioning and fatigue after surgery in early‐stage colorectal cancer patients**

**Harm van Baar** ^1^, Sandra Beijer^2^, Ellen Kampman^1^ and Renate M. Winkels^1,3^

^1^ *Division of Human Nutrition, Wageningen University & Research Wageningen, The Netherlands;* ^2^ *Netherlands Comprehensive Cancer Organization (IKNL), Utrecht, The Netherlands;* ^3^ *Department of Public Health Sciences, Penn State College of Medicine, Hershey, PA, USA*

**Introduction:** Associations between low skeletal muscle mass and both physical functioning (PF) and fatigue have been reported in various studies, while studies on the association between low radiographic muscle density, PF and fatigue are lacking. Furthermore, it is not known whether low muscle mass and/or density are associated with poor recovery of PF and fatigue after surgery. We hypothesized that low skeletal muscle mass and density are associated with worse recovery of PF after surgery and higher fatigue levels in early‐stage colorectal cancer (CRC) patients.

**Methods:** Within a prospective cohort of *n* = 604 early‐stage CRC patients, we collected information on PF and fatigue using the EORTC QLQ‐C30 questionnaire shortly after diagnosis and 6 months later. Skeletal muscle mass and ‐density were assessed using pre‐operative computed tomography (CT) images. Low muscle mass and ‐density were identified using previously determined cut‐off values. Cox regression models were used to calculate prevalence ratios (PRs). All PRs were adjusted for age, sex, stage of disease and total adipose tissue.

**Results**: The average age of the study population was 65.7 ± 9.0, 37% were women and 67% of the patients had a tumour located in the colon. Low muscle mass was present in 26% of the patients and low muscle density in 25%. Fifty percent of the patients did not recover to preoperative PF and 48% reported higher fatigue scores at six months. Both low muscle mass and ‐density were not significantly associated with recovery of PA and fatigue after surgery.

**Conclusions:** Low muscle mass and density were not associated with recovery of PF and fatigue six months after diagnosis in early‐stage CRC patients. It should be investigated if post‐diagnosis improvement of muscle mass and/or ‐density can improve post‐surgery recovery of PA and fatigue.

**3–11**

**What affects radiographic muscle density? Associations between lifestyle, weight loss and muscle density**

**Harm van Baar** ^1^, Sandra Beijer^2^, Ellen Kampman^1^ and Renate M. Winkels^1,3^

^1^ *Division of Human Nutrition, Wageningen University & Research Wageningen, The Netherlands;* ^2^ *Netherlands Comprehensive Cancer Organization (IKNL), Utrecht, The Netherlands;* ^3^ *Department of Public Health Sciences, Penn State College of Medicine, Hershey, PA, USA*

**Introduction:** Low radiographic muscle density is associated with higher mortality in cancer patients. Previous studies reported an association between higher total adiposity, increasing age and pre‐existing comorbidities and lower muscle density. Whether lifestyle and/or weight loss affect muscle density has not been studied yet. We assessed whether lifestyle factors and pre‐diagnosis weight loss were associated with skeletal muscle density.

**Methods:** Within a prospective observational study, *n* = 709 stage I‐III colorectal cancer (CRC) completed questionnaires on dietary intake (energy, protein, fat and carbohydrates), physical activity and smoking shortly after diagnosis. Body weight two years prior to diagnosis was recalled at diagnosis. Muscle density was assessed using computed tomography (CT) images at diagnosis and low muscle density was identified using previously determined cut‐off values. Cox regression models, with a fixed time variable, were used to calculate prevalence ratios (PRs). All PRs were adjusted for age, gender and total adipose tissue.

**Results:** The average age of the study population was 66.3 ± 9.1 years, 38% were women, and low muscle density was present in 28% of the patients. Current smoking (PR 1.78, 95% CI: 1.02–3.09) and pre‐diagnosis weight loss >5% (PR 1.75 95% CI: 1.27–2.41) were significantly associated with increased risk of low muscle density, while moderate to vigorous physical activity (hours per week) (PR 0.98, 95% CI: 0.97–0.999) was significantly associated with decreased risk of low muscle density. No association between low muscle density and energy and macronutrient intake was found.

**Conclusions:** In stage I‐III colorectal cancer patients, low muscle density was associated with higher prevalence of smoking, and with pre‐diagnosis weight loss, while higher levels of moderate to vigorous physical activity was associated with a lower risk of low muscle density. Prospective studies are needed to confirm these findings, which may aid to inform targeted intervention studies to improve muscle density.

**3–12**

**The impact of sarcopenia and frailty phenotype on health related quality of life of colorectal cancer patients**

Mariana Vieira Barbosa^1^, Mylena Pinto dos Santos^2^ and **Renata Brum Martucci** ^1,2^

^1^ *Nutrition Institute, Rio de Janeiro State University, Rio de Janeiro, Brazil;* ^2^ *Nutrition and Dietetic Service, National Cancer Institute, Rio de Janeiro, Brazil*

**Introduction:** Sarcopenia and frailty are overlapping syndromes, both are associated with aging and disease, increasing the risk of adverse events such as physical disability, reduction in health related quality of life (HRQL) and death. The aim of the study was to evaluate the influence of sarcopenia and frailty phenotype on HRQL of colorectal cancer patients.

**Methods:** Cross sectional study including adult (≥20y) colorectal cancer patients. Clinical and nutritional data were collected. Sarcopenia was defined as low skeletal muscle mass < 6.76 kg/m^2^ for women and < 10.76 kg/m^2^ for male patients assessed by bioelectrical impedance analysis (Janssen et al..., 2000). Frailty was defined by Fried et al (2001) as the presence of 3 or more of the following criteria: unintentional weight loss (>3 kg in past year), self‐reported exhaustion, weakness (low handgrip strength), slow walking speed (gait speed) and low physical activity (IPAQ questionnaire). HRQL was assessed using the EORTC QLQ‐C30 questionnaire.

**Results:** 73 patients (age 63.9 ± 11.3 y) were included; 63% women; 73.7% well‐nourished by the PG‐SGA; 46.8% were sarcopenic and 19.2% frail. There was no difference in HRQL when compared sarcopenic patients with non‐sarcopenic patients, however, the frail patients presented worse HRQL when compared to non‐frail patients in Global Health Status (64.9 ± 27 vs 86.8 ± 13.7; *p* < 0.01); Role Function (46.4 ± 38.2 vs 92.1 ± 14.3; *p* < 0.001); Physical Function (55.7 ± 29.4 vs 90.2 ± 14.3; p < 0.001); Fatigue (43.6 ± 32.4 vs 9.4 ± 14.0; *p* = 0.001); nausea/vomiting (11.9 ± 19 vs 0.9 ± 3.8; *p* = 0.002) and Appetite lost (35.7 ± 42.3 vs 0.0 ± 0.0; p = 0.002). The regression model adjusted by age, sex, BMI, cancer stage and sarcopenia showed that be frail was associated with worse Global Health Status (β:‐17.8; 95%CI ‐32.3/−3.3); Role Function (β:‐34.6; 95%CI ‐52.4/−16.8); Physical Function (β:‐22,6; 95%CI ‐36,9/−8,3) and Fatigue (β:24,9; 95%CI 10,2/39,6).

**Conclusions:** Although, Sarcopenia was not associated with HRQL, Frailty was an independent predictor of worse HRQL in colorectal cancer patients.

**3–13**

**Characterization of the intestinal smooth musculature of pancreatic cancer patients with sarcopenia**

**Rianne D.W. Vaes** ^1^, Tessa T.J. Welbers^1^, David P.J. van Dijk^1^, Dorit Rennspiess^2^, Axel zur Hausen^2^, Steven W.M. Olde Damink^1^ and Sander S. Rensen^1^

^1^ *Department of Surgery and NUTRIM School of Nutrition and Translational Research in Metabolism, Maastricht University, Maastricht, The Netherlands;* ^2^ *Department of Pathology and GROW School for Oncology and Developmental Biology, Maastricht University Medical Center+ (MUMC+), Maastricht, The Netherlands*

**Background:** Loss of skeletal muscle tissue is one of the defining characteristics of cancer cachexia. Since cachectic cancer patients often report gastrointestinal motility problems, a process intricately linked to proper intestinal smooth muscle function, we hypothesized that the intestinal smooth musculature could also be affected in cachexia.

**Methods:** Archived full‐thickness jejunal tissue sections from 57 pancreatic cancer patients were analysed by picrosirius red stains for collagen and by immunohistochemistry for contractile smooth muscle markers α‐smooth muscle actin (α‐SMA) and smoothelin, and CD117, a marker of the interstitial cells of Cajal which regulate intestinal contraction. Muscle wall thickness, marker staining intensities and distributions, and collagen deposition (as indication of smooth muscle dedifferentiation) were quantified by morphometry using Pannoramic Viewer software. Patients were assigned to a sarcopenia or non‐sarcopenia group based on their skeletal muscle index (SMI) as assessed by analyses of a single computed tomography slide at the L3 level, using validated sex‐specific cut‐offs (Martin et al.).

**Results:** Mean age and BMI of both groups were similar. Intestinal smooth muscle wall thickness did not differ between the sarcopenia and non‐sarcopenia group (1661 ± 125.0 vs. 1439 ± 93.5 μm, *p* = 0.41). Whereas α‐SMA staining intensity was similar between the groups, staining intensity of smoothelin, a marker of the advanced contractile phenotype, was reduced (143.0 ± 4.9 vs. 125.4 ± 5.5 arbitrary units, *p* = 0.02) in sarcopenic patients. The distribution of CD117^+^ interstitial cells of Cajal was similar in both groups, but pronounced collagen deposition around the myenteric plexus was more often observed in patients with sarcopenia (*p* = 0.04).

**Conclusions:** These data suggest that cancer cachexia is not only associated with skeletal muscle wasting, but also affects the intestinal smooth musculature. The reduced contractile smooth muscle marker expression and collagen accumulation around the myenteric plexus indicate that both contractile function of smooth muscle cells and regulation of their contractile functionality could be compromised.

**3–14**

**Immunometabolism in cancer cachexia**

**Rianne D.W. Vaes** ^1^, Ryan Sambo^1^, Marike W. van Gisbergen^3^, Ludwig J. Dubois^3^, Steven W.M. Olde Damink^1,2^ and Sander S. Rensen^1^

^1^ *Department of Surgery, NUTRIM School of Nutrition and Translational Research in Metabolism, Maastricht University, Maastricht, the Netherlands;* ^2^ *Department of Surgery, Maastricht University Medical Centre+, Maastricht, The Netherlands;* ^3^ *Department of Radiotherapy, The M‐lab group, GROW ‐ School for Oncology and Developmental Biology, MCC ‐ Maastricht Comprehensive Cancer Centre, Maastricht University Medical Centre+, Maastricht, The Netherlands*

**Introduction:** The majority of pancreatic cancer patients develop cachexia during their disease course. Systemic inflammation is an important factor in the pathophysiology of cancer cachexia. Since inflammation has been shown to be associated with significant changes in leukocyte metabolism in the context of other disorders, we hypothesized that tumour‐derived factors from cachectic pancreatic cancer patients would induce a pro‐inflammatory response in macrophages accompanied by a shift towards glycolysis to meet their metabolic demand.

**Methods:** THP‐1 monocytes were differentiated into macrophages by treatment with 25 ng/mL phorbol 12‐myristate 13‐acetate for 48 hours and subsequently exposed to conditioned medium (CM) from independent pancreatic tumour organoid lines (CL03, CL06, CL09, and CL11) established from cachectic pancreatic cancer patients for 24 h. mRNA expression levels of pro‐inflammatory cytokines (*IL‐1*, *IL‐6*, and *IL‐8*) and key glycolytic genes (*SLC2A1*, *HK2*) were determined by qPCR. The metabolic profile of CM‐treated THP‐1 macrophages was assessed using the Seahorse XF96 extracellular flux analyser.

**Results:** Expression of pro‐inflammatory cytokines (*IL‐1, IL‐6, IL‐8*) and key glycolytic genes (*SLC2A1, HK2*) was not induced in macrophages after incubation with tumour organoid‐CM. In line with this, the glycolytic capacity of THP‐1 macrophages incubated with tumour organoid‐CM was similar compared to the glycolytic capacity of macrophages treated with control medium. However, CM of two out of four tumour organoid lines induced a markedly increased basal oxygen consumption rate (CL03: 67.5 ± 1.9 pmol/min; *p* = 0.002, CL06: 66.6 ± 4.9 pmol/min; *p* = 0.021, CL09: 57.4 ± 3.9 pmol/min; *p* = 0.150, and CL11: 41.8 ± 5.3; *p* = 1.00 versus 36.8 ± 4.0 pmol/min in macrophages treated with control medium).

**Conclusions:** The tumour secretome from cachectic pancreatic cancer patients does not induce a pro‐inflammatory response or glycolysis in macrophages, but increases basal macrophage oxygen consumption rates to various extents. This might reflect a shift towards the alternatively activated M2 phenotype.

**3–15**

**Thoracic muscle radiation attenuation for the prediction of postoperative pneumonia following partial hepatectomy for colorectal metastasis**

**Gregory van der Kroft** ^1^, David P.J. van Dijk^2^, Sander S. Rensen^2^, Bianca de Greef^3^, Malcolm A. West^4^, Kris Ostridge^5^, Ulf P. Neumann^1,2^ and Steven W.M. Olde Damink^1,2^

^1^ *Department of General, Gastrointestinal, Hepatobiliary and Transplant Surgery, Uniklinikum RWTH‐Aachen, Aachen Germany, European Surgical Center Aachen Maastricht (ESCAM);* ^2^ *Department of Surgery, Maastricht University Medical Centre, Department of Surgery, European Surgical Center Aachen Maastricht (ESCAM), NUTRIM School of Nutrition and Translational Research in Metabolism, Maastricht University, Maastricht, the Netherlands;* ^3^ *Department of Clinical Epidemiology & Medical Technology Assessment, Maastricht University Medical Centre, Maastricht, The Netherlands;* ^4^ *Academic Unit of Cancer Sciences, Faculty of Medicine, University of Southampton, Southampton, UK;* ^5^ *Southampton NIHR Respiratory Biomedical Research Unit, Southampton, UK*

**Background:** Low muscle radiation attenuation is indicative of myosteatosis and predictive of poor outcome following oncological surgery in some cancer types. Postoperative pneumonia has been shown to increase postoperative morbidity, prolong hospital admission, and increase in‐hospital mortality following a range of surgical interventions. Patients undergoing partial hepatectomy develop reactive pleural effusion which increases the risk of post‐operative pneumonia to incidences above 10%. We hypothesized that low muscle radiation attenuation at the 4th thoracic vertebrae (T4 MRA) is predictive for postoperative pneumonia following liver surgery.

**Methods:** Prospective infection control data of 180 patients who underwent elective colorectal liver metastasis resection were used to identify postoperative pneumonia. Body composition was assessed using CT‐scans at the 3^rd^ lumbar vertebrae (L3) and 4^th^ thoracic vertebrae (T4) to determine: (i) muscle radiation attenuation (MRA); (ii) total skeletal muscle area (SMA); (iii) and muscle index. Sex specific cut‐offs were determined at the lower tertile. Body composition variables were corrected for known confounders and related to postoperative pneumonia by multivariable logistic regression analysis. Correlations between T4 and L3 MRA and T4 and L3 SMA were evaluated using Pearson's correlations.

**Results:** T4 MRA and L3 MA were significantly correlated, whereas T4 SMA and L3 SMA were not (*r* = 0.43, *p* < 0.001, and *r* = −0.13, *p* = 0.09, respectively). Univariate analysis showed that T4 MRA was significantly associated with a higher risk of postoperative pneumonia (*N* = 21, 11,6%) (*p* = 0.01), whereas L3 MRA, L3‐index and T4‐index were not (*p* = 0.05, *p* = 0.10 and *p* = 0.62 respectively). In multivariate analysis, low T4 MRA remained significantly associated with an increased risk of postoperative pneumonia (p = 0.04 (OR 2.92, 95% CI 1.08–7.91).

**Conclusions:** Muscle radiation attenuation is a more reproducible body composition parameter across different body compartments than skeletal muscle area. Low muscle radiation attenuation assessed at the T4 level is predictive for postoperative pneumonia after partial hepatectomy.

**3–16**

**“Radiomics” features in body composition imaging associated with muscle wasting and poor survival after pancreatic cancer resection**

**Gregory van der Kroft** ^1^, Ralph Brecheisen^2^, Leonard Wee^3^, David P.J. van Dijk^2^, Sander S. Rensen^2^, Andre Dekker^3^, Roman Eickhoff^1^, Angalie A. Röth^1^, Florian Ulmer^1^ and Ulf P. Neumann^1,2^

^1^ *Department of General, Gastrointestinal, Hepatobiliary and Transplant Surgery, Uniklinikum RWTH‐Aachen, Aachen Germany, European HPB‐Center Aachen/Maastricht (ESCAM);* ^2^ *Department of Surgery, Maastricht University Medical Centre. European HPB‐Center Aachen/Maastricht (ESCAM). NUTRIM School of Nutrition and Translational Research in Metabolism, Maastricht University, Maastricht, the Netherlands;* ^3^ *Department of Radiation Oncology (MAASTRO), GROW, School for Oncology and Developmental Biology, Maastricht University Medical Center, Maastricht, The Netherlands*

**Introduction:** Muscle radiation attenuation (MRA) is associated with myosteatosis and mortality following pancreatic cancer surgery. Computerized radiological image analysis enables investigation of image‐derived phenotypes by extracting large numbers of quantitative features (radiomics). We identified radiomics features that correlated strongly with MRA and evaluated their association with mortality after surgery for head of pancreas cancer.

**Methods:** This was a retrospective multicenter study of 330 patients undergoing elective pancreatic cancer resection. Computed tomography images through the 3^rd^ lumbar vertebrae were manually segmented into muscle, visceral‐, and subcutaneous adipose tissue compartments. MRA was measured in the muscle compartment; in total, 339 radiomics features were extracted from all three compartments. Radiomics features associated with MRA were identified using Pearson correlation coefficients and tested for mortality association in univariate logistic regression. Multivariate logistic regression of MRA‐correlated radiomics features against mortality was performed using elastic regularization with 5‐fold cross‐validation and 20 sampling repetitions per fold.

**Results:** Eleven radiomics features were strongly correlated to MRA (*r* > 0.75, p < 0.001). In univariate analysis, all radiomics features were significantly associated to mortality at 90 days (*p* < 0.02) and 2 years (*p* < 0.03), compared to MRA alone (*p* < 0.005 and p < 0.03, respectively). Radiomics features slightly improved the Area‐Under‐receiver‐operator Curve (AUC) metric relative to MRA alone, both for 90‐day mortality (0.66 vs. 0.71, respectively) and 2‐year mortality (0.57 vs. 0.61, respectively) prediction.

**Conclusions:** Eleven radiomics features were found after univariate analysis to be strongly correlated to MRA, and significantly associated with mortality in patients undergoing pancreatic cancer resection. In multivariate logistic regression, radiomics features were associated with 90‐day and 2‐year mortality, respectively. We propose that radiomics features contain more information than MRA alone, leading to slightly improved discrimination. The robustness and veracity of radiomics features as predictors of post‐resection mortality need to be investigated further in a substantially larger multi‐institutional cohort.

**3–17**

**Preoperative skeletal muscle loss is highly associated to obstructive jaundice, but not to inflammatory biomarkers in resectable pancreatic cancer**

**Hosein Aziz**, Mustafa Suker, Jeroen van Vugh, Katya Mauff, Yordi van Putten, Jelle van Dongen, Joep Hermans, Jan Ijzerman, Nanda Krak and Casper van Eijck

*Erasmus Medical Center, Rotterdam, The Netherlands*

**Introduction:** Computed tomography (CT) ‐ determined skeletal muscle loss (SML) has shown to be a prognostic indicator in pancreatic cancer. Furthermore, approximately 70% of patients with pancreatic cancer have some degree of biliary obstruction at the time of diagnosis. Our aim was to identify factors associated with preoperative SML in patients with resected pancreatic ductal adenocarcinoma (PDAC) and to investigate the association between SML and postoperative outcome.

**Methods:** All pancreatic resections performed between 2004–2016 were assessed. SML was defined by CT imaging using previously established cut‐off values for the total cross‐sectional muscle area measured transversely at the third lumbar vertebra level (Figure 1). Baseline clinicopathologic characteristics, pre‐operative laboratory as well as follow up information, were collected. Obstructive jaundice was defined as serum bilirubin levels at presentation >35 μmol/L. We used logistic regression analysis to model SML as a function of gender, age, obstructive jaundice, CA19.9, systemic immune inflammation index (SIII), Glasgow Prognostic Score and histopathologic characteristics. Cox proportional hazards modelling were performed to identify independent prognostic factors for survival outcome.

**Results:** A total of 189 patients were eligible for analysis. Fifty‐eight percent of the patients had preoperative SML. Obstructive jaundice (OR 3.96, 95% CI: 1.50–10.5, *p* = 0.006), and high tumour stage (OR 4.64, 95% CI: 1.32–16.03, *p* = 0.017) were identified as independent predictors of preoperative SML. The SIII and GPS were not associated to preoperative SML. Furthermore, SML was independently associated with cancer‐specific survival (HR 1.98, 95% CI: 1.08–3.63, *p* = 0.028) as well as disease‐free survival (HR = 2.44, 95% CI 1.40–4.26, p = 0.002).

**Conclusions:** Patients with resectable PDAC with biliary obstruction at presentation have a three‐fold higher odds of pre‐operative SML compared to non‐jaundiced PDAC patients. Furthermore, SML is associated with cancer‐specific and disease‐free survival in resectable PDAC patients. Preoperative management of both obstructive jaundice and SML may prolong short and long‐term survival for these patients.

**3–18**

**Plasma metabolomics in oesophago‐gastric cancer patients: A pilot study**

**Janice Miller** ^*, 1^, Ahmed Alshehri^2^, Nathan A. Stephens^1^, Michael I. Ramage^1^, Stephen J. Wigmore^1^, James A. Ross^1^, David G. Watson^2" noteRef="jcsm12365-subcmp-0085-note-0004^ and Richard J.E. Skipworth^2^

^1^ *Department of Clinical Surgery, University of Edinburgh, Royal Infirmary of Edinburgh;* ^2^ *Strathclyde Institute of Pharmacy and Biomedical Sciences, University of Strathclyde, Glasgow G4 0RE, UK*

^*^Joint first authors

^**^Joint senior authors

**Introduction:** There are no agreed biochemical markers by which to diagnose cancer cachexia. The identification of early diagnostic markers would allow institution of prophylactic targeted therapy in patients undergoing surgical and oncological treatments.

We have previously performed transcriptomic analysis of rectus abdominis muscle biopsies from operative upper‐gastrointestinal cancer patients to identify new molecular biomarkers of cancer cachexia. Using plasma from the same patient cohort, we performed liquid chromatography‐ mass spectrometry (LC–MS) based metabolomics analysis to investigate the metabolic profile of cachexia.

**Methods:** Patients undergoing potentially curative surgery for oesophago‐gastric or pancreatic cancer were recruited. Fasting plasma samples were taken at induction of anaesthesia. Patients were divided into two groups on the basis of percentage weight loss from pre‐morbid stable weight (<5% and > 5%). LC–MS analysis was undertaken. Data extraction for each sample was carried out by MZmine software. Metabolites obtained were evaluated manually, and peak quality and retention times matched with standard metabolite mixtures. Univariate and multivariate analyses were performed using SIMCA‐P 14.1 software.

**Results:** Eighteen patients were recruited (*n* = 9 in each group). Metabolomic analysis showed clear separation of the groups. Overall a total of 37 metabolites were associated with weight loss. Metabolites with the highest fold change were species of LysoPC (lysophosphatidylcholine: ratio 1.78, *P* = 0.003) and LysoPE (lysophosphatidylethanolamine: ratio 1.79, *P* = 0.002). The largest affected group was the glycerophospholipids. Fatty acyls and products of lipid and amino acid metabolism were also increased.

**Conclusions:** This pilot study reveals a distinct metabolic profile of weight loss in operative oesophago‐gastric cancer patients. These results highlight the role of increased lipolysis in cancer cachexia, a mechanistic pathway that is currently under‐explored in human cachexia. Further biomarker development studies are required to validate LysoPC and LysoPE as early markers of cachexia in oesophago‐gastric cancer.

**3–19**

**Role of the multidimensional prognostic index in CKD older patients on conservative and replacement therapy**

**Alessio Molfino**, Silvia Lai, Maria Ida Amabile, Anna Paola Mitterhofer and Maurizio Muscaritoli and Study Group on Geriatric Nephrology of the Italian Society of Nephrology (SIN)

*Department of Clinical Medicine, Sapienza University of Rome, Rome, Italy*

**Introduction:** The prevalence of chronic kidney disease (CKD) is increasing, such as the number of CKD patients requiring dialysis. The prognostic evaluation of older adults with CKD plays a key role in decision‐making of treatment processes. The prognosis of older adults with CKD may be influenced by nutritional status, functional and cognitive status, as well as by the presence of comorbidities and therapies.

We aimed to evaluate the effectiveness of the multidimensional prognostic index (MPI) in predicting mortality risk and hospitalization, and the association with nutritional, metabolic and markers of atherosclerosis in older adults with CKD on conservative and replacement therapy.

**Methods:** CKD patients were enrolled and evaluated by MPI. Mortality rate, hospitalization rate, days of hospitalization and number of annual admissions during a 24‐month follow‐up were recorded. Clinical and nutritional parameters, including bioimpedance analysis, markers of atherosclerosis (intima media thickness), and ankle brachial pressure index, were recorded at baseline and at 24‐month. Parametric and non‐parametric analyses were performed, as appropriate.

**Results:** 173 CKD patients (107 males), with a mean age of 74.65 ± 8.43 years, on conservative and replacement therapy were consecutively enrolled in dialysis facilities in Italy. Three classes of risk were identified according to MPI score. We found a significant association between mortality rate and MPI for risk classes (ᵪ^2^=49.57, *P* < 0.0001). A positive correlation was seen between MPI and number of hospitalizations/year (*r* = 0.77, *P* < 0.001), and days of hospitalization (*r* = 0.73, P < 0.001). MPI correlated with age (*r* = 0.22, P = 0.003) and extracellular water at baseline (*r* = 0.23, *P* = 0.013).

**Conclusions:** MPI may be very useful to assess the prognosis of CKD patients. A multidimensional and multidisciplinary evaluation, including the analysis of fluid status by bioimpedance analysis, should be implemented in this population.

**3–20**

**Can a simple measure of task performance predict future tolerance of systemic anticancer treatment?**

**Anthony Byrne** ^2,3^, Jemima T. Collins^1,2^, Simon I. Noble^3^, John Chester^1,4^, Helen E. Davies^2^, Diane Parry^2^ and Jason F. Lester^4^

^1^ *Cardiff University;* ^2^ *Cardiff and Vale NHS Trust;* ^3^ *Marie Curie Research Centre Cardiff University;* ^4^ *Velindre NHS Trust*

**Introduction:** Assessment of muscle strength as well as mass is essential for diagnosis of sarcopenia. Although non‐receipt of planned cancer MDT treatments is common, objective measures of task performance are not routinely used to assess patients' readiness for treatment. Measurement of task performance may better reflect overall neuromuscular function as a proxy measure of frailty and poor tolerance of systemic anticancer treatment. A readily applicable tool in clinical settings would have important implications for MDT decision making and resource use. The Short Physical Performance Battery (SPPB) is a valid, responsive and reliable test of physical performance in older people. Its acceptability and feasibility in predicting completion of chemotherapy is unclear.

**Objectives:** We sought to establish whether SPPB might predict receipt and completion of MDT‐planned chemotherapy in Non Small Cell Lung Cancer (NSCLC).

**Methods:** Participants were recruited at first presentation to a Rapid Access Clinic. We assessed: performance on SPPB, body composition, PS and nutritional status. We recorded receipt and completion of chemotherapy, adverse effects, hospitalisations, and treatment delays.

**Results:** Of 62 participants, for 26 the MDT‐planned treatment was chemotherapy. Patients with a higher total SPPB score were likely to complete more cycles of chemotherapy as well as the full course. Quicker gait speeds and sit‐to‐stand times were associated with completion of three or more cycles of chemotherapy (all *p* < 0.05). For every unit increase in SPPB score, there was a 28.2% decrease in adverse events, hospitalisations and delays of chemotherapy (incidence rate ratio 0.718, *p* = 0.001).

**Conclusions:** SPPB is easy to undertake in clinical settings and may give better indication of likely chemotherapy treatment course completion than muscle mass alone and ECOG PS. This has implications for MDT decision‐making and prudent use of resources.

**3–21**

**Using NMR metabolomics to unravel the pathways underlying the host‐microbiota crosstalk in cancer cachexia**

**Sarah A. Pötgens**, Nathalie M. Delzenne and **Laure B. Bindels**

*Metabolism and Nutrition, Louvain Drug Research Institute, Université catholique de Louvain, Brussels, Belgium*

**Introduction:** The gut microbiota, considered as a crucial regulator of host immunity and metabolism, has recently been pinpointed as a promising therapeutic target for cancer cachexia. Indeed, previous studies demonstrated that, in a mouse model of leukaemia and cachexia, nutritional intervention targeting the gut microbiota has the potential to decrease cancer progression, inflammation, muscle atrophy and fat mass loss.

**Methods and results:** In this context, this research project aims to investigate new metabolic pathways potentially involved in the host‐microbiota interactions in cancer‐associated cachexia. Next‐generation sequencing and 1H‐NMR metabolomics are used to characterize both the microbial ecosystem and the host metabolism of cachectic mice (colon carcinoma 26 model). First results obtained by 1H‐NMR metabolomics of liver samples and next‐generation sequencing of cecal content demonstrate an important alteration of numerous liver metabolites as well as profound microbial changes in cachectic mice.

**Conclusions/Perspectives:** Following up on this work, we are currently performing 1H‐NMR metabolomics of cecal content, to further characterize the functions of the microbial ecosystem, as well as of blood samples, to get deeper insights in the host metabolism. In addition, through model and network buildings, we will pinpoint key microbes and related metabolites potentially involved in the microbiota‐host relationship in cancer cachexia. Finally, the relevance of such biological pathways will be validated in vivo.

**3–22**

**Bloom of *Enterobacteriaceae* in cancer cachexia: *Klebsiella oxytoca* as a gut pathobiont contributing to intestinal dysfunction**

Sarah A. Pötgens^1^, Hélène Brossel^1^, Martina Sboarina^1^, Emilie Catry^1^, Patrice D. Cani^1,2^, Audrey M. Neyrinck^1^, Nathalie M. Delzenne^1^ and **Laure B. Bindels** ^1^

^1^ *Metabolism and Nutrition Research Group, Louvain Drug Research Institute, Université catholique de Louvain, Brussels, Belgium;* ^2^ *Walloon Excellence in Life Sciences and BIOtechnology (WELBIO), Louvain Drug Research Institute, Université catholique de Louvain, Brussels, Belgium*

**Introduction:** Cancer cachexia is a complex multi‐organ syndrome characterized by body weight loss, weakness, muscle atrophy and fat depletion. With a prevalence of 1 million people in Europe and only limited therapeutic options, there is a high medical need for new approaches to treat cachexia. Our latest results highlighted microbial dysbiosis, characterized by a bloom in Enterobacteriaceae and altered gut barrier function in preclinical models of cancer cachexia. They also demonstrated the potential of targeting the gut microbial dysbiosis in this pathology. However, the exact mechanisms underlying the gut microbiota‐host crosstalk in cancer cachexia remain elusive.

**Methods and Results:** In this set of studies, we identified *Klebsiella oxytoca* as one of the main *Enterobacteriaceae* species increased in cancer cachexia and we demonstrated that this bacteria acts as a gut pathobiont by altering gut barrier function in cachectic mice. Moreover, we propose a conceptual framework for the lower colonization resistance to *K. oxytoca* in cancer cachexia that involves altered host gut epithelial metabolism and host‐derived nitrate boosting the growth of the gut pathobiont.

**Conclusions:** This set of studies constitutes a strong progression in the field of gut microbiota in cancer cachexia, by dissecting the mechanism of emergence of one bacterium, *K. oxytoca*, and establishing its role as a gut pathobiont in this severe disease.

**3–23**

**Loss of AMPK activity in muscle accelerates muscle mass loss and induces insulin resistance in cancer‐induced cachexia**

**Steffen H. Raun**, Xiuqing Han, Carlos Henriquez‐Olguín, Erik A. Richter and Lykke Sylow

*Section of Molecular Physiology, Faculty of Science, University of Copenhagen, Denmark*

**Introduction:** Cancer often leads to a marked loss of body mass, coined cachexia. Likewise, cancer‐induced cachexia has been associated with insulin resistance and whole‐body metabolic disturbances. Despite the clinical relevance, the cause of cachexia is still unknown. Increased activity of the metabolic sensor, AMP‐activated protein kinase (AMPK), has been observed in cachectic muscle, but the role of this kinase in cachexia is unresolved. This study aims to investigate the role of AMPK in cachectic muscle.

**Methods:** Mice overexpressing a dominant negative kinase mutant (kinase dead; AMPK KD) in skeletal and cardiac muscle were inoculated with Lewis Lung Carcinoma (LLC) cells at the flank. Non‐tumour bearing wild type littermates served as control. 16 days after tumour inoculation, glucose uptake was investigated using isotopic tracers during 10 min insulin stimulation (dose: 0.3 U/kg, injected retro‐orbitally in anaesthetized mice) (*n* = 7 for wild type groups, *n* = 6 for AMPK KD mice).

**Results:** Body weight, fat‐ and lean mass were similar between the three groups before tumour inoculation. Tumour‐induced cachexia was evidenced by a 30–35% reduction in abdominal adipose tissue that was similar between genotypes. For muscles, cachexia was only observed in tumour‐bearing AMPK KD mice, not wild type. Here, muscle weights were significantly reduced for tibialis anterior (−10.2%), EDL (−13.2%) and the heart (−12.8%) in AMPK KD mice compared to non‐tumour mice. Furthermore, whole body insulin response and muscle glucose uptake was decreased in AMPK KD mice compared to non‐tumour mice.

**Conclusions:** We conclude that AMPK activity may have an important and unrecognized protective role in cancer‐induced cachexia. Here, we demonstrate that loss of AMPK activity in muscle accelerates muscle mass loss and reduces whole‐body insulin sensitivity at a very early stage of cachexia in mice.

**3–24**

**The regulation of skeletal muscle fatigue by inflammation and activity during the progression of cancer cachexia**

**Brandon N. VanderVeen**, Dennis K. Fix, Brittany R. Counts and James A. Carson

*University of South Carolina, Columbia, USA*

**Introduction:** Cachexia progression has been linked to both systemic (e.g. chronic inflammation) and behavioural (e.g. inactivity) changes that compound to accelerate body weight and muscle mass loss. We have previously shown reduced volitional activity and increased skeletal muscle fatigue occur prior to cachexia development. The Apc ^*Min/+*^ (MIN) mouse, a preclinical model of cancer‐induced cachexia, is characterized by chronically elevated interleukin‐6 (IL‐6) and associated muscle signalling. Given the association between fatigue and cachexia's progression, understanding the regulation of skeletal muscle fatigue by IL‐6 and volitional activity provides intriguing therapeutic advantages.

**Purpose:** The purpose of this study was to determine the regulation of skeletal muscle fatigue during by activity and IL‐6 signalling during the progression of cachexia.

**Methods:** First, to determine the role of IL‐6 muscle signalling in cancer‐induced fatigue, we examined the functional properties of the tibialis anterior *in situ* in 18‐week male MIN and wildtype (WT) mice lacking the skeletal muscle IL‐6 receptor β (glycoprotein 130; KO). Next, we sought out to determine if elevated circulating IL‐6 alone induced skeletal muscle fatigue by overexpressing IL‐6 for 2 weeks in male WT mice.

**Results:** MIN mice had increased skeletal muscle fatigability (−22%) compared to WT following 90 seconds of a submaximal contraction‐induced fatigue protocol which was directly correlated to volitional activity (*R* = 0.82). Additionally, MIN‐KO mice had an attenuation (9%) in skeletal muscle fatigue compared to MIN. WT + IL‐6 had increased muscle fatigability (−20%) compared to vector controls concomitant with reduced respiratory control ratio and COX activity.

**Conclusions:** Together these results suggest that there is a relationship between volitional activity and increased skeletal muscle fatigue and high levels of IL‐6 may contribute to cancer‐induced skeletal muscle fatigability. Future studies should determine if increasing volitional activity can rescue skeletal muscle fatigue prior to cachexia development.

**3–25**

**Marked increased production of acute‐phase reactants (APR) by skeletal muscle during cancer cachexia**

**Isabelle Massart** ^1^, Geneviève Paulissen^2^, Laure Bindels^3^, Nathalie Delzenne^3^, Marie‐Alice Meuwis^2^, Edouard Louis^2^ and Jean‐Paul Thissen^1^

^1^ *Pole Endocrinology, Diabetes & Nutrition, University of Louvain, Bruxelles, Belgium;* ^2^ *Laboratory of translational Gastroenterology, University of Liège, Giga‐institute, Liège, Belgium;* ^3^ *Louvain Drug Research Institute, University of Louvain, Brussels*

**Introduction:** Reliable parameters for early diagnosis of cancer cachexia are still lacking and molecular mechanisms of muscle atrophy are incompletely unravelled. The goal of this study is to identify new pathways and potential biomarkers of muscle atrophy during cancer cachexia using the mouse models.

**Methods:** Ten days after C26 carcinoma cells SC injection, a classical model of cancer cachexia in mouse, gastrocnemius samples were collected for protein extraction and fractionation into sarcoplasmic (SF) and myofibrillar fractions (MF). Differential label free proteomics was used to compare muscle extracts from cachectic and control mice (n = 6/group) on a 2Dnano‐UPLC coupled to a QExactive Plus Hybrid Quadrupole‐Orbitrap mass spectrometer. Protein identifications and relative quantitations were obtained using MaxQuant and the differential analysis was performed using Perseus. Different softwares were used to identify the proteins potentially secreted.

**Results:** 963 proteins were obtained in SF and 935 in MF, with 228 differentially abundant between control and cachectic mice in SF and 198 in MF. Among the most significant upregulated and potentially secreted proteins during cachexia are several acute phase reactants (APR): Haptoglobin (Hp), Serum Amyloid A, Alpha‐1‐acid glycoprotein 1, C3 complement (C3) and Serpina3n. These changes were confirmed by Western Blot (*P* < 0.001) and RTq‐PCR (increase from 2‐ to 23‐fold; P < 0.001) indicating a local muscle production. Similar changes for Hp, C3 and Serpina3n were also observed in BaF3 cancer cachexia model. Myotube atrophy caused by glucocorticoids was associated with increased mRNA for most of these APR, confirming their increased production by muscle fibres and indicating the role of glucocorticoids in this induction.

**Conclusions:** Our study demonstrates the marked increased production of APR by skeletal muscle during cancer cachexia. Further studies are required to unravel the roles of these proteins in muscle atrophy and their interest as potential biomarkers of cancer cachexia.

**3–26**

**Maternal diet supplementation with leucine modulated the tumour‐induced muscle proteolysis in adulthood offspring**

**Natália Miyaguti Angelo da Silva**, Sarah Christine Pereira de Oliveira and Maria Cristina Cintra Gomes‐Marcondes

*Laboratory of Nutrition and Cancer, Department of Structural and Functional Biology; Institute of Biology (IB), State University of Campinas (UNICAMP), Sao Paulo, Brazil*

**Introduction:** The maternal nutrition, via epigenetics modifications, can modulate the cancer‐induced damage effects in offspring's adulthood. Leucine‐rich diet ameliorated the muscle proteolysis in cachectic tumour‐bearing rats. Thus, we investigated if maternal nutrition with leucine supplementation minimizes the muscle protein degradation in the adult offspring tumour‐bearing rats.

**Methods:** Adult offspring Wistar rats (n = 7–8) from mothers (intrauterine and weaning periods subjected to a control diet (normoprotein; C) or leucine‐rich diet (normoprotein plus 3% leucine; L), and fed a control diet until 120 days‐old, were distributed in groups according to the Walker‐256 tumour implant: C, control; W, tumour‐bearing; L, leucine without tumour; WL, leucine tumour‐bearing. After 21‐days of tumour evolution, gastrocnemius samples were analysed (CEUA #4224–1).

**Results:** All groups had the same body‐weight evolution during the lifetime. After the tumour inoculation, the WL group showed a lower final body‐weight (*P* = 0.0408), but both tumour‐bearing groups (W, WL) showed similar relative tumour weight. More interesting, the relative muscle weight was higher in the WL than the W group (*P* = 0.0158). Muscle tyrosine release increased only in W group; WL group had no difference compared to L. The muscle's 11S, and 19S proteasome subunits expression showed no difference, but the 20S subunit (28 kDa) increased only in the W group compared to C group (*P* = 0.0251), and WL and L groups had no difference. Chymotrypsin‐like activity remained unchanged among the groups, but calpain and cathepsin‐H activities increased only in W group. The serum 1‐methylhistidine increased only in W group (*p* = 0.0091), but the WL and L groups showed the same concentration.

**Conclusions:** The adult offspring tumour‐bearing rats, from mothers fed a leucine‐rich diet, had lower protein degradation, modulating the muscle mass loss. Therefore, the maternal nutritional supplementation likely showed a protective effect in muscle proteolysis. Other studies are undergoing to analyse the epigenetic markers that contributed to these changes.

**Support:** FAPESP; CNPq; CAPES; FAEPEX.

**4–01**

**Detection of sarcopenia and cachexia during the recording of the routine 12 channel ECG (Combyn™ ECG)**

**Falko Skrabal** ^1^, Katharina Skrabal^1^ and Jana Windhaber^2^

^1^ *Institute of Cardiovascular & Metabolic Medicine, Graz;* ^2^ *Department of Paediatric and Adolescent Surgery, Medical University Graz, Austria*

**Introduction:** Measurements of appendicular muscle mass (App MM) are not performed routinely, since accurate methods like whole body DXA (WBDXA) or segmental impedance spectroscopy are not widely available. Therefore we included methods for water, AppMM and fat measurements into the 12 channel ECG which is performed routinely.

**Methods:** A 12 channel ECG was modified as to enable segmental impedance spectroscopy in six body segments, namely the thorax, the abdomen and the four extremities (Med Eng Phys 44 (2017) 44–52). Only one electrode in addition to the 12 channel ECG is necessary. The impedances at different frequencies are used to calculate fat in all segments and AppMM at the extremities. In contrast to DXA our methodology can be corrected for extracellular fluid (ECF) excess, whereas the DXA measures also edema as muscle mass. The methodology was validated using WBDXA as reference method in 123 males and 153 females and was applied during the routine ECG in patients and athletes (976 male, 847 female) between the age of 9 and 97.

**Results:** There is a continuous rise of App MM in males and females peaking at 25 years, the slope of rise being steeper in males. Thereafter App MM declines in both sexes the slope of decline being much steeper in males. At very old age AppMM in both sexes is therefore nearly identical. Normal ranges were established for all age groups to avoid the use of the oversimplified standard where sarcopenia is based on 2 SD below the mean of young adults (Am J Epidemiol 147 (1998) 755–63). Examples of the application are shown in internal and neurologic diseases.

**Conclusions:** Since the methodology is performed unnoticed during the routine ECG and since it is not falsified by ECF accumulation as DXA, it could become a standard in medicine for application in all diseases associated with muscle loss.

**4–02**

**Muscle microRNAs predict muscle wasting following cardiac surgery**

Richard Paul, Mark Griffiths and **Paul Kemp**

*Section of Molecular Medicine, National Heart and Lung Institute, Imperial College London, UK*

**Introduction:** Muscle wasting is a common comorbidity of chronic conditions and an acute response to critical illness. We have developed a human model of muscle wasting, based on muscle loss following aortic surgery and shown that pre‐surgical muscle of a subset of miRNAs is associated with muscle loss. Here we quantified muscle expression of miRNAs before and after surgery to determine the extent to which they could predict muscle loss.

**Methods:** 46 patients undergoing aortic surgery requiring a post‐surgical ICU stay were recruited. Rectus femoris cross sectional area (RF_CSA_) was determined before and 7 days after surgery. An open muscle biopsy was taken prior to surgery and a closed muscle biopsy obtained 24 h later. miR‐424, 542‐3p/5p, 675, 519a, 518e, 485–3p and 422a were quantified by PCR.

**Results:** 50% of the patients lost >10% RF_CSA_ and were categorized as wasting. Pre‐surgical expression of miR‐424 and ‐542 were positively associated and that of miR‐422a and ‐518e negatively associated with muscle RF_CSA_ loss. Surgery increased the expression of miR‐424‐5p and − 542‐5p in all patients and miR‐675 in wasting patients only. Surgery reduced the expression of miR‐483‐5p in non‐wasting patients only. Logistic regression followed by ROC analysis for the pre‐surgical miRNA values gave an AUC of 0.87 (*p* < 0.0001) and detected muscle loss of >10% by day 7 in this group with a specificity of 83% and a sensitivity of 90%. Post‐surgical miRNA expression gave an AUC of 0.92 (p < 0.0001) and detected muscle loss of >10% in this study with a specificity of 95% and a sensitivity of 94%.

**Conclusions:** These data indicate that the pre‐surgical and 1‐day post‐surgical miRNA profiles have potential diagnostic capacity and indicate that miRNA expression is an important determinant of the susceptibility to muscle loss following surgery.

**4–03**

**Activin A causes human skeletal muscle cell atrophy by inhibiting slow myosin heavy chain synthesis**

**Audrey Loumaye**, Pascale Lause and Jean‐Paul Thissen

*Endocrinology, Diabetology and Nutrition Dept, IREC, Université Catholique de Louvain, Belgium*

**Background:** As we showed previously, cancer cachexia is associated with high circulating Activin A (ActA) levels, which are predictive of poor survival. In addition, several recent experimental evidences pinpoint the atrophying effect of ActA towards skeletal muscle, suggesting that ActA could predict poor survival in cancer patients by contributing to the loss of skeletal muscle mass.

**Aims:** Using a model of primary human skeletal muscle cells (SkMDC), we investigated the mechanisms involved in muscle atrophy induced by ActA.

**Results:** ActA (100 ng/ml during 48 h) caused atrophy of differentiated SkMDC, characterized by a reduction of the myotube diameter (−21%, *p* = 0.010), associated with a decrease of the cellular content in slow myosin heavy chain (MHC) (−45%, *p* = 0.013). The reduction of the slow MHC resulted from a downregulation of its gene expression (MYH7) (−45%, *p* = 0.0001). In contrast, we found no significant effect of ActA on MYH1 or MYH2 expression, encoding the fast MHC. Interestingly, expression of MYH7 was largely predominant in SkMDC in comparison to MYH1 (10 times less) and MYH2 (70 times less). ActA induced also a downregulation of the expression of MEF2C (−48%, *p* = 0.002), the main transcription factor regulating MYH7, which was also supported by a decrease of its transcription regulators, such as MyoD (−50%, p = 0,0003) and Myogenin (−51%, p < 0.0001). Finally, a lower transcriptional activity of MEF2C was indirectly suggested by the decrease of miR133 and a tendency to decrease of miR1, both downstream targets of MEF2C.

**Conclusions:** ActA causes atrophy of human skeletal muscle cells by repressing transcription and activity of MEF2C leading to a decrease in MYH7 transcription and slow MHC synthesis. Some factors known to increase MEF2C activity, such as exercise, should be assessed in order to prevent the atrophying effect of ActA on skeletal muscle and hence the development of cancer cachexia.

**4–04**

**Targeting muscle stem cells to restore regenerative capacity in aged muscle**

**Jerome N. Feige**

*Nestle Institute of Health Sciences, EPFL Campus, Lausanne, Switzerland;* *School of Life Sciences, Ecole Polytechnique Federale de Lausanne (EPFL), Lausanne, Switzerland*

**Introduction:** The remarkable ability of skeletal muscle to regenerate upon injury is conferred by tissue‐resident stem cells called satellite cells. With age, the regenerative capacity of muscle stem cells (MuSCs) dramatically declines. Developing strategies to enhance muscle repair in elderly people is therefore required, in particular to accelerate their recovery from injuries following falls or from surgical interventions affecting muscle tissues.

**Methods:** As the causes of MuSC dysfunction with age are multi‐systemic, we have investigated age‐related changes at different levels of the MuSC niche using stem cell isolation by flow cytometry, genomic and proteomic profiling, and therapeutic treatment with recombinant proteins in aged and genetically‐modified mouse models.

**Results & Conclusions:** Our results demonstrate that regenerative failure of skeletal muscle during aging involves altered extracellular matrix remodelling after injury, loss of cell communication in the stem cell niche, and perturbed secretion and signalling of apelin, a small peptide secreted in response to exercise. Targeting these mechanisms via fibronectin, the FAP‐derived matricellular protein WISP1, and the bioactive apelin peptide all provide novel therapeutic concepts to rescue skeletal muscle regenerative failure during aging by restoring muscle stem cell function.

**References**

Lukjanenko L, Jung MJ, Hegde N, Rudnicki MA, Fan CM, von Maltzahn J, Feige JN, and Bentzinger CF. Loss of fibronectin from the aged stem cell niche affects the regenerative capacity of skeletal muscle in mice. Nat Med 2016;22(8):897–905.

Vinel C, Lukjanenko L, Batut A, Pahor M, Feige JN, Vellas B, Valet P, Dray C. Nature Medicine. 2018 (In Press) https://doi.org/10.1038/s41591-018-0131-6

**4–05**

**Novel models of human pancreatic Cancer cachexia heterogeneity by tumor organoids Transplantation into mice**

**Merel Aberle** ^1^, Ludwig J. Dubois^2^, Rianne Vaes^1^, Natasja G. Lieuwes^2^, Rianne Biemans^2^, Ramon Langen^3^, Ronald van Dam^4^, Sander S. Rensen^1^ and Steven Olde Damink^1,4,5^

^1^ *Department of Surgery, NUTRIM School of Nutrition and Translational Research in Metabolism, Maastricht University, Maastricht, the Netherlands;* ^2^ *Department of Radiotherapy, The M‐lab group, GROW‐ School for Oncology and Developmental Biology, Maastricht University Medical Centre+, Maastricht, The Netherlands;* ^3^ *Department of Pulmonology, NUTRIM School of Nutrition and Translational Research in Metabolism, Maastricht University, Maastricht, the Netherlands;* ^4^ *Department of Surgery, Maastricht University Medical Centre+, Maastricht, The Netherlands;* ^5^ *Department of General, Visceral and Transplantation Surgery, RWTH University Hospital Aachen, Aachen, Germany*

**Introduction:** The poor survival of pancreatic cancer patients is largely attributable to cachexia, a syndrome of severe weight loss and muscle wasting. Tumour‐derived factors are thought to play an important role in the aetiology of cancer cachexia. To investigate systemic effects of such factors on host metabolism, we developed new pancreatic tumour organoid‐based mouse models.

**Methods:** Organoids were established from pancreatic ductal adenocarcinomas from two patients of whom cachexia severity was assessed pre‐operatively [body weight loss, body composition (e.g. skeletal muscle/adipose tissue mass using CT‐scans at L3 level), inflammation (e.g. CRP), and functional tests (e.g. handgrip strength)]. Organoids were dissociated into single cells; 20,000 cells or PBS were injected subcutaneously into the flanks of 9‐weeks old NMRI‐nude mice (*n* = 8/group). Body weight was monitored until sacrifice at 20 weeks to analyse tissues important in metabolism.

**Results:** Patients CL09 vs. CL12 showed 13.4% vs. 1.2% weight loss and a CRP of 20 vs. 1 mg/L. Transplantation of tumour organoids from CL09 and CL12 into mice resulted in tumour takes of 90% vs. 50%, and similar average tumour weight (34 vs. 33 mg). Alpha‐smooth muscle actin staining of tumour tissue revealed an extensive stromal reaction to both tumour organoids. Surprisingly, body weight gain of CL09‐transplanted mice did not differ from controls, whereas CL12‐transplanted mice displayed a significantly lower weight gain (0.7 gr. vs. 2.9 gram). However, wet weight of various muscles and liver weight did not differ between groups.

**Conclusions:** Cachexia parameters in mice transplanted with human tumour organoids are distinct from the clinical phenotype of the organoid donor. Nevertheless, the impact of CL12 tumour organoids on body weight suggests that tumour‐derived factors can directly or indirectly promote cachexia. Ongoing analyses of the inflammatory response and adipose tissue characteristics will shed light on additional relevant cachexia‐related parameters in these novel avatar models.

**4–06**

**Mouse limb and diaphragm muscles show distinct cachexia profiles in response to human pancreatic tumours**

**Rachel Nosacka** ^1^, Sarah Judge^1^, Michael Gerber^2^, Jose Trevino^2^ and Andrew Judge^1^

^1^ *Department of Physical Therapy, University of Florida, Gainesville, Florida, USA;* ^2^ *Department of Surgery, University of Florida, Gainesville, Florida, USA*

**Introduction:** Cancer cachexia is a life‐threatening metabolic syndrome that causes significant loss of skeletal muscle mass and significantly increases mortality in cancer patients. There is an urgent need for better understanding of the molecular pathophysiology of this disease so that effective therapies can be developed. Interestingly, almost all studies evaluating skeletal muscle response to cancer have focused on limb muscles, and almost all have focused on one or two pre‐clinical models. Therefore, the current study aimed to identify genome‐wide gene networks and biological processes in two distinct skeletal muscles in response to the same tumour using patient derived tumours.

**Methods:** Tumours resected from 5 pancreatic ductal adenocarcinoma (PDAC) patients were attached to the pancreas of immunodeficient NSG mice to generate Patient Derived Xenograft (PDX) mice, and tissues were harvested at IACUC mandated tumour endpoints.

**Results:** Body weight, muscle mass and fat mass were significantly decreased in each PDX line. H&E staining of cryosections taken from the tibialis anterior (TA) and diaphragm muscles revealed significant fibre atrophy in both muscles, but additional evidence of necrosis and mononuclear cell infiltration in the diaphragm but not the TA. Subsequent Masson's Trichrome staining also showed collagen deposition in the diaphragm but not the TA. Genome‐wide microarray analysis revealed similarities in biological pathways changed within the diaphragms of each PDX cohort, and separately within the TAs. In contrast, key differences were identified between TAs and diaphragms which align with our histological findings. Indeed, the diaphragm showed an enrichment of upregulated genes annotating to biological processes such as the extracellular matrix, inflammatory response and response to wounding, whereas genes annotating to the extracellular matrix were enriched among genes downregulated in the TA.

**Conclusions:** These data suggest that distinct biological processes are associated with different skeletal muscles in response to the same tumour burden.

**4–07**

**COPS2/TRIP15 protein content in skeletal muscle wasting in non‐small‐cell lung cancer patients: a novel target to be explored in cachexia?**

**Willian das Neves** ^1,2^, Christiano R.R. Alves^1^, Maria Janieire N.N. Alves^3^, Patricia C. Brum^1^ and Gilberto de Castro Jr^1,2^

^1^ *University of Sao Paulo;* ^2^ *Instituto do Cancer do Estado de São Paulo;* ^3^ *Instituto do Coração ‐ Hospital das Clinicas da Faculdade de Medicina da USP, São Paulo, Brazil*

**Introduction:** Most cancer patients in advanced stages are diagnosed with skeletal muscle wasting and cachexia, both associated with poor prognosis and with no effective treatment available. Recently, our group described a therapeutic role for endurance exercise training by reestablishing skeletal muscle function and improving survival in the Walker 256 cancer cachexia rat model. In an unbiased proteomics screen, COP9 signalosome complex subunit 2/Thyroid receptor‐interacting protein 15 (COPS2/TRIP15) was one of the most downregulated proteins in the skeletal muscle from tumour‐bearing untrained rats (−72%; *p* < 0.001), and endurance exercise training fully restored COPS2/TRIP15 protein expression. Given that COPS2/TRIP15 is highly conserved from Drosophila to humans, we hypothesized that COPS2/TRIP15 protein expression would change during cancer cachexia progression in humans.

**Methods:** To test this hypothesis, we are currently collecting muscle biopsies from metastatic non‐small cell lung cancer (mNSCLC) patients and control subjects.

**Results:** Our preliminary data (*n* = 5) showed that mNSCLC patients had lower aerobic capacity associated with decreased muscle COPS2/TRIP15 protein content compared to control subjects (−40%; *p* < 0.05). We are currently increasing the sample size of this cohort to evaluate potential correlations between COPS2/TRIP15 protein content and clinical data.

**Conclusions:** Animal model provided new insight into role of COPS2/TRIP15 during cancer cachexia progression, and preliminary data demonstrated that NSCLC patients had lower muscle COPS2/TRIP15 protein content than control subjects. These novel findings provide a preliminary signal for COPS2/TRIP15 as a potential target to develop new therapies for cachexia.

*Financial support FAPESP, Sao Paulo, Brazil (15/22814–5, 16/20187–6 and 16/01478–0).*

**4–08**

**11βHSD1 mediates a partial suppression of muscle atrophy in chronic inflammatory disease in response to therapeutic glucocorticoids**

**Justine Webster** ^1,2^, Chloe Fenton^1^, Gareth Lavery^1^, Ramon Langen^2^ and Rowan Hardy^1^

^1^ *University of Birmingham, Birmingham, United Kingdom;* ^2^ *Maastricht University, Maastricht, The Netherlands*

**Objective:** Therapeutic glucocorticoids (GCs) are commonly used in the treatment of chronic inflammatory disease. Unfortunately, their long‐term administration is associated with deleterious systemic side effects including muscle myopathy. 11 beta‐hydroxysteroid dehydrogenase type 1 (11β‐HSD1) activates GCs within muscle, is increased with inflammation, and has been shown to mediate GC‐induced muscle wasting. We examined how therapeutic GCs influence inflammatory myopathy and assessed the role of 11β‐HSD1 in this process.

**Methods:** Wild type (WT) and global 11β‐HSD1 knock out (KO) animals were crossed onto the TNF‐tg murine model of polyarthritis and inflammatory myopathy. Animals then received vehicle or the GC corticosterone (100 ug/ml) over three weeks in drinking water at doses sufficient to suppress signs of polyarthritis. Quadriceps and tibialis anterior were then harvested.

**Results:** GC activation by 11β‐HSD1 was completely attenuated in muscles from TNF‐tg^11β‐HSD1 KO^ animals. Both TNF‐tg and TNF‐tg^11β‐HSD1 KO^ animals developed inflammatory myopathy characterized by reduced muscle weights and fibre size. In the TNF‐tg mouse, therapeutic GC treatment further exacerbated muscle wasting with reduced muscle weight and fibre size relative to vehicle treated controls. This was characterized by elevated gene expression of atrophy markers (*foxo1, trim63*) and anti‐anabolic signalling (*redd1*), whilst pro‐inflammatory gene expression of *il‐6* was markedly suppressed. In contrast TNF‐tg^11β‐HSD1 KO^ animals receiving therapeutic GCs were protected from further muscle wasting, with muscle weights and fibre sizes significantly greater than TNF‐tg counterparts. These animals showed a partial protection from the elevated expression of the atrophy markers *foxo1, trim63* and anti‐anabolic marker *redd1*. They were also resistant to the suppression of the pro‐inflammatory gene of *il‐6*.

**Conclusions:** These data suggest that GCs and inflammation can act synergistically to induce muscle wasting during chronic inflammation and that 11β‐HSD1 appears to be an important factor in mediating the additive effects of corticosterone in vivo.

**4–09**

**Blockade of angiotensin II type 1 receptor in skeletal muscle stem (satellite) cells prevents angiotensin II‐induced skeletal muscle wasting**

**Tadashi Yoshida** and Patrice Delafontaine

*Department of Medicine and Medical Pharmacology and Physiology, University of Missouri School of Medicine, Columbia, MO, USA*

**Introduction:** Patients with advanced congestive heart failure (CHF) or chronic kidney disease (CKD) often have increased angiotensin II (Ang II) levels and cachexia. We previously demonstrated that Ang II infusion in rodents causes skeletal muscle wasting, likely contributing to cachexia in CHF and CKD. We also demonstrated that Ang II inhibits proliferation of skeletal muscle stem (satellite) cells (SCs) and reduces muscle regenerative capacity. We hypothesize that Ang II type 1 receptor (AT1R) signalling in SCs plays a role in developing Ang II‐induced muscle wasting.

**Methods:** We generated tamoxifen‐inducible, SC‐specific AT1R‐null mice (SC‐AT1R^−/−^) by crossing Pax7^CreER^ and AT1R‐floxed mice. By introducing Cre‐reporter gene in these mice, we labelled SCs and their progenies with EYFP. These mice were infused with Ang II (1.5 μg/kg/min), and muscle regeneration and wasting were analysed by qRT‐PCR, western blotting and immunohistochemistry.

**Results:** In hindlimb cardiotoxin injury model, Ang II reduced the number of regenerating myofibers (71.9% decrease, p < 0.001) and the expression of SC proliferation/differentiation markers MyoD and myogenin (56.5% and 62.5% decrease, respectively, p < 0.001). In contrast, SC‐AT1R^−/−^ mice were protected against these Ang II‐mediated reductions in muscle regeneration. In vitro, Ang II inhibited primary cultured SC proliferation, whereas AT1R‐null SCs were not affected. Importantly, SC‐AT1R^−/−^ mice restored skeletal muscle mass in high Ang II condition, likely due to the increased muscle regenerative capacity. SC lineage tracing and gene expression analyses revealed that, in the presence of high Ang II, AT1R‐deficient SCs generated higher number of newly formed/repaired myofibers (12.4 ± 2.7% of total myofibers in SC‐AT1R^−/−^ and ND in control, p < 0.001), resulting in the restoration of muscle mass and cross‐sectional area in SC‐AT1R^−/−^ mice.

**Conclusions:** These data indicate that inhibition of AT1R signalling in SCs could have a therapeutic potential to treat muscle wasting in chronic diseases with high Ang II, such as CHF and CKD.

**4–10**

**Angiotensin II inhibits autophagy and causes accumulation of dysfunctional mitochondria in mouse skeletal muscle**

Kleiton Augusto Santos Silva^1^, Patrice Delafontaine^1,2^ and **Tadashi Yoshida** ^1,2^

^1^ *Department of Medicine;* ^2^ *Department of Medical Pharmacology and Physiology, University of Missouri School of Medicine, Columbia, MO, USA*

**Introduction:** The ubiquitin‐proteasome system (UPS) and the autophagy‐lysosome system are two major intracellular protein degradation pathways. We have previously demonstrated that Angiotensin II (Ang II) activates the ubiquitin proteasome‐system and induces skeletal muscle wasting. However, it is unknown whether autophagy is involved in Ang II‐mediated muscle wasting, and we aimed to investigate the effects of Ang II on autophagy in mouse skeletal muscle.

**Methods:** Ang II was infused (1.0 μg/kg/min) in male FVB mice (12 weeks‐old) for 7 days; pair‐fed group was infused with saline and served as a control. Hindlimb muscles were harvested, and autophagy marker expressions were analysed by RT‐qPCR and western blotting. Mitochondrial morphology and function were analysed by transmission electron microscopy (TEM) and oxygen consumption rate (OCR) measurement, respectively.

**Results:** In the Ang II‐induced wasting condition (12% reduction in body weight, *p* = 0.0001), LC3B‐II conversion was blunted and p62/SQTSM1 expression was increased, indicating a reduction in autophagic flux. We found that Ang II inhibited phospho‐ULK1^Ser317^, leading to a reduction in phospho‐beclin1^Ser14^ and phospho‐ATG14^Ser29^, critical components of VPS34 complex that triggers autophagosome formation. On the other hand, lysosomal enzymatic activities (cathepsins) were not altered by Ang II. This reduction of autophagic flux likely resulted in an impaired clearance of damaged mitochondria in skeletal muscle, as TEM and OCR analyses revealed an accumulation of abnormal/dysfunctional mitochondria.

**Conclusions:** Our study indicates that Ang II impairs the initial step of autophagosome formation. The reduction of autophagy likely resulted in an insufficient clearance of damaged mitochondria, leading to an accumulation of dysfunctional mitochondria in skeletal muscle. Our data strongly suggest that autophagy defect plays a critical role in Ang II‐induced skeletal muscle wasting. Targeting autophagy pathway could lead to a development of novel therapeutic strategy to treat muscle wasting disorders with high circulating Ang II, such as congestive heart failure.

**4–11**

**BIO101 accelerates differentiation and enhances mitochondrial function in skeletal muscle cells**

**Maria Serova** ^1^, Blaise Didry‐Barca^1^, Sissi On^1^, Mathilde Latil^1^, Stanislas Veillet^1^, René Lafont^2^ and Pierre Dilda^1^

^1^ *Biophytis, Sorbonne Université, BC9, Paris, France;* ^2^ *Sorbonne Université, UPMC Univ Paris 06, Paris‐Seine Biology Institute (BIOSIPE), CNRS, Paris, France*

**Background:** Muscle wasting diseases such as sarcopenia, myopathies and cachexia are associated with the decline in differentiation of muscle cells into functional myofibers. This leads to a decrease in mobility and poor quality of life. The drug candidate BIO101 previously demonstrated its potential on muscle quality and function in different *in vitro* and *in vivo* models. BIO101 is the API of Sarconeos currently tested in clinical IIb trial in patients with sarcopenia.

**Objectives:** The aim of this study was to characterize the impact of BIO101 on muscle cells differentiation.

**Methods:** Mouse C2C12 myoblasts were induced to differentiate in 2% horse serum with and without BIO101, and MHC positive myotubes were studied under fluorescent microscopy up to 6 days of differentiation. The activation of AKT/mTOR signalling pathway was assessed by western blot. Oxygen consumption was measured using a Seahorse XF Analyser.

**Results:** BIO101 treatment induced a significant increase (>40%) of myofiber diameters consistently with a rapid activation of AKT/mTOR signalling pathway involved in muscle anabolism. Fusion index and number of nuclei per myotube were significantly increased after continuous exposure to 5 μM BIO101 consistently with an increased proportion of myogenin‐positive cells, suggesting an acceleration of differentiation process by the drug candidate. In accordance with improved myoblast differentiation, BIO101 increased mitochondrial mass (+75% at the end of differentiation in the presence of 5 μM drug) and oxygen consumption rate in myotubes (+23%, p < 0.05 in spare respiratory capacity).

**Conclusions:** This study demonstrates the overall beneficial properties of BIO101 on muscle cell differentiation. Increases in mitochondrial respiratory spare capacity and mitochondrial mass in response to BIO101 exposure are believed to be responsible for improved muscle function observed in preclinical models. These results support the clinical development of Sarconeos in sarcopenic patients.

**4–12**

**Combined administration of anti‐IL6 and anti‐PD‐L1 antibodies prevents ketogenic failure, reduces tumour progression, and increases overall survival in an autochthonous murine pancreatic cancer model**

**Miriam Ferrer** ^1^, Claire Connell^1^, Thomas Flint^1^, Daniele Biasci^2^ and Tobias Janowitz^1^

^1^ *Cancer Research UK Cambridge Institute, University of Cambridge, UK;* ^2^ *Cambridge Institute for Medical Research, University of Cambridge, UK*

**Introduction:** Tumour associated IL‐6 downregulates hepatic ketogenesis and thus predisposes to metabolic stress during caloric restriction associated with cachexia. Stress‐induced glucocorticoids are immune suppressive and can cause failure of cancer immunotherapy. Here, we investigate if this convergence of host metabolism and anti‐tumour immunity offers a target for combination therapy with anti‐IL‐6 and anti‐PD‐L1 antibodies.

**Materials and methods:** Combined anti‐IL‐6 and anti‐PD‐L1 antibodies (*n* = 13) or equimolar control antibodies (*n* = 12) were administered to mice bearing autochthonous pancreatic cancer (KPC: KrasLSL.G12D/+; p53R172H/+; Pdx‐Cre/+). Groups were matched and all mice received 1 dose of gemcitabine. Tumour volumes were monitored by ultrasound. Body weight and food intake were recorded and the biochemical profile of mice was characterized. For statistical analysis, differences between groups were determined using an unpaired two‐tailed Student's t‐test. Overall survival (OS) was analysed using the Kaplan–Meier method and by applying Log‐rank (Mantel‐Cox) statistics. Correlation between IL‐6‐JAK–STAT‐3 pathway and OS of patients from The Cancer Genome Atlas (TCGA) database was examined. Association of IL6 mRNA expression levels with OS was assessed in all cancer patients as well as specifically in patients with pancreatic cancer.

**Results:** OS increased significantly in anti‐IL‐6 and anti‐PD‐L1 treated mice compared to control mice (OS: 25 days vs. 11 days, *p* = 0.04). Day 7 measurements revealed delayed tumour progression in the combination immunotherapy cohort (volume increase in %: 118 +/− 5.4 vs. 138 +/− 5.6, *p* = 0.02). The combined analysis of expression from 55505 transcripts from 9345 patients using GSEA identified an association between IL‐6 pathway transcription and shorter OS (*p* < 0.0001). IL‐6 high expression significantly decreases OS in all cancer patients (*p* = 2.8E‐15), including patients with PDAC (*p* = 0.048).

**Conclusions:** Combination treatment with anti‐IL‐6 and anti‐PD‐L1 antibodies significantly increased OS and slowed tumour growth in an autochthonous mouse model of pancreatic cancer. The results of our work provide further justification to explore this treatment combination in cachectic patients with cancer associated elevation of IL‐6.

**4–13**

**Myeloid cell‐mediated inflammation protects against mouse liver cancer cachexia**

Merve Erdem^1,2^, Diana Möckel^3^, Twan Lammers^3^, Jochen Springer^4^ and **Thorsten Cramer** ^1,5^

^1^ *Molecular Tumour Biology, Department of General Surgery, RWTH University Hospital, Aachen, Germany;* ^2^ *Berlin School of Integrative Oncology, Charité Medical University, Berlin, Germany;* ^3^ *Institute for Experimental Molecular Imaging, RWTH University Hospital, Aachen, Germany;* ^4^ *Department of Cardiology and Pneumology, University of Göttingen Medical Center, Göttingen, Germany;* ^5^ *Department of General Surgery, University of Maastricht, Maastricht, The Netherlands*

**Introduction:** While different molecular mechanisms have been shown to be important for the pathogenesis of cachexia, systemic inflammation is considered to be one of the key driving factors. We investigated the molecular pathogenesis of hepatocellular carcinoma (HCC)‐associated cachexia to identify potential targets for therapy. To this end, we used a transgenic mouse model of HCC termed ASV‐B.

**Methods:** ASV‐B mice were crossed with mice harbouring a defect in myeloid cell‐mediated inflammation. Body weight and composition were analysed via NMR spectroscopy and microCT imaging. Lipolysis and fat browning were identified by biochemical and gene expression analyses. Macrophages in adipose tissue were investigated by immunohistochemistry. In a human HCC patient cohort (*n* = 93), CT images of L3 region were used to calculate body composition.

**Results:** We identified robust cachexia in the ASV‐B mouse model as evidenced by the loss of visceral fat and lean mass during tumour development. In addition, these mice showed elevated inflammatory markers in blood, increased fat mobilization ex vivo and browning of adipose tissue. Unexpectedly, the myeloid cell‐mediated inflammation defect resulted in enhanced fat loss without effecting inflammatory activity in the serum. Furthermore, defective myeloid cell‐mediated inflammation was associated with decreased macrophage infiltration and macrophage proliferation in visceral fat compared to WT mice. Analysis of our patient cohort revealed that 30% of HCC patients displayed reduced visceral fat mass reminiscent of the murine phenotype identified by us.

**Conclusions:** Our results show that a clinical relevant number of HCC patients display loss of visceral fat as part of the cancer cachexia phenotype. The analysed murine HCC model suggests a role for macrophages in the local regulation of cancer‐induced visceral fat wasting. Myeloid‐cell mediated inflammation displays a rather unexpected anti‐cachectic function our hands, questioning the general role of inflammation for the pathogenesis of cachexia.

**4–14**

**Group I Paks support muscle regeneration and counteract cancer‐associated muscle atrophy**

Andrea Cerquone Perpetuini^1^, Andrea David Re Cecconi^2^, Michela Chiappa^2^, Giulia Benedetta Martinelli^2^, Claudia Fuoco^1^, Giovanni Desiderio^1^, Luisa Castagnoli^1^, Cesare Gargioli^†, 1^, **Rosanna Piccirillo** ^†, 2^ and Gianni Cesareni^†, 1^

^1^ *Department of Biology, University of Rome Tor Vergata, Rome, Italy;* ^2^ *Department of Neurosciences, IRCCS‐Mario Negri Institute for Pharmacological Research, Milan, Italy*

^†^These authors contributed equally to this work and are corresponding authors

**Introduction:** Skeletal muscle is characterized by an efficient regeneration potential that is often impaired during myopathies. Previous studies revealed that the Ser/Thr kinase p21 protein‐activated kinase 1(Pak1) was specifically reduced in the atrophying gastrocnemius of Yoshida hepatoma‐bearing rats. So, we evaluated the role of group I Paks during cancer‐related atrophy and muscle regeneration.

**Methods:** We examined Pak1 expression levels in Tibialis Anterior muscles during cachexia induced by grafting colon adenocarcinoma C26 cells and in vitro by dexamethasone treatment. We investigated whether the overexpression of Pak1 counteracts muscle wasting in C26‐bearing mice and in vitro also during exposure to interleukin‐6 (IL6) or dexamethasone. Moreover, we analysed the involvement of group I Paks on myogenic differentiation using the group I chemical inhibitor IPA‐3.

**Results:** We found that Pak1 expression levels are reduced during cancer‐induced cachexia in the Tibialis Anterior of colon adenocarcinoma C26‐bearing mice and in vitro during dexamethasone‐related atrophy. Electroporation of muscles of C26‐bearing mice with plasmids expressing PAK1 preserves fibre size in cachectic muscles by restraining the expression of atrogin‐1 and MuRF1 and possibly by inducing myogenin expression. Consistently, the overexpression of PAK1 reduces the dexamethasone‐induced expression of MuRF1 in myotubes and increases the phospho‐ FOXO3/FOXO3 ratio. Interestingly, the ectopic expression of PAK1 counteracts atrophy in vitro by restraining the IL6‐Stat3 signalling pathway measured in luciferase‐based assays and by reducing rates of protein degradation in atrophying myotubes exposed to IL6. On the other hand, we observed that the inhibition of group I Paks has no effect on myotube atrophy and is associated with impaired muscle regeneration in vivo. In fact, we found that mice treated with the group I inhibitor IPA‐3 display a delayed recovery from cardiotoxin‐induced muscle injury.

**Conclusions:** Our data show group I Paks playing a central role in the regulation of muscle homeostasis, atrophy and myogenesis.

**4–15**

**The consequences of elevated p70S6K phosphorylation in rat soleus muscle at the early stage of mechanical unloading**

**Svetlana P. Belova**, Ekaterina P. Mochalova, Timur M. Mirzoev, Tatiana L. Nemirovskaya and Boris S. Shenkman

*Institute of Biomedical Problems RAS, Moscow, Russia*

**Introduction:** Disuse‐induced skeletal muscle wasting is associated with an imbalance between muscle protein synthesis and degradation. It was previously shown that p70S6K phosphorylation is paradoxically elevated in rodent soleus muscle at the early stage (12 h, 1‐ and 3 days) of mechanical unloading. However, the consequences of such p70S6K hyperphosphorylation on proteolytic and anabolic markers are poorly defined. The aim of the study was to evaluate the effect of elevated p70S6K phosphorylation on the key markers of proteolytic (FOXO3a, MuRF‐1 and MAFbx/Atrogin1) and anabolic (IRS‐1, AKT) signalling pathways as well as nuclear content of HDAC5.

**Methods:** The mechanical unloading was performed via hindlimb suspension (HS). 21 male Wistar rats were randomly divided into 3 groups: intact control (C), 1‐day HS (HS), and 1‐day HS plus rapamycin (mTORC1 inhibitor) treatment (1.5 mg/kg) (HS + R). Anabolic and catabolic markers were assessed using WB and RT‐PCR.

**Results:** One‐day HS resulted in a significant 65% increase in p70S6K phosphorylation level. Rapamycin treatment prevented the increase in p70S6K phosphorylation during unloading confirming the efficiency of the mTORC1 inhibitor. HS induced a significant increase in MuRF‐1 and MAFbx mRNA expression. In the HS + R group the expression of E3 ubiquitin‐ligases didn't differ from control group. The similar pattern was observed for MuRF‐1 protein expression. Increased p70S6K phosphorylation was accompanied by HDAC5 nuclear export, while rapamycin treatment returned the content of HDAC5 to the control levels. The phosphorylation of AKT and FOXO3a as well as total IRS‐1 content in the HS + R group was not altered as compared to HS alone.

**Conclusions:** The results suggest that the most probable mechanism of MuRF‐1 upregulation during an elevated p70S6K phosphorylation at the early stage of unloading is a termination of epigenetic blockade of E3 ubiquitin‐ligases expression. This study was supported by Russian Science.

Foundation grant No. 17–75‐20152.

**5–01**

**Transitioning from SARA‐OBS, an observational study to SARA‐INT, a double‐blind, placebo controlled, randomized clinical trial to evaluate safety and efficacy of Sarconeos (BIO101)**

**Waly Dioh** ^1^, Cendrine Tourette^1^, Carole Margalef^1^, René Lafont^1,2^, Philippe Dupont^1^, Pierre Dilda^1^, Stanislas Veillet^1^, Samuel Agus^1^ and Susanna Del Signore^3^

^1^ *Biophytis, Sorbonne Universités, UPMC – BC9, Paris, France;* ^2^ *Sorbonne Universités, UPMC Univ Paris 06, CNRS ‐ Institut de Biologie Paris Seine (BIOSIPE), Paris, France;* ^3^ *Bluecompanion ltd, London, United Kingdom*

**Introduction:** Sarcopenia, is characterized by the loss of muscle mass and muscle strength leading to a global muscle functional impairment and physical disability. SARA‐OBS, is the ongoing observational study dedicated to characterized the population for SARA‐INT, the interventional study with the investigational drug BIO101. Both studies are hosted in SARA‐Data, an innovative platform for clinical trials.

**Methods:** SARA‐OBS study consists of two main visits (baseline and 6‐month) to evaluate the main (400‐meter gait speed), and secondary endpoints (6‐minute walk, body composition, SPPB, grip strength and physical activity through actimetry). Patient Reported Outcomes and biomarkers of sarcopenia are also studied. Patients in SARA‐OBS study are recruited within centers in US and EU (France, Belgium and Italy), based on the FNIH criteria (Studenski et al..., 2014; ALM/BMI < 0.512 in women and < 0.789 in men or ALM <19.75 kg in men and < 15.02 kg in women) as well as SPPB ≤8. SARA‐INT shares the same inclusion criteria, will take place in 21 sites in EU and US and will consist of four main visits (baseline, Month1, Month3, and Month6). Newly recruited patients and patients retained from SARA‐OBS will be dosed at BIO101–175 mg b.i.d. and 350 mg b.i.d during 26 weeks versus placebo. The main end‐point is the gait speed at the 400‐meter walking test) as for SARA‐OBS.

**Results:** SARA‐OBS recruitment rates will be discussed including main screening failure reasons. Baseline characteristics including main (400 m gait speed) and key secondary criteria will be presented. Analysis of a first set of patients that have completed the investigational phase (ie 6 month visit) will be discussed. Baseline characteristics of the first randomized patients within the SARA‐INT study will be presented.

**Conclusions:** The SARA‐OBS study contributed to a better characterization of Age‐related Sarcopenia patients that will transition to SARA‐INT to evaluate safety and efficacy of BIO101.

**5–02**

**Role of cytomegalovirus serostatus on exercise‐induced decrease in senescence‐prone T‐lymphocytes in peripheral blood**

**Rose Njemini** ^1,2^, Hung Cao Dinh^1,2^, Ingo Beyer^1,2,3^, Oscar Okwudiri Onyema^1,2^, Keliane Liberman^1,2^, Liza De Dobbeleer^1,2^, Wim Renmans^4^, Sam Vander Meeren^4^, Kristin Jochmans^4^, Andreas Delaere^1,2^, Veerle Knoop^1,2^ and Ivan Bautmans^1,2,3^

^1^ *Frailty in Aging Research Group, Vrije Universiteit, Brussels, Belgium;* ^2^ *Gerontology Department, Vrije Universiteit, Brussels, Belgium;* ^3^ *Department of Geriatric Medicine, Universitair Ziekenhuis, Brussels, Belgium;* ^4^ *Laboratory of Haematology, Universitair Ziekenhuis, Brussels, Belgium*

**Introduction:** Aging is associated with a decline in immune function termed immunosenescence (IS). This process is characterized among others by less naïve T‐cells, and more terminally differentiated senescence‐prone phenotypes, which have been implicated in the pathogenesis of many age‐related conditions including sarcopenia. Although cross‐sectional studies indicate that regular exercise may combat the adverse effects of IS, reports regarding the long‐term adaptation effects of exercise on T‐cell phenotypes have been largely equivocal. These inconsistencies may be due to potential contributors to IS, particularly cytomegalovirus (CMV) infection, which is considered a hallmark of T‐cell senescence. Therefore, we sought to investigate the impact of CMV serostatus on T‐cell response to long‐term exercise in older women.

**Participants and Methods:** One hundred older women (aged ≥65 years) were randomized to 2–3 times/weekly training for 6 weeks at either intensive strength training (3x10 repetitions at 80% one‐repetition maximum (1RM), *n* = 31), strength endurance training (SET, 2x30 repetitions at 40% 1RM, *n* = 33), or control (flexibility training, FT, *n* = 36). Anti‐CMV antibodies were measured using Architect iSystem. The T‐cell percentage and absolute blood counts were determined before and after 6 weeks (24 h–48 h after the last training session) using flow cytometry and haematology analyser.

**Results:** As expected, CMV‐seropositivity was significantly associated with less naive cells, more memory and senescence‐prone phenotypes. We found that 6 weeks of SET significantly decreased senescence‐prone T‐cells along with a concomitant increase in naïve T‐cells in peripheral blood. Intriguingly, these changes were observed in CMV‐seropositive, but not CMV‐seronegative individuals.

**Conclusions:** In conclusion, the results suggest that SET may limit the accumulation of senescence‐prone T‐cells and decline in naive T‐cells in CMV‐seropositive older subjects, which is attractive given our aging population, and the potential implications that T‐cell IS has for increasing susceptibility to morbidity. SET might have more relevance in restoring a compromised immune system.

**5–03**

**New biomarkers and the potential of bioelectrical impedance vector analysis for the assessment of muscle wasting**

**Nicole Ebner** ^1,2^, Lorena Lerner^3^, Jeno Gyuris^3^, Breno Godoy^1,2^, Ruben Evertz^1,2^, Goran Loncar^4,5^, Miroslava Valentova^1^, Mirela Vatic^1^, Jochen Springer^1^, Stefan D. Anker^6,7^, Wolfram Doehner^8^ and Stephan von Haehling^1,2^

^1^ *Department of Cardiology and Pneumology, University Medical Center Göttingen, Göttingen, Germany;* ^2^ *DZHK (German Centre for Cardiovascular Research), partner site Goettingen, Goettingen, Germany;* ^3^ *AVEO Pharmaceuticals, Inc., Cambridge, Massachusetts;* ^4^ *Institute for cardiovascular disease Dedinje, Belgrade, Serbia;* ^5^ *Faculty of medicine, University of Belgrade, Serbia;* ^6^ *Division of Cardiology and Metabolism ‐ Heart Failure, Cachexia & Sarcopenia, Department of Cardiology (CVK), Charité University Medical Center Berlin, Germany;* ^7^ *Berlin‐Brandenburg Center for Regenerative Therapies (BCRT), Charité University Medical Center Berlin, Germany;* ^8^ *Centre for Stroke Research Berlin, Charité ‐ University Medical School, Berlin, Germany*

**Purpose:** The aim of this study is to investigate whether bioelectrical impedance vector analysis (BIVA) can be a suitable technique for the assessment of muscle wasting. We also analysed different potential biomarkers and exercise capacity.

**Subjects and Methods:** We included in our study 164 patients with stable chronic heart failure (HF) of both sexes, with a mean age of 65 years. Dual energy X‐ray absorptiometry (DEXA) was used as the reference method for identifying sarcopenic or muscle wasting individuals. The BIVA procedures, which respectively correct bioelectrical values for body height and body geometry, were used. Additionally we analysed growth differentiating factor 15 [GDF‐15], Activin A, TNF α and Interleukin‐1. Exercise capacity was measured with spiroergometry [peak VO2] and six minute walk test [6MWT]. The quality of life was analysed by using the Kansas City Cardiomyopathy Questionnaire [KCCQ]. Muscle wasting was defined as the appendicular muscle mass 2 standard deviation below the mean of a healthy reference group of adults aged 18–40 years, as suggested for the diagnosis of muscle wasting in healthy aging. Cachexia was defined as non edematous weight loss of 5% in 6 month or more.

**Results:** We found that 26 patients showed cachexia without muscle wasting and 27 patients showed muscle wasting and without cachexia. A total of 10 patients were found with cachexia and muscle wasting. Patients with cachexia showed significantly higher GDF‐15 (mean patients with cachexia 2.9 ± 1.4 vs. without: 1.9 ± 0.85 *p* = 0.01) and Interleukin 1b levels (mean patients with cachexia: 11.7 ± 6.19 vs. mean without: 10.7.42 ± 6.18 *p* = 0.02). Exercise capacity was significantly reduced in patients with cachexia (mean peak VO2:15.8 ± 5.16 kg/mL*min) compare to without cachexia (mean peak VO2:17.03 ± 4.42; *p* < 0.05). Patients with cachexia showed reduced quality of life (KCCQ symptom score: 54.2 ± 27.1, KCCQ symptom stability:54.2 ± 27.0, KCCQ quality of life score:61.6 ± 26.2, KCCQ overall summary score:54.4 ± 12.6). Quality of life scores are correlated with reduced exercise capacity. Patients with muscle wasting showed significantly higher values of resistance/height (R/H; *p* < 0.01) and reactance/height (X/H; p < 0.01), and a lower phase angle (p < 0.01). Moreover we show a significant correlation between appendicular mass and resistance/height (r = +0.151; *p* < 0.0001) and reactance/height (r = +0.047; *p* = 0.04). GDF‐15, Activin A and IL‐1 were not correlated with muscle wasting.

**Conclusions:** BIVA detected muscle‐mass variations in patients with muscle wasting. These procedures are promising tools for screening for muscle wasting in routine practice. Patients with HF showed reduced exercise capacity and reduced quality of life and both are correlated with each other. Moreover we have shown that patients with cachexia showed higher GDF‐15 and Interleukin‐1 levels.

**5–04**

**Αcute effects of essential amino acid gel‐based and whey protein supplements on appetite and energy intake in older women**

**Theocharis Ispoglou**, Matthew Lees, Paul Harlow, Lauren Duckworth, Karen Hind and Matthew Butteworth

*Leeds Beckett University, Carnegie School of Sport, Leeds, United Kingdom*

**Introduction:** Anorexia and the satiating effects of dietary protein are partly responsible for deficiencies in protein (PI) and energy intakes (EI), which are contributing factors to age‐related sarcopenia. We have demonstrated that essential amino acids (EAA) supplements facilitate an increase in PI and EI of older women. However, it is not known whether supplementation with different forms of protein supplements providing equivalent amounts of EAA can be as equally effective at increasing EI. We therefore investigated the effect of supplements matched in EAA content (7.5 g) on EI and appetite.

**Methods:** Ten women aged 69.2 ± 2.7 years, completed three trials in a randomized, crossover design. Composite appetite scores, peptide YY, and insulin responses to a 200 ml whey protein (WP) isolate (275 kJ), a 50 ml EAA gel (478 kJ) or a control (CON) were investigated over one hour, followed by an ad libitum breakfast.

**Results:** EI at breakfast (CON 1957 ± 713, WP 1413 ± 623, EAA 1963 ± 611 kJ) was higher in the CON and EAA than the WP (both *P* = 0.006). After accounting for supplement energy content, EI in the EAA was higher than the CON (*P* = 0.0006) and WP (*P* = 0.0008). Time‐averaged area under the curve for composite appetite scores (CON 74 ± 20, WP 50 ± 22, EAA 60 ± 16 mm) was higher in CON than WP (*P* = 0.015). Time‐averaged area under the curve for peptide tyrosine tyrosine (CON 87 ± 13, WP 119 ± 27, EAA 97 ± 22 pg·mL‐1) was higher in WP than CON (*P* = 0.009) and EAA (*P* = 0.012).

**Conclusions:** Supplementation with WP facilitated an increase in PI, whereas supplementation with an EAA gel facilitated an increase in both PI and EI, therefore may be potential means for addressing nutritional age‐related sarcopenia.

**5–05**

**DPA shows comparable cellular chemotherapy sensitizing effect as EPA upon cellular incorporation**

**Francina J. Dijk** ^1^, Bram Dorresteijn^1^, Klaske van Norren^2^, Marion Jourdan^1^, Ardy van Helvoort^1^ and Miriam van Dijk^1^

^1^ *Danone Nutricia Research, Nutricia Advanced Medical Nutrition, Utrecht, The Netherlands;* ^2^ *Nutrition and Pharmacology, Wageningen University, Wageningen, The Netherlands*

**Introduction:** Dietary supplementation with ω‐3‐polyunsaturated fatty acids (PUFAs) has shown beneficial effects on cancer and treatment outcomes. Compared to eicosapentaenoic acid (EPA) and docosahexaenoic acid (DHA), the role of ω‐3‐PUFA docosapentaenoic acid (DPA) has been studied less. We hypothesize that DPA could have similar effects as EPA and DHA in cancer and during its treatment.

**Methods:** C26‐adenocarcinoma cells were pre‐treated (4 days) with EPA, DHA or DPA (50 μM) and treated with doxorubicin or cisplatin. Cellular PUFA incorporation and chemo‐sensitivity was measured by cell gas‐chromatography, cell viability (WST) and apoptosis (caspase‐3/7). Subsequently, incorporation of PUFAs in tumour and muscle tissue was studied in a C26‐tumour‐bearing (TB) mouse model after supplementation with EPA‐containing diet supplements (fish‐oil, pure‐EPA or tuna‐oil). In healthy volunteers, PUFA incorporation was measured in plasma, RBC and WBC at five timepoints following a nutritional supplement intervention containing fish‐oil for 7 days (Faber et al 2011).

**Results:** Both EPA and DPA showed similar chemotherapy‐enhancing properties by increasing chemotherapy sensitivity in C26‐adenocarcinoma cells. DPA incorporation was significantly increased in cells treated with EPA and DPA (*P* < 0.0001) when compared to control treatment. In TB‐mice, supplementation with fish‐oil and pure‐EPA, both high in EPA content and low in DPA, resulted in a twice as high incorporation of DPA in tumour and muscle tissue when compared to EPA. In line with the preclinical results, healthy volunteers showed a significant increase in EPA and DPA in WBC and plasma (*P* < 0.001) following supplementation with a fish‐oil containing nutritional intervention.

**Conclusions:** Supplementation with EPA or EPA‐containing supplements leads to increased cellular EPA and DPA. High DPA incorporation is mainly driven by the elongation of incorporated EPA. Both EPA and DPA supplementation showed chemotherapy‐enhancing effects *in vitro;* however, the role of DPA requires more research.

**5–06**

**Exploring the feasibility of a combined package of care for patients undergoing neo‐adjuvant chemotherapy for pancreatic cancer: The FEED Study (a Fish oil supplement, pancreatic Enzyme supplement, Exercise advice and individualized Dietary counselling)**

**Oonagh Griffin** ^1,2^, Sinead Duggan^1^, David Fennelly^3^, Raymond Mc Dermott^3^, Justin Geoghegan^2^ and Kevin Conlon^1,2^

^1^ *Professorial Surgical Unit, Centre for Pancreatico‐ biliary Disease, Trinity Centre for Health Sciences, Tallaght Dublin, Ireland;* ^2^ *National Surgical Centre for Pancreatic Cancer, St Vincent's University Hospital, Elm Park, Dublin, Ireland;* ^3^ *Department of Medical Oncology, St Vincent's University Hospital, Elm Park, Dublin, Ireland*

**Introduction:** Cancer cachexia is highly prevalent in pancreatic cancer, and muscle depletion is an established adverse prognostic factor when combined with obesity during palliative chemotherapy. The development of more potent chemotherapy agents has led to a new era in the management of locally advanced and borderline resectable pancreatic cancer. Recent work has confirmed that accelerated muscle loss occurs during neoadjuvant chemotherapy, significantly increasing mortality.

**Methods:** We designed an interventional study of a multi‐modal package of care designed to prevent loss of muscle in patients with pancreatic cancer, specifically intensive nutritional counselling, pancreatic enzyme replacement therapy (PERT), an eicosapentaenoic acid‐enriched oral nutritional supplement (ONS), and an individualized daily step target. The primary objective is to determine the feasibility of this 12‐week intervention in 20 patients receiving neoadjuvant chemotherapy before pancreatic resection. Specifically, tolerance of supplementation, PERT, and physical activity are measured, along with barriers to acceptance. Secondary objectives are to investigate change in body composition (CT‐based assessment), inflammatory cytokines (IGF‐1, IL‐6, TNF‐α), functional capacity (grip strength, Timed‐Up‐and‐Go), and quality of life (EORTC).

**Results:** To date, 12 of 18 eligible patients undergoing neoadjuvant treatment (Folfirinox or Gemcitabine with Nab‐Paclitaxel) at our institution over a ten‐month period have been recruited, representing 60% accrual. Nine patients have completed the intervention to date. Preliminary analysis of the feasibility of the intervention shows that 100% of patients attended fortnightly dietetic appointments, with 89%, 78% and 67% meeting PERT, ONS and step target prescriptions respectively. Barriers to meeting ONS prescription included taste aversion/fatigue (increased compliance when PERT was established), while barriers to meeting physical activity included chemotherapy‐induced neuropathy, treatment‐related side effects, and fear of acquiring infection. Secondary objectives will be analysed upon study completion, with an expected accrual end‐date of December 2018.

**Conclusions:** Preliminary data highlight high patient opt‐in and acceptance of this multi‐modal intervention.

**5–07**

**Skeletal muscle structure and energy metabolism in cancer cachexia: clinical approach**

**Adeline Dolly**, Julie Cournet, Jean‐François Dumas and Stéphane Servais

*INSERM UMR1069, “Nutrition, Croissance et Cancer”, University of Tours, France*

**Background:** Evidence from pre‐clinical models of cancer cachexia and key works on muscle cells suggest mitochondrial dysfunctions, lipid droplets accumulation and sarcoplasmic reticulum (SR) stress to be important factors that could contribute to the development of skeletal muscle atrophy associated with cancer cachexia. However, no clinical studies on muscular mitochondrial bioenergetics or (SR) stress have been performed yet. Our objective is to investigate the metabolic and structural dysfunctions in skeletal muscle from cachectic patients.

**Methods:** An ongoing Clinical Pilot Study METERMUCADIG (NCT02573974) is including 45 patients with pancreatic or colorectal cancer. Pectoral muscle biopsy and blood samples are collected before chemotherapy. Different analyses are performed, including evaluation of body composition and myosteatosis on CT‐Scan; analyse of cytoplasm, mitochondria, and lipid droplets on skeletal muscle electron micrographs; measure of muscle fibres surface and mitochondrial oxygen consumption; and quantification of mRNA expression of SR stress markers on muscle biopsies.

**Results:** 32 of the 45 patients have been included so far. Our preliminary data on muscle biopsies seem to show, for the first time in a clinical study on cancer cachexia, a structural modification of cytoplasm aspect in the pectoral skeletal muscle of patients. We also observe intramyocellular lipid droplets and a heterogeneity in body composition, muscle fat infiltration and muscle fibres surface among patients. Some patients, despite same sex, age, cancer, BMI and SMI, show huge body composition and skeletal muscle structural differences, which question actual criteria used to detect cachexia.

**Conclusions:** At the end of patients' inclusion, SMI thresholds will be calculated. Then all data collected will be processed to have for the first time evidences for links between patients' skeletal muscle bioenergetics alterations, structural defects and body composition.

**5–08**

**Skeletal muscle collagen content is increased in cachectic pancreatic cancer patients and is a significant predictor of survival**

**Sarah M. Judge** ^1^, Daniel Delitto^2^, Rachel L. Nosacka^1^, Miles E. Cameron^2^, Michael H. Gerber^2^, Jose G. Trevino^2^ and Andrew R. Judge^1^

^1^ *Department of Physical Therapy, University of Florida Health Science Center, Gainesville, FL, USA;* ^2^ *Department of Surgery, College of Medicine, University of Florida Health Science Center, Gainesville, FL, USA*

Cancer cachexia is a devastating catabolic condition characterized by skeletal muscle wasting, with or without fat wasting, consequent to tumour burden which negatively impacts tolerance to cancer therapies and contributes to 30% of cancer‐related deaths. However due, in part, to limited knowledge of the underlying mechanisms of cancer cachexia derived from human studies, the ability to therapeutically intervene remains elusive. The purpose of the current study was therefore to better define the phenotype of skeletal muscle obtained from patients with pancreatic ductal adenocarcinoma (PDAC), which has one of the highest rates of cachexia. Morphological and transcriptional analyses were performed on rectus abdominis muscle biopsies obtained from *N* = 20 resectable PDAC patients undergoing tumour resection surgery and from *N* = 16 weight‐stable non‐cancer controls undergoing benign abdominal surgery. PDAC patients with a body weight (BW) loss of >5% over the previous 6 months were considered cachectic (*N* = 15 cachectic, *N* = 5 non‐cachectic). Compared to controls, the percent of muscle occupied by collagen was significantly increased in skeletal muscle from cachectic, but not non‐cachectic PDAC patients. Across all PDAC patients, collagen content positively correlated with body weight loss, was significantly higher in patients with lymph node metastasis and was a significant predictor of survival on univariate and multivariate analyses. The fibrotic phenotype in cachectic PDAC patients was also associated with increased abundance of fibroadipogenic progenitors, lipid deposition, macrophage infiltration, calcium deposition and evidence of deficient cellular quality control mechanisms compared to controls. Transcriptional profiling of all patients supported these findings by identifying gene clusters related to wounding, inflammation, and cellular response to transforming growth factor beta (TGF‐β) upregulated in cachectic PDAC patients compared to controls. In summary, this work is the first to demonstrate a fibrotic phenotype in cachectic PDAC patients that positively correlates with body weight loss and which associates with poor survival.

**5–09**

**Chemoradiation induced changes in body composition and the response to tube‐feeding in locally advanced squamous cell carcinoma of the head and neck**

**A.C.H. Willemsen** ^1,2,3^, **Ann Hoeben** ^1,2^, Roy I. Lalisang^1,2^, Ardy Van Helvoort^3,4^, Laura W.J. Baijens^2,5^, Frederik W.R. Wesseling^2,6^, Frank Hoebers^2,6^ and Annemie M.W.J. Schols^3^

^1^ *Division of Medical Oncology, Department of Internal Medicine, Maastricht University Medical Centre+, The Netherlands;* ^2^ *GROW‐School of Oncology and Developmental Biology, Maastricht University Medical Centre+, The Netherlands;* ^3^ *Department of Respiratory Medicine, NUTRIM School of Nutrition and Translational Research in Metabolism, Maastricht University Medical Center+, Maastricht, The Netherlands;* ^4^ *Danone Nutricia Research, Nutricia Advanced Medical Nutrition, Utrecht, The Netherlands;* ^5^ *Department of Otorhinolaryngology, Head & Neck Surgery, Maastricht University Medical Centre+, The Netherlands;* ^6^ *MAASTRO Clinic, Department of Radiation Oncology, Maastricht, The Netherlands*

**Introduction:** Cachexia is a common problem in patients with locally advanced squamous cell carcinoma of the head and neck (LAHNSCC). Treatment with chemo‐ or bioradiation (CRT) may induce further weight loss and the need of tube‐feeding (TF). Yet, changes in body composition during treatment are underexplored, and strong evidence on the effect of TF on the course and composition of weight loss during therapy is lacking. Aim of this prospective cohort study (ClinicalTrials.gov Identifier: NCT01985984) was to evaluate changes in body composition during CRT and to study the effect of the current TF regime on body composition.

**Methods:** 69 LAHNSCC patients treated with CRT between January 2013 and December 2016 were included. Weight loss, body composition (bioelectrical impedance analysis), and hand grip strength (HGS) were assessed at baseline, in week 3–4 and 1–2 weeks after CRT. TF was started according to the Dutch guidelines for malnutrition [Kruizenga et al. Richtlijn Ondervoeding].

**Results:** In the total cohort, incidence of cachexia was 29% at diagnosis, increasing to 36% after CRT completion. Weight and HGS significantly decreased during CRT; −3.7 ± 3.5 kg (*p* < .001) and − 3.1 ± 6.0 kg (p < .001) respectively. 48/69 patients required TF, started with a median of 21 days after CRT initiation. 21/69 patients remained on total oral diet. Between‐group analysis showed a significantly (*p* = 0.007) higher total weight loss during CRT in the subgroup remaining on oral diet (5.5 ± 3.7 kg) compared to TF subgroup (3.0 ± 3.2 kg). Loss of Fat Free Mass (FFM) and HGS however was comparable between the groups.

**Conclusions:** Patients undergoing CRT for LAHNSCC have significant loss of weight and FFM during treatment. Current TF nutritional regime applied in this study attenuates weight loss but does not overcome loss of muscle mass and function during therapy. Future interventions should consider more proactive feeding and strategies specifically targetting loss of muscle mass and function.

**5–10**

**Muscle wasting is an independent prognostic factor in patients with locally advanced head and neck cancer treated with chemo‐ or bioradiation and predicts treatment toxicity but not tumour control**

**Ann Hoeben** ^1,2^, A.C.H. Willemsen^1,2,5^, Roy I. Lalisang^1,2^, Laura W.J. Baijens^2,3^, Frederik W.R. Wesseling^2,4^, Frank Hoebers^2,4^ and Annemie M.W.J. Schols^5^

^1^ *Division of Medical Oncology, Department of Internal Medicine, Maastricht University Medical Centre+, The Netherlands;* ^2^ *GROW‐School of Oncology and Developmental Biology, Maastricht University Medical Centre+, The Netherlands;* ^3^ *Department Otorhinolaryngology, Head & Neck Surgery, Maastricht University Medical Centre+, The Netherlands;* ^4^ *MAASTRO Clinic, Department of Radiation Oncology, Maastricht, The Netherlands;* ^5^ *Department of Respiratory Medicine, NUTRIM School of Nutrition and Translational Research in Metabolism, Maastricht University Medical Center+, Maastricht, The Netherlands*

**Introduction:** Patients (≤70 years) with locally advanced head and neck cancer (LAHNSCC) are being treated with chemo‐ or bioradiation (CRT). Comorbidities define the radiosensitizer: cisplatin (chemoradiation) versus cetuximab (bioradiation). Body composition is currently not a critical factor in this treatment choice. Curation rates of CRT are mostly defined by tumour stage and ‐biology. The impact of body composition in LAHNSCC patients on the prognosis, CRT toxicity and –outcome is not established. Aim of this prospective cohort study (ClinicalTrials.gov Identifier: NCT01985984) is to evaluate baseline body composition in LAHNSCC treated with chemoradiation versus bioradiation and to assess the influence of muscle wasting on median overall survival (OS), CRT toxicity and –outcome.

**Methods:** 137 LAHNSCC patients treated with CRT between January 2013 and December 2016 were included. Body composition (bioelectrical impedance analysis) was assessed at baseline. Grade III and IV toxicity (CTCAE 4.03) was evaluated. Clinical locoregional tumour response was defined 2–3 months after CRT completion and OS analysis was performed within a median follow‐up of 32 months.

**Results:** A low fat free mass index <P_10_ (P_10_ FFMI), was found in 23/100 (23%) chemoradiation patients and in 17/37 (46%) patients treated with bioradiation (*p* = 0.009). Only in the chemoradiation subset, dose limiting toxicity was significantly higher in the P_10_ FFMI subgroup (*p* = 0.001). Overall, patients with low FFMI required more additional hospitalizations during CRT (*p* = 0.035). Locoregional tumour control was not impaired by low FFMI. Multivariate Cox regression analysis identified P_10_ FFMI as an independent prognostic factor for OS (HR 2.139 [95%CI 1.116–4.099], *p* = 0.022) and a prognostic advantage for LAHNSCC patients that are able to receive cisplatin (HR 0.307 [95%CI 0.157–0.600], p = 0.001).

**Conclusions:** Low FFMI (independent of BMI) is a prognostic factor for OS, treatment related toxicity and ‐tolerance in LAHNSCC patients. It also reflects significant multimorbidity, contributing to the decreased OS.

**5–11**

**The combination of bisoprolol and megace has synergistic effects on survival in a rat model of cancer cachexia**

Sandra Palus^1^, Gunter Glowalla^2^, Stephan von Haehling^1^, Andrew J.S. Coats^3^, Wolfram Döhner^4^, Stefan D. Anker^1,4^ and **Jochen Springer** ^1^

^1^ *Department of Cardiology and Pneumology, University Medical Centre Göttingen (UMG), Göttingen, Germany;* ^2^ *Institute for Biochemistry and Biophysics, Friedrich‐Schiller‐University, Jena, Germany;* ^3^ *Department of Medical Sciences, IRCCS, San Raffaele Pisana, Rome, Italy;* ^4^ *Division of Cardiology and Metabolism, Department of Cardiology (CVK); and Berlin‐Brandenburg Center for Regenerative Therapies (BCRT); German Centre for Cardiovascular Research (DZHK) Partner Site Berlin, Charité Universitätsmedizin Berlin, Germany*

**Background:** Bisoprolol and megace have both been shown to improve survival and reduce wasting in the Yoshida AH‐130 hepatoma cancer cachexia model. Here we tested a combination of both compounds.

**Methods:** the combination of 3.75 mg/kg/d bisoprolol and 75 mg/kg/d megace equaling 75% of the effective does was compared to 5 mg/kg/d bisoprolol, 100 mg/kg/d megace or placebo over a period of 16 days.

**Results:** the combination of bisopolol and megace reduced the mortality further (HR: 0.21, 95%CI: 0.11–0.44, *p* < 0.0001) than bisoprolol (HR: 0.32, 95%CI: 0.17–0.62, *p* = 0.0007) or megace (HR: 0.49, 95%CI: 0.22–1.09, *p* = 0.082) each compared to placebo. The combination was superior to singe compounds; i.e. vs bisoprolol (HR: 0.48, 95%CI: 0.13–1.79, *p* = 0.28) or vs megace (HR: 0.29, 95%CI: 0.05–1.00, *p* = 0.0489). Weight loss was attenuated by bisoprolol (−21.9 ± 10.5 g, p < 0.0001), megace (−19.2 ± 12.1 g, p < 0.0001) and the combination (−31.2 ± 8.9 g, *p* = 0.0001) vs placebo, while the differences between the treatment arms were not significant different (all *p* > 0.4). The quality of life indicators food intake was increased compared to placebo (5.3 ± 0.58) by bisoprolol (10.9 ± 2.9 *p* = 0.0003), megace (13.5 ± 2.6, p < 0.0001) or the combination (14.7 ± 1.76, p = <0.0001) on day 11. Spontaneous activity was increased by bisoprolol (43,755 ± 3,741, *p* = 0.0189), megace (51601 ± 4,000, *p* = 0.0032) or the combination (51,381 ± 2,971, *p* = 0.0009) compared placebo (33,663 ± 1,878).

**Conclusions:** The combination of bisoprolol and megace at the 75% of the effective dose, respectively increases survival and has similar effects on weight, food intake and spontaneous activity. This shows that combinations of individually effective compounds may prove beneficial for the treatment of cancer cachexia.

**5–12**

**The anti‐catabolic agent s‐oxprenolol delays disease progression and improves survival in experimental cancer cachexia**

Sandra Palus^1^, Cathleen Drescher^2^, Stephan von Haehling^1^, Andrew J.S. Coats^3^, Stefan D. Anker^1,4^ and **Jochen Springer** ^1^

^1^ *Department of Cardiology and Pneumology, University Medical Centre Göttingen (UMG), Göttingen, Germany;* ^2^ *German Institute of Human Nutrition, Potsdam, Germany;* ^3^ *Department of Medical Sciences, IRCCS, San Raffaele Pisana, Rome, Italy;* ^4^ *Division of Cardiology and Metabolism, Department of Cardiology (CVK); and Berlin‐Brandenburg Center for Regenerative Therapies (BCRT); German Centre for Cardiovascular Research (DZHK) Partner Site Berlin, Charité Universitätsmedizin Berlin, Germany*

**Background:** S‐oxprenolol is the s‐enantiomer of the unspecific beta blocker oxprenolol and has intrinsic sympathomimetic activity and effects on serotonin level in the central nervous system. Here, we tested its effect on the progression of cancer cachexia and survival in the Yoshida hepatoma model.

**Methods:** Wistar rats (approx. 200 g) were randomized to treatment with either 5 (*n* = 13), 12.5 (*n* = 21), 25 (*n* = 38) or 50 (n = 21) mg/kg/d s‐oxprenolol or placebo for 17 days. Survival, weight change, quality of life and body composition were analysed.

**Results:** Treatment with s‐oxprenolol (SOX) had no effect on tumour growth (all *p* > 0.3), which was also seen in cell culture proliferations assays (BrdU and XTT) using Kelley, HeLa‐93, and B16V cell lines. However, treatment resulted in a dose‐dependent improvement of survival: 5 mg/kg/d SOX: HR: 0.79, 95%CI: 0.39–1.57, *p* = 0.50; 12.5 mg/kg/d SOX: HR: 0.67, 95%CI: 0.38–1.17, *p* = 0.16, 25 mg/kg/d: HR: 0.56, 95%CI: 0.32–0.76, *p* = 0.0087, 50 mg/kg/d SOX: HR: 0.49, 95%CI: 0.27–0.74. *p* = 0.0075. Placebo treated animals lost a mean of −48.2 ± 1.6 g, 5 mg/kg/d SOX had no effect −48.9 ± 6.1 g (*p* = 0.87), while 12.5, 25 or 50 mg/kg/d SOX groups significantly had reduced wasting −35.3 ± 6.3 g (*p* = 0.0043), −28.7 ± 7.0 g (*p* = 0.0004) and − 17.4 ± 10.0 (p < 0.0001), respectively. The main protective effect was on lean body mass. Quality of Life indicators food intake and spontaneous locomotor activity was only assessed in the 5 and 25 mg/kg/d SOX groups. Normal food intake of a 200 g rat is approx. 22 g/d, this was reduced to 4.9 ± 0.8 g in placebo and 7.8 ± 2.4 g in 5 mg/kg/d SOX (*p* = 0.14) and 11.9 ± 2.5 g in the 25 mg/kg/d SOX group (*p* = 0.0011). Spontaneous activity was reduced to 27742 ± 2902 counts/d in the placebo group and somewhat increased by treatment with SOX 34396 ± 6428 and 36559 ± 4815 for 5 mg/kg/d (*p* = 0.23) and 25 mg/kg/d (*p* = 0.071), respectively.

**Conclusions:** S‐oxprenolol has major beneficial effects in this severe model of cancer cachexia. As the racemic oxprenolol has been used for treatment of hypertension for decades, there are excellent data on safety and therefore, s‐oxprenolol should be tested in a clinical trial.

**6–01**

**Nutritional deterioration during cancer treatment: still a reality**

**Beatriz Bartissol** ^1^, Pedro Miguel Neves^3^ and Paula Ravasco^2,3^

^1^ *Hospital de Vila Franca de Xira, Unidade de Dietética e Nutrição;* ^2^ *Hospital Universitário de Santa Maria, Universidade de Lisboa;* ^3^ *Centro de Investigação Interdisciplinar em Saúde da Universidade Católica Portuguesa, Lisboa, Portugal*

**Introduction:** Weight loss/nutritional deterioration are common features in cancer patients under active treatments. To understand whether or not their incidence is changing during treatments is of major importance, given the theoretical increased awareness in nutrition in clinical practice. Thus, this study aimed to analyse the impact of cancer treatments on Body Mass Index (BMI) and weight changes throughout the treatments.

**Methods:** Epidemiological, observational, retrospective study that included 405 patients with various types of solid tumours, submitted to chemotherapy as outpatients in Vila Franca de Xira Hospital between 2014 and 2017. Age, gender, diagnosis (location and stage), height (m) and weight (kg) at the beginning and end of the treatments was obtained from records, and BMI was calculated. Differences in weight and BMI at the beginning and end of the treatments were evaluated, as well as differences between the qualitative and quantitative variables considering cancer location and stage.

**Results:** A weight loss of 1.1% was observed in all patients (*p* < 0.001), with 39% losing more than 2.4%, as well as a reduction of 0.3 kg/m^2^ in BMI (p < 0.001). These results were more relevant for upper gastrointestinal cancer, that showed a weight loss of 4.1% (p < 0.001) and BMI reduction of 0.9 kg/m^2^ (p < 0,001), and also for stage IV disease that lost 1.9% of weight (*p* < 0.01) and had a BMI reduction of 0.5 kg/m^2^ (p < 0.01).

**Conclusions:** Despite clear advances in cancer treatment and nutritional therapy in the last decade, the prevalence of non‐intentional weight loss associated with cancer and/or treatments remains alarmingly high. Patients submitted to chemotherapy, especially those with cancer of the upper GI tract and/or advanced disease stage have significant weight loss and deterioration of BMI throughout treatments, indicating a clear need for urgent and consistent nutritional intervention. The present results from a 3 year period might translate a still ineffective nutritional intervention in cancer care.

**6–02**

**Towards a more comprehensive understanding of the barriers to implementing nutritional advice in patients with cancer cachexia**

**Rima Nasrah** ^1,2^, Christina Van der Borch^1^, Mary Kanbalian^1^ and R. Thomas Jagoe^1,2,3^

^1^ *McGill Cancer Nutrition Rehabilitation Clinic, Jewish General Hospital, Montreal, Quebec, Canada;* ^2^ *Peter Brojde Lung Cancer Centre, Jewish General Hospital, Montreal, Quebec, Canada;* ^3^ *McGill University, Montreal, Quebec, Canada*

**Introduction:** Reduced nutritional intake is recognized as a critical contributory factor in cancer cachexia. Nutritional intervention studies have frequently been hampered by poor adherence. Both physical and psychological symptoms and other possible social or environmental factors have been identified that promote poor nutrition and may act as barriers to successful nutritional intervention in patients with cachexia. However, the nature of these barriers, their prevalence and relative importance are still poorly described.

**Methods:** Patients attending a specialized multi‐disciplinary cancer cachexia clinic (McGill Cancer Nutrition Rehabilitation program at the Jewish General Hospital (CNR‐JGH)) had comprehensive symptom, nutritional and functional assessments at each visit. In addition, CNR‐JGH dietitians prospectively recorded their impression of the main barriers to successful nutritional intervention at each visit. These barriers were classified into 15 categories by similarity and subdivided into two groups: symptom‐related vs non‐symptom‐related.

**Results:** Data was collected from 114 patients over 7 months in 2017 and analysis was performed on the 94 new patients only. 89% of patients had at least one major barrier to successful nutritional intervention and non‐symptom‐related barriers were more frequent. Thus 6 non‐symptom‐related barriers accounted for 65% of all barriers identified. Barriers were dynamic and at follow up around 60% of patients had a change where new barriers became evident or old ones resolved. Simple symptom screening alone lacked specificity in detecting patients with genuine barriers to nutritional intervention. However, both patients with and those without barriers, were able to stabilize their weight over the first visit interval. The presence of barriers to nutritional intervention did not prevent increased dietary energy intake in the context of the CNR‐JGH clinic.

**Conclusions:** Close attention to non‐symptom‐related barriers to nutritional intervention is important for successful nutritional intervention in cancer cachexia. Increases in nutritional intake and stabilizing weight can be achieved with an iterative, personalized multi‐disciplinary team approach.

**6–03**

**Nutritional status and nutritional management in a patient with gastric metastasis of breast cancer: a case report**

Larissa Calixto Lima, Aline Pereira Pedrosa, **Fernanda de Oliveira Pereira** and Taiara Scopel Poltronieri

*Brazilian National Cancer Institute – INCA, Rio de Janeiro, Brazil*

**Introduction:** Gastric metastasis emergence from breast neoplasm is rare. However, the nutritional support and nutritional diagnosis is indispensable, since gastric tumours are related to nutritional complications.

**Methods:** It is a descriptive study, type case report of experience. The Patient‐Generated Subjective Global Assessment (PG‐SGA),weight, height, triceps skinfold (TSF), arm circumference (AC), arm muscle circumference (AMC) and laboratory tests (complete blood count, C‐reactive protein and albumin) were analysed and the glasgow prognostic score (GPS) was calculated.

**Results:** A 54‐year‐old female, Caucasian, diagnosed with invasive lobular adenocarcinoma of the left breast, stage IV (progression to bones, ovary and stomach), grade II, presenting positive hormonal receptors, HER‐2 negative. Initially, she was classified as having a moderate malnutrition (PG‐SGA B), indicating a need of nutritional intervention. In the anthropometric evaluation, the patient weighed 74.5 kg, her height was 157 cm and presented BMI's indicative of obesity grade I (30.2 kg/m^2^) but the percentiles of TSF, AC and AMC were adequate. She had odynophagia, anorexia and severe weight loss of 2,7%, in a week, and total weight loss of 9.4% in one month. The laboratory tests showed hypoalbuminemia, thrombocytopenia and anaemia while the relationship between serum levels of C‐reactive protein and albumin showed GPS equal to zero. For all this, according to Fearon, this patient was classified as being in a pre‐cachexia stage. Due to gastrointestinal symptoms and the pre‐cachexia stage, it was opted for nasoenteric tube after discussion with an interdisciplinary team, but the patient died three days late. According to the GPS the prognosis was low‐risk, but, since death is not a probabilistic event, the precise time of its occurrence cannot be defined by prognostic factors.

**Conclusions:** The evaluations used daily in clinical practice such as significant weight loss, loss of appetite and metabolic changes can be used easily to classify the cachexia but the best use of nutritional therapy in these patients is still a dilemma among professionals.

**Table 5:** Anthropometric and biochemical evaluation comparing admission values and the last nutritional evaluation of the patient with gastric metastasis of breast cancer.

ANTHROPOMETRIC PARAMETERS	**ADMISSION** **(06/07/2017)**	**NUTRITIONAL REVALUATION (14/07/2017)**	**CLASSIFICATION**	**REFERENCES**
**Weight (kg)**	74,5	72,5	**	**
**Height (m)**	1,57	1,57	**	**
**BMI (kg/m** ^**2**^ **)**	30,2	29,4	Overweight	OMS (1998)
**WL (%)**	**	2,7% in a week	WL severe	Blackburn *et al*. (1977)
**AC (cm)**	**	31,9 (p50‐p75)	Adequate	Frisancho (1990)
**TSF (mm)**	**	28 (p50‐p75)	Adequate	Frisancho (1990)
**AMC (cm)**	**	22,2 (p25‐p50)	Adequate	Frisancho (1981)

**BIOCHEMICAL DATA**	**ADMISSION** **(06/07/2017)**	**NUTRITIONAL REVALUATION (14/07/2017)**	**CLASSIFICATION**	REFERENCES*
**HAEMATOCRIT (%)**	38,5	25,9	Low	36–47
**HAEMOGLOBIN (g/dL)**	13,0	8,50	Low	11.5–16.4
**LEUCOCYTES (/μl)**	6900	5200	Adequate	4000–10000
**PLATELETS (k/μl)**	140	93	Low	150–400
**C‐REACTIVE PROTEIN (mg/L)**	Not required	8,64	High	<0,5
**ALBUMIN (g/dL)**	3,4	2,4	Low	3,5‐5,2
**GPS**	**	0	Low risk	McMillan (2008)

BMI: Body Mass Index; WL: Weight Loss; AC: Arm Circumference; TSF: triceps skinfold; AMC: arm muscle circumference; GPS: Glasgow Prognostic Score; *Limits adopted by the laboratory of the Hospital of Cancer III ‐ INCA; **Not applicable

**6–04**

**miRNA‐130a significantly improves accuracy of SGA nutritional assessment tool in prediction of malnutrition and cachexia in radiotherapy treated head and neck cancer patients**

**Tomasz Powrózek** ^1^, Radosław Mlak^1^, Anna Brzozowska^2^, Marcin Mazurek^1^, Paweł Gołębiowski^2^ and Teresa Małecka‐Massalska^1^

^1^ *Department of Human Physiology, Medical University of Lublin, Poland;* ^2^ *Department of Oncology, Medical University of Lublin, Poland*

**Background:** Investigation of novel cachexia related markers is a one of the major challenges in contemporary oncology. Among studied markers, the miRNA seems to be promising due to its possibility to regulate genes responsible for induction of inflammatory response, muscle atrophy and fat tissue wasting.

**The aim of the study** was to investigate the role of blood circulating miRNA‐130a in prediction of cancer cachexia in 70 head and neck cancer patients (HNC) subjected to radiotherapy. Moreover, diagnostic accuracy of SGA scoring and miRNA‐130a level was evaluated in various cachexia models.

**Results:** miRNA‐130a level negatively correlated with plasma TNF‐α concentration(*r* = −0.560; p = <0.001). Patients with low miRNA expression had over 3‐fold higher risk of BMI decrease below 18.5 after the termination of therapy; over 6‐fold higher risk of losing over 5% of body weight and higher risk of >10% weight reduction (OR = 14.18) compared to other cases. ROC analysis performed for miRNA‐130a allowed to distinguish cachectic patients (body weight loss >5%) from moderately or mildly malnourished ones with optimal sensitivity of 79.4% and specificity of 80.8% (AUC = 0.865). miRNA significantly improved nutritional assessment conducted using SGA, achieving the following values: sensitivity – 88.6%, specificity – 94.3%, PPV – 93,9%, NPV – 89.2%.

**Conclusions:** miRNA‐130a demonstrates potential clinical utility in prediction of cachexia prior to the therapy in HNC patients. Simultaneous use of both tools ‐ SGA and miRNA significantly improved the accuracy in the diagnosis of cachexia.

**6–05**

**Dietary potassium intake and mortality in hemodialysis patients**

Yoko Narasaki, Yusuke Okuda, Amy S. You, Tracy Nakata, Elani Streja, Kamyar Kalantar‐Zadeh and **Connie M. Rhee**

*Harold Simmons Center for Chronic Disease Research and Epidemiology, Division of Nephrology and Hypertension, University of California, Irvine, Orange, CA, USA*

**Background:** While clinical practice guidelines advise dietary potassium restriction among dialysis patients, given concerns about potential hyperkalemia, there are sparse data informing these recommendations. While one prior study has observed that higher dietary potassium intake was associated with higher mortality risk in hemodialysis patients, inference from these findings are limited by lack of key covariate data (e.g., dialysate potassium concentration).

**Methods:** Among 415 hemodialysis patients from the multi‐center prospective “Malnutrition, Diet, and Racial Disparities in Chronic Kidney Disease” study, information regarding dietary potassium intake was obtained using the Block Food Frequency Questionnaire administered over the period of October 2011–March 2015. Associations of baseline dietary potassium intake categorized as tertiles with all‐cause death risk were examined using unadjusted, case‐mix adjusted (age, sex, race, ethnicity, and diabetes status), and expanded case‐mix adjusted (case‐mix covariates + vintage, vascular access, insurance, congestive heart failure, coronary artery disease, coronary artery disease/cerebrovascular accident, combined cardiovascular disease disease, BMI, and dialysis potassium concentration) Cox analyses.

**Results:** The mean ± SD age of the cohort was 56 ± 15 years, among whom 45%, 48%, and 36% were female African‐American, and Hispanic, respectively. In unadjusted, case‐mix, and expanded case‐mix adjusted analyses, the lowest tertile of dietary potassium intake was associated with higher mortality risk (ref: highest tertile): HRs (95% CIs) 1.54 (1.03,2.30), 1.68 (1.11,2.55), and 1.74 (1.14,2.66), respectively.

**Conclusions:** In a prospective cohort of hemodialysis patients, dietary potassium intake in the lowest tertile was associated with higher death risk. These findings suggest that excessive dietary potassium restriction may be deleterious in dialysis patients, and further studies are needed to determine the optimal dietary potassium intake in this population.

**6–06**

**Short‐term creatine supplementation reduces the malnutrition‐inflammation score and attenuates lean body mass loss in hemodialysis patients**

**Gustavo Pimentel** ^1^, Ana Marini^1^, Reika Motobu^1^, Ana Freitas^1^, Joao Mota^1^, Fabio Lira^2^, Alessandro Laviano^3^ and Benjamin Wall^4^

^1^ *Federal University of Goiás Goiânia, Brazil;* ^2^ *São Paulo State University;* ^3^ *Sapienza University, Rome, Italy;* ^4^ *University of Exeter, UK*

**Introduction:** Hemodialysis (HD) induces an imbalance between muscle protein synthesis and breakdown, leading to loss of muscle mass and function. Creatine supplementation has been proposed as s nutritional strategy to alleviate muscle loss in various populations. But has not been investigated in chronic kidney disease patients undergoing HD. Thus, our objective was to evaluate whether creatine supplementation could attenuate the loss of lean body mass (LBM) and malnutrition‐inflammation score (MIS) in patients undergoing HD.

**Methods:** A randomized, placebo‐controlled, double blind, parallel‐design study included patients undergoing HD, of both sexes, aged 18–59 years. The patients were randomly divided into a Placebo Group (*n* = 14; who received maltodextrin, 20 g for four weeks) and a Creatine Group (n = 14; who received creatine supplementation, 20 g in the first week and 5 g at 2nd‐4th week, for four weeks). Before and after the study period, all volunteers were evaluated for food intake and, MIS, and body composition was determined using DXA and inelastic tape.

**Results:** Creatine supplementation attenuated the MIS (Pre:5.57 ± 0.72 vs. Post:1.79 ± 0.47 score, *p* = 0.003) compared with the placebo (Pre:5.71 ± 0.97 vs. Post:5.36 ± 0.95 score, *p* = 0.317) (ANOVA *p* = 0.017, effect size:0.09). Creatine supplementation was able to increase LBM (Pre:42.96 ± 2.74 vs. Post:43.92 ± 2.71 kg, *p* = 0.009) when compared to placebo group (Pre:41.33 ± 2.28 vs. Post:41.46 ± 2.36 kg, *p* = 0.550) (ANOVA *p* = 0.038, effect size:1.41). Moreover, the creatine group presented lower % fat mass (Creatine ∆:‐0.62% vs. Placebo ∆:+0.53%, *p* = 0.011) and higher thigh (Creatine ∆:+0.35 vs. Placebo ∆:‐0.15 cm, *p* = 0.000) and calf circumferences (Creatine ∆ = +0.27 vs. Placebo ∆:‐0.96 cm, *p* = 0.031) and gait speed (Creatine ∆:+0.05 vs. Placebo ∆:‐0.03meters/sec, *p* = 0.035) than placebo group. Food intake did not change among the groups.

**Conclusions:** In HD patients, creatine supplementation may to reduce the malnutrition‐inflammation score, as well as attenuate the loss of LBM, fat mass, thigh and calf circumferences and gait speed when compared to placebo group.

**6–07**

**Dietary factors for preventing sarcopenia in the elderly Japanese population**

**Yasumi Kimura** ^1^ and Shuji Akagi^2^

^1^ *Department of Nutritional Sciences, Faculty of Nutritional Sciences, Nakamura Gakuen University, Fukuoka, Japan;* ^2^ *Department of Human Nutrition, Faculty of Contemporary Life Science, Chugoku Gakuen University, Okayama, Japan*

**Introduction:** The proportion of elderly people in the Japanese population is increasing rapidly, and dietary factors have gained attention for their effects on healthy life span and preventive care. Sarcopenia, which involves loss of muscle mass and strength and symptoms such as whole body muscle weakness and decline in physical function, is one of the most important factors contributing to the requirement for nursing care. Thus, preventive management of sarcopenia is increasingly important for the elderly to achieve a healthy and independent lifestyle. The aim of this study was to clarify the dietary factors associated with sarcopenia in elderly subjects.

**Methods:** Participants were community‐dwelling subjects aged 65 or over (*n* = 183, 46 men and 137 women). Sarcopenia was assessed using the algorithm of the Asian Working Group for Sarcopenia (AWGS). Body height and weight were measured, and BMI was calculated by dividing weight by squared height (kg/m^2^). Dietary intake was evaluated using a brief‐type self‐administered diet history questionnaire (BDHQ). All selected food and nutrient intakes were adjusted for energy by the density method. IBM SPSS Statistics 22 was used for statistical analysis.

**Results:** Of the 183 study subjects, 20 had sarcopenia. Compared with the non‐sarcopenia group, the sarcopenia group were older, had lower BMI and significantly decreased food intake. The sarcopenia group also had significantly higher intake of carbohydrate and lower intake of total energy, protein and vitamin D. Regarding foods, the sarcopenia group had lower intake of cooking oil, salt and beer.

**Conclusions:** Protein and vitamin D have previously been reported to be the most important nutrients for preventing sarcopenia, and both of these were consumed in lower amounts in the sarcopenia group in the present study. Our results also indicate that having adequate food intake and appropriate carbohydrate intake may contribute to sarcopenia prevention.

**6–08**

**Failure of early, evidence based intensive care unit nutrition in preventing sepsis induced chronic critical illness**

**Martin Rosenthal**, Scott Brakenridge, Trina Bala, Zhongkai Wang and Frederick Moore

*University of Florida, Gainesville, USA*

**Introduction:** Sepsis has plagued ICUs for decades. With recent advances, early sepsis mortality is now low (< 10%), but unfortunately many survivors progress into a new chronic critical illness (CCI) cachexia phenotype. The purpose of this study was to determine how CCI patients responded to receiving early adequate ICU nutrition by an evidenced based protocol (EBP).

**Methods:** A retrospective review on an USA NIH funded sepsis database identified 22 CCI patients (defined as ICU days >14 with organ dysfunction) who received early adequate nutritional per EBP (receiving >80% nutritional goals) and these were compared to 44 matched rapid recovery (RAP) patients (discharged <14 days with no organ dysfunction).

**Results:** By matching the CCI and RAP groups had similar baseline characteristics but CCI patients had higher serial SOFA scores and experienced more nosocomial infections. Comparative serial biomarkers over 4 weeks (all *p* < 0.05), showed that CCI patients remained persistently inflamed with ongoing stress metabolism (higher IL‐6, IL‐8 levels, and GLP‐1 p < 0.05), that despite receiving adequate EBP nutrition, they had evidence of persistent catabolism (higher urine 3‐methylhistadine excretion), and persistent immunosuppression (higher neutrophil to lymphocyte ratio with higher soluble PDL‐1 levels). More CCI were discharged to non‐home destinations (81% vs 29%, p < 0.05). Functional status of CCI patients was worse by Zubrod score (3.25 vs 1.40, p < 0.05), and the CCI cohort had lower 12‐month survival (73% vs 97%, p < 0.05).

**Conclusions:** Despite early adequate EBP nutrition septic ICU patients who develop CCI exhibit: 1) persistent inflammation, 2) poor nutritional status, 3) poor functional status and 4) much worse 12 month survival. We concluded EBP nutrition needs to be modified to improve outcomes for this vulnerable patient population. Nutrients that lessen inflammation (e.g. resolvens) and promote anabolism (e.g. leucine or propranolol) combined with resistance exercise warrant investigation.

**6–09**

**The effect of the fish oil metabolite docosahexaenoyl‐serotonin on hypothalamic inflammation**

**Klaske van Norren**, Sebastian Eriksson and Mieke Poland

*Human Nutrition, Wageningen University, The Netherlands*

**Introduction:** Hypothalamic inflammation has been reported to play a key role in cancer cachexia. The gut might accelerate the influence of hypothalamic inflammation on cancer‐ or more in general disease‐induced muscle wasting. The objectives of this study were to: 1) determine the role of gut‐flora derived compounds like lipopolysaccharide (LPS) on hypothalamic inflammation in vitro and 2) to investigate if docosahexaenoic acid (DHA) and the DHA derivative docosahexaenoyl serotonin (DHS) can inhibit this process.

**Methods:** In this study we tested the effect of docosahexaenoic acid (DHA), serotonin and the acyl‐amine docosahexaenoyl‐serotonin (DHA‐serotonin) on hypothalamic inflammation in vitro. The effect on a co‐culture of hypothalamic HypoE‐46 cells and BV‐2 microglial cells was compared to the effect on monocultures of these two cell lines. Microglial cells were seeded on top of hypothalamic cells that had grown for 24 h. Inflammation was initiated with the addition of 10 to 31,6 ng/mL LPS. These low concentrations of LPS resemble the physiological concentrations in the hypothalamus. At the same time point that LPS was added, DHA‐serotonin was added at different concentrations. IL‐6 and MCP‐1 excretion was measured with ELISA. LDH, BCA and WST‐1 assays were performed to exclude toxicity.

**Results:** LPS stimulated IL‐6 and MCP‐1 release in the hypothalamic cells. IL‐6 and MCP‐1 release were higher in the co‐culture (180% and 220% respectively). These LPS concentrations had no influence on cytokine excretion of the microglial cell monoculture. Addition of 0.3 uM DHA‐serotonin decreased the IL‐6 release in the co‐culture with 25% (p < 0.05), but not in the hypothalamic monoculture. The combination of serotonin and DHA (control) at the same concentrations had no effect. MCP‐1 release was not affected by the addition of DHA‐serotonin.

**Conclusions:** These data indicate that cytokine production is different in the co‐culture when compared to the mono‐cultures, suggesting a sensing of the inflammation by the hypothalamic cells and a moderating effect by the microglial cells. DHA‐serotonin has potential for reducing this hypothalamic inflammation.

**6–10**

**Leucine nutritional supplementation modulates the tumour‐induced alteration in energy metabolism and production in skeletal muscle activity**

Bread Cruz^1^, Daniel Martins‐de‐Souza^2^ and **Maria Cristina Cintra Gomes‐Marcondes** ^1^

^1^ *Department of Structural and Functional Biology, Laboratory of Nutrition and Cancer;* ^2^ *Department of Biochemistry and Tissue Biology, Biology Institute, University of Campinas (UNICAMP), Brazil*

**Introduction:** Leucine can maintain lean body mass by stimulating muscle protein synthesis and inhibiting proteolysis. Therefore, we evaluated in skeletal muscle the proteomic compared to metabolomic profiles (*in vivo* vs *in vitro* assays) under leucine and/or tumour effects.

**Methods:** Wistar rats were distributed in four groups: Control (C), Walker tumour‐bearing (W), leucine‐rich diet (L) and tumour‐bearing subjected to leucine‐rich diet (LW) to access the proteomic profile in gastrocnemius muscles. C2C12 cells treated with leucine (50uMol) and/or Walker factor (WF; 25 μg/well) for 24 h, were accessed the metabolomic profile analysis by ^1^H‐NMR spectroscopy, following the bioinformatics tool Metaboanalyst (analysed using http://www.metaboanalyst.ca). The proteomic profile of muscles samples in Walker‐256 tumour‐bearing rats was analysed by STRING v10 software.

**Results:** Muscles and myotube cells from W and LW groups showed 15 metabolites with statistical difference associated with important metabolic pathway changes in LW group. The main altered metabolites present in higher concentration in the LW group were glutamine, proline, lactate, aspartate (*P* < 0.05), which led to impacted metabolic pathways: ammonia recycling; urea cycle; amino acids metabolism; protein biosynthesis and gluconeogenesis (P < 0.05). We observed constant modulation of protein metabolism and amino acids, as well as processes related to energy biosynthesis in LW group, showing that leucine supplementation leads to significant changes in cellular metabolism, especially under the Walker factor exposure. In addition, the metabolomic and proteomic results showed that the main cellular component affected was mitochondria, especially in catalytic activity, ion binding and electron carrier activity. The principal biological processes modulated by leucine were the energy generation, tricarboxylic acid cycle and carbohydrates catabolic processes. The metabolomic analyses of myotubes corroborated the proteomic results in muscle tissue.

**Conclusions:** Our study showed beneficial effects of leucine supplementation (metabolomic and proteomics analyses), providing some information about the muscle glycolytic metabolic pathways under tumour‐induced damages.

**Support:** FAPESP; CNPq; FAEPEX).

**6–11**

**Lipid‐rich parenteral nutrition or parenteral nutrition supplemented with ketone bodies attenuate critical illness‐induced muscle weakness in lean mice**

**Chloë Goossens**, Sarah Derde, Sarah Vander Perre, Greet Van den Berghe^*^ and Lies Langouche^*^

*Clinical Division and Laboratory of Intensive Care Medicine, Department of Cellular and Molecular Medicine, KU Leuven, Leuven, Belgium*

^*^equal contribution

**Introduction:** We previously documented that obese critically ill patients are protected against debilitating critical illness‐induced muscle wasting and weakness. In mice, we could attribute this protective effect to a more pronounced lipolysis in obese compared to lean critically ill. Here, we assessed whether increased lipid availability in lean ill mice would similarly attenuate muscle wasting and weakness.

**Methods:** We used a centrally‐catheterized, fluid‐resuscitated, antibiotic‐treated mouse model of prolonged (5 days) critical illness evoked by abdominal sepsis (*n* = 15–23 mice/group). In experiment 1, we compared markers of muscle wasting and weakness, fatty acid oxidation, and ketogenesis in lean septic mice receiving standard parenteral nutrition (standard‐PN; 46% glucose‐, 35% lipid‐, 16% protein‐derived kcal) or lipid‐rich PN (lipid‐PN; 90% lipid‐, 10% glucose‐derived kcal). In experiment 2, we compared markers of muscle wasting and weakness in lean septic mice receiving standard‐PN supplemented with glucose (PN + gluc; 6.25 mg/g/day) or with D,L‐3‐hydroxybutyrate (PN + 3HB; 5 mg/g/day). Pair‐fed healthy mice served as controls.

**Results:** In experiment 1, muscle mass was reduced with 29% in both standard‐PN and lipid‐PN septic mice (*p* ≤ 0.001). However, lipid‐PN septic mice maintained specific maximal muscle force up to healthy control levels (*p* = 0.6), whereas this was reduced with 27% in standard‐PN septic mice (*p* = 0.006). Furthermore, lipid‐PN enhanced hepatic and muscular fatty acid oxidation (*p* ≤ 0.05) and stimulated ketogenesis (*p* < 0.0001), but also caused hepatic triglyceride accumulation and steatosis (p < 0.0001). Also in experiment 2, muscle mass was reduced with 25% in both ill groups (p < 0.0001). Similar to lipid‐PN, PN + 3HB attenuated the reduction in specific maximal muscle force compared to PN + gluc (p = 0.01), again up to healthy control levels (*p* = 0.1). Importantly, hepatic triglyceride accumulation was absent in PN + 3HB septic mice.

**Conclusions:** Lipid‐rich PN protected lean septic mice against the muscle weakness, but not the muscle wasting. Improved ketogenesis appeared essential herein as 3HB supplementation of PN similarly protected against critical illness‐induced muscle weakness.

Funding: GVdB and LL, via the KU Leuven, receive long‐term structural research support from the Methusalem Program funded by the Flemish Government (METH14/06) and from the Research Foundation Flanders (G.0C78.17 N). This project has received funding from the European Research Council (ERC) under the European Union's Horizon 2020 research and innovation program [AdvG 2017–785809].

**6–12**

**Nutritional intervention attenuates skeletal muscle alterations in a mouse model of pulmonary arterial hypertension**

**Paulien Vinke** ^1,3^, T. Scott Bowen^2^, Renger F. Witkamp^3^, Volker Adams^*, 4^ and Klaske van Norren^*, 3^

^1^ *University Clinic of Cardiology, Heart Center Leipzig, Leipzig, Germany;* ^2^ *School of Biomedical Science, University of Leeds, Leeds, United Kingdom;* ^3^ *Nutrition and Pharmacology Group, Division of Human Nutrition and Health, Wageningen University, Wageningen, the Netherlands;* ^4^ *Department of molecular and experimental cardiology, TU Dresden, Heart Center Dresden, Dresden, Germany*

^*^Contributed equally

**Introduction:** Alterations in skeletal and respiratory muscles contribute to exercise intolerance and fatigue in pulmonary arterial hypertension (PAH), a chronic disease mostly affecting women. Muscle atrophy and fibre shifts towards a fatigable phenotype in limb and respiratory muscles in models of PAH remain poorly resolved, particularly in relation to potential benefits of nutritional intervention. As such, the present study administered a nutritional intervention with high protein, leucine, fish oil and oligosaccharides to determine whether skeletal muscle alterations could be attenuated in a female mouse model of PAH.

**Methods:** Female mice (C57/BL6) were treated for 8 weeks with saline (sham; *n* = 10) or monocrotaline (MCT; 600 mg/kg; n = 10) to induce pulmonary arterial hypertension in combination with a control diet (standard chow AIN‐93 M), while a further MCT‐treated group received a nutritional intervention (NI, isocaloric) (MCT + NI; n = 10). Muscle wet weights for diaphragm, tibialis anterior (TA), soleus and EDL were measured and subsequent histological analyses were performed.

**Results:** Compared to shams, MCT mice showed an increase in heart weight, right ventricular thickness and fibrosis (all p < 0.05), all signs of pulmonary arterial hypertension. Wet weights of TA, soleus and EDL were not different between all groups. Further analyses revealed fibre cross‐sectional area of the diaphragm was also not different between all groups. In contrast, however, the limb muscle TA fibre cross‐sectional area was reduced (P < 0.05) by 22% in MCT compared to sham mice, but preserved in the MCT + NI group.

**Conclusions**: Nutritional intervention attenuates limb skeletal muscle alterations in a mouse model of pulmonary arterial hypertension. Interestingly, greater deficits were observed in limb compared to respiratory muscle. Further study is therefore warranted to determine why limb and respiratory muscle are differentially impacted during PAH, but these data provide novel evidence that nutritional intervention with high protein, leucine, fish oil and oligosaccharides can prevent muscle wasting in PAH.

